# Historical and contemporary factors generate unique butterfly communities on islands

**DOI:** 10.1038/srep28828

**Published:** 2016-06-29

**Authors:** Raluca Vodă, Leonardo Dapporto, Vlad Dincă, Tim G. Shreeve, Mourad Khaldi, Ghania Barech, Khellaf Rebbas, Paul Sammut, Stefano Scalercio, Paul D. N. Hebert, Roger Vila

**Affiliations:** 1 Institut de Biologia Evolutiva (CSIC-Universitat Pompeu Fabra), Passeig Marítim de la Barceloneta 37, 08003, Barcelona, Spain; 2 Department of Life Sciences and Systems Biology, University of Turin, Via Accademia Albertina 13, 10123 Turin, Italy; 3 Department of Biology, University of Florence, 50019 Florence, Italy; 4 Centre for Biodiversity Genomics, Biodiversity Institute of Ontario, University of Guelph, Guelph, N1G 2W1, Ontario, Canada; 5 Department of Biological and Medical Sciences, Oxford Brookes University, Headington, Oxford, OX3 0BP, UK; 6 Département d’Agronomie, Université Mohamed Boudiaf de M’sila, 28000 M’sila, Algeria; 7 Département des sciences de la nature et de la vie, Université Mohamed Boudiaf de M’sila, 28000 M’sila, Algeria; 8 137, “Fawkner/2” Dingli Road, Rabat RBT 9023, Malta; 9 Consiglio per la ricerca in agricoltura e l’analisi dell’economia agraria, Unità di Ricerca per la Selvicoltura in Ambiente Mediterraneo, c.da Li Rocchi, I-87036 Rende (CS), Italy

## Abstract

The mechanisms shaping island biotas are not yet well understood mostly because of a lack of studies comparing eco-evolutionary fingerprints over entire taxonomic groups. Here, we linked community structure (richness, frequency and nestedness) and genetic differentiation (based on mitochondrial DNA) in order to compare insular butterfly communities occurring over a key intercontinental area in the Mediterranean (Italy-Sicily-Maghreb). We found that community characteristics and genetic structure were influenced by a combination of contemporary and historical factors, and among the latter, connection during the Pleistocene had an important impact. We showed that species can be divided into two groups with radically different properties: widespread taxa had high dispersal capacity, a nested pattern of occurrence, and displayed little genetic structure, while rare species were mainly characterized by low dispersal, high turnover and genetically differentiated populations. These results offer an unprecedented view of the distinctive butterfly communities and of the main processes determining them on each studied island and highlight the importance of assessing the phylogeographic value of populations for conservation.

Island communities share several features: the number of species is lower than on similarly sized mainland areas (impoverishment), they host a disproportionate fraction of non-predatory and highly dispersive species (disharmony) and they are characterized by a high fraction of endemic elements^1^. In the last decades it has been largely recognized that these features are shaped by colonization and extinction events but also by the long-term persistence of populations, resulting in relictuality and endemicity^1^,^2^,^3^. In turn, these processes depend on a large number of factors related to both species and island characteristics, as well as to stochastic events^1^,^4^. As a result, no island populations and communities are identical and they have been regarded as “individuals”^5^. Hence, recognizing the main drivers behind the formation of each community and developing specific conservation plans require in-depth comparative analyses linking community and genetic approaches^6^,^7^,^8^.

An important property of island communities is that their structure tends to be nested, with some species occurring on most islands, and others occurring on fewer islands, usually the largest and least isolated^9^,^10^. The existence of nested patterns has important implications for conservation; when communities are highly nested, conservation decisions are simplified and the usual strategy is to concentrate efforts on the most diverse communities^1^. After a series of studies suggested that nested structures universally occur within different taxa and island systems^11^, the recent introduction of strict null models revealed that significantly nested patterns are less common than previously hypothesized^12^. There is also a debate^6^,^13^ as to whether species occurrence in island communities is mostly determined by their frequency in neighbouring source areas, as postulated by neutral theories^2^,^14^, or whether competition and interaction between species traits and island characteristics (mostly mediated by differences in dispersal tendency among species) play a primary role^3^,^10^,^15^.

Genetic variation provides fundamental information to understand the historical and ecological dynamics that shaped island populations and communities^6^,^16^. Past connections or long-term isolation are well-known determinants of phylogeographic patterns, but they exert variable effects on different species, with highly migratory species expected to show the least geographic variation due to frequent gene flow^16^,^17^,^18^. However, the assumption that species lacking spatial genetic differentiation are the most vagile and widespread has rarely been tested^6^,^16^,^19^.

Following these premises, it is not surprising that, although numerous studies exposed the role of complex phenomena in producing faunistic^20^,^21^ and genetic community structures for selected insular taxa^19^,^22^, comprehensive studies aiming to disentangle large arrays of ecological-historical and deterministic-stochastic factors over large taxonomic groups are uncommon^8^,^16^,^17^. In this study, we examined butterfly communities occurring on the circum-Sicilian islands and compared them with populations from the southern Italian Peninsula, Sicily and north Africa. Sicily and the surrounding islands represent a biogeographic crossroad with high species richness and contrasting biodiversity^23^,^24^,^25^. These islands have different geological histories and locations with respect to the two main faunistic sources (southern Europe and north Africa) and possess different environmental settings, while the well-known taxonomy and distribution of the butterflies occurring in this region make them an excellent model system. However, the sparse information about their genetic structure has impeded the link between community composition and patterns of genetic differentiation. In this study we: i) model species richness over the western Mediterranean islands, ii) examine the pattern of nestedness in the study area, iii) analyse the relative importance of species dispersal tendency and frequency at source in determining their frequency on islands, iv) we sequenced the COI gene to assess the genetic differentiation patterns for populations of 29 species and test for a possible correlation between dispersal tendency and regional genetic variation, v) document the overall genetic differentiation pattern for north Africa, circum-Sicilian islands, Sicily, and the Italian Peninsula, and vi) categorize the species based on their island occupancy and genetic structure. This integrative approach aids the recognition of the multiple processes generating species assemblages for an entire and diverse superfamily and provides the information needed to prioritize conservation decisions in a key biogeographical contact zone.

## Results

### Determinants for island species richness

The AIC-based Generalized Linear Model (GLM) for the richness of Western Mediterranean islands on the basis of ecological factors, returned a model with four variables: island area (IA), isolation (IS), source richness (SR) and maximum elevation (EL). Isolation represented the largest part (26.1%) of the total variance explained by the model (67.8%) (Supplementary Table S3). The Maltese islands, Lampedusa, Lipari and Vulcano were richer in species than expected (Supplementary Fig. S1a), while Pantelleria, Linosa, Stromboli and Marettimo had fewer species than expected. In the second GLM (including the Pleistocene land connections (PC) as a variable) a six variable model best fitted the data (Supplementary Table S4), adding mean island precipitation (PT) and PC to the previous model. In order to make this model directly comparable with the first one, we excluded PT (results including this variable are shown in Supplementary Table S4). This model increased the explained variance to 73.2% with PC having the lowest *lmg* value (Table 1). The difference in richness residuals between the model without the PC and the full model can provide an estimation of the number of species that colonized the islands during the Last Glacial Maximum (LGM), due to land-bridge connections, and survived until present (Table 2).

### Community structure

**Figure 1:**
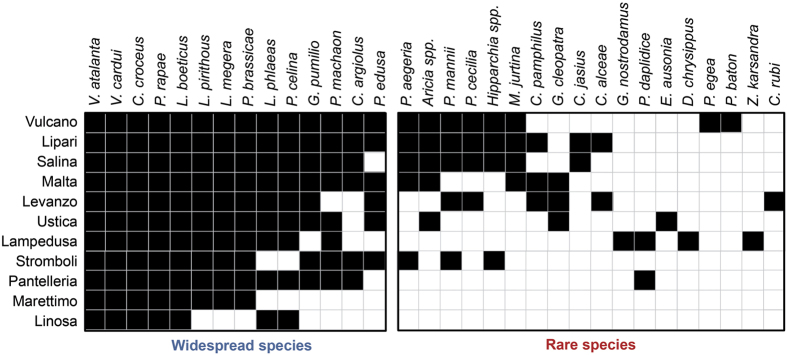
The packed matrix minimizing the NODF metric for the butterfly species recorded on the islands examined in this study. The rows represent the studied islands and the columns the species occurring in each island. Widespread species showed a much more nested pattern than rare ones.

**Figure 2:**
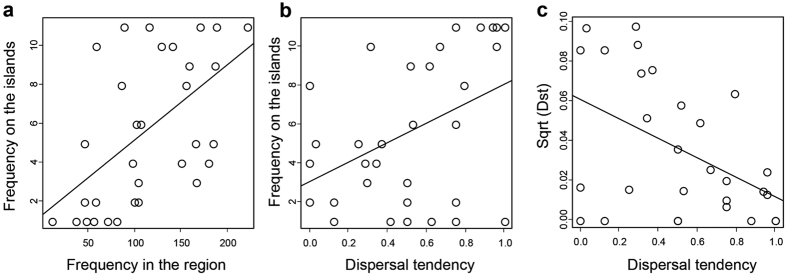
(a) The correlation between species occurrence on islands and their frequency at source in cells of 0.1 × 0.1 latitude-longitude degrees. **(b)** The correlation between species occurrence on islands and their dispersal tendency. **(c)** The correlation between genetic variation (Dst) and dispersal tendency.

Various null models provided different evidence for the existence of a significantly nested pattern of the butterfly communities on the 11 studied islands. The observed nestedness metric based on overlap and decreasing fill (NODF) of the entire packed matrix (Fig. 1) was significantly higher than the mean NODF obtained with the equiprobable rows and columns (EE) and proportional rows and columns total (CE) null models (Table 3). However, the mean NODF for the fixed rows and columns (FF) null model was significantly higher than the observed value, revealing an anti-nested pattern (Table 3). After dividing the matrix into two sub-matrices corresponding to widespread and rare species (occurring in more and less than half of the islands, respectively), widespread species showed a nested pattern for the EE and CE null models, while rare species showed a significantly anti-nested pattern with the FF model (Table 3). In particular, species occurring on less than four islands showed a highly chequered distribution (Fig. 1). Accordingly, when all species were considered, nestedness and turnover components had similar contributions in determining faunistic dissimilarities with a ratio between nestedness and Sørensen (nest/sor) indexes of 0.588. When divided into sub-matrices, the widespread species showed a predominance of the nestedness component (nest/sor = 0.814), and the rare ones a predominance of species replacement (nest/sor = 0.232). A multiple regression showed that species frequency on islands was significantly associated with both frequency at source (n = 32, Est. = 0.037, t = 3.88, P < 0.001, Fig. 2a, Supplementary Fig. S2a) and with dispersal tendency (n = 32, Est. = 4.607, t = 2.84, P = 0.008, Fig. 2b, Supplementary Fig. S2b). The partitioning of variance revealed that dispersal tendency explained a lower fraction of variation than frequency at source (16.4% for dispersal tendency and 29.5% for frequency at source).

### Genetic pattern

**Figure 3:**
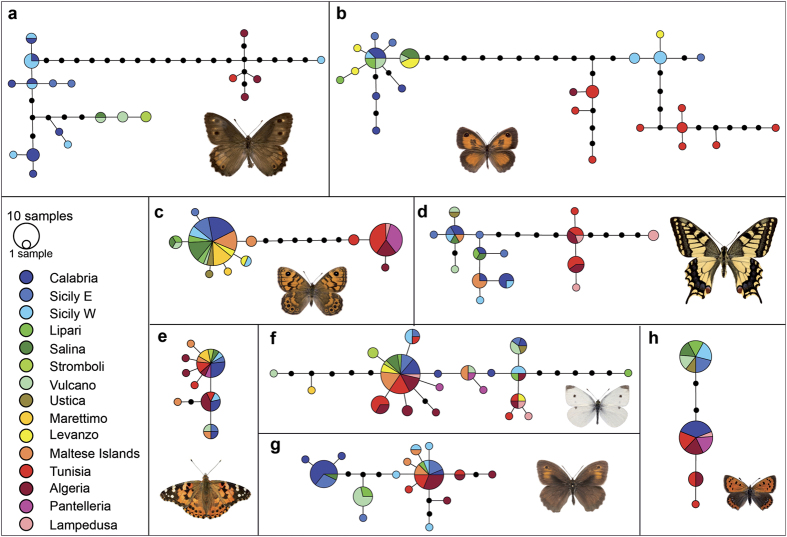
COI haplotype networks for eight of the 27 investigated species (the other species are represented in Supplementary Note). **(e**,**f)** Species with genetic variation but no spatial segregation (*V. cardui*, *P. rapae*, respectively); **(a**,**b**,**g)** species with spatial differentiation but no mutually exclusive pattern among lineages (*Hipparchia spp.*, *P. cecilia* and *M. jurtina*, respectively); **(d**,**h**,**c)** species showing evidence of mutual exclusion (*P. machaon*, *L. phlaeas* and *L. megera*, respectively). Some of these species also have endemic lineages. Photographs of the butterfly species: **(a–d, f–h)** R. Vila; **(e)** V. Dincă.

**Figure 4:**
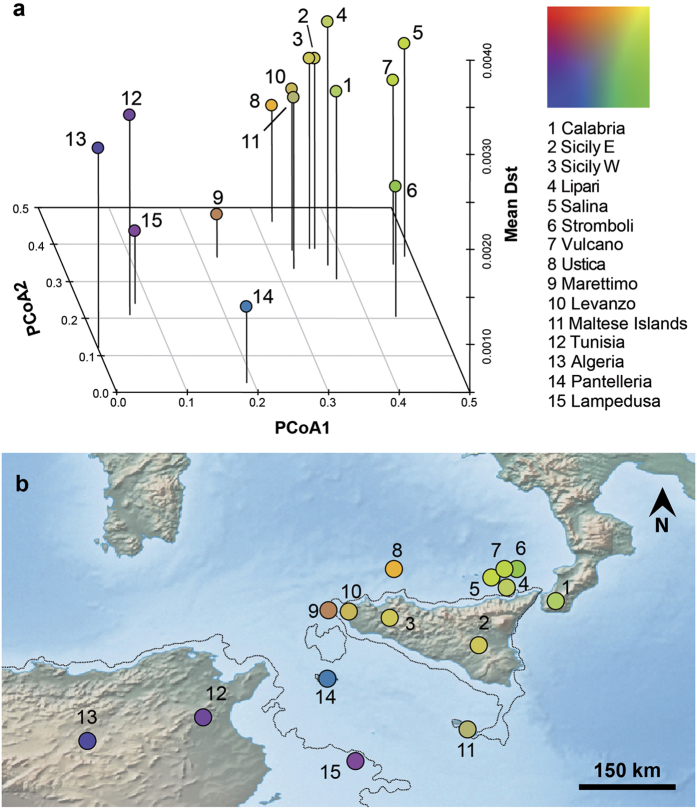
(a) Principal Coordinates Analysis (PCoA) showing the pattern of similarity among islands based on the mean population differentiation (Gst) among the 27 studied species for the 15 areas (Axis 1 and 2). The vertical axis represents the mean Dst of the species on each island; the top right coloured square is the RGB configuration used to attribute colours to the areas. **(b)** The coloured dots in the bi-dimensional representation of PCoA have been plotted on a map (equirectangular projection) showing the −100 m isobaths for north Africa and Sicily (black line), which represent a reliable reconstruction of seashores during the LGM. The background map was cropped from a map available at Natural Earth (www.naturalearthdata.com).

We used 1044 COI sequences, of which 878 have been specifically sequenced for this study and the rest obtained from GenBank. We provide all sequences as a fasta file in the Supplementary Information. Some species showed very little genetic diversification with only one or a few closely related haplotypes (e.g. *C. croceus*, *Gonepteryx cleopatra*, *Pseudophilotes baton*, *Gegenes nostrodamus*; Supplementary Note). Other highly mobile species were more variable, but the haplotypes did not show any geographic pattern (e.g. *Pieris rapae* and *Vanessa cardui*; Fig. 3). Several species were genetically differentiated between north Africa and Europe (examples provided in Fig. 3, the remaining species are available in the Supplementary Note). Lineages of some species such as *Papilio machaon*, *Lycaena phlaeas*, *Lasiommata megera* (Fig. 3), *Carcharodus alceae, Aricia* spp. and *Polyommatus celina* displayed chequered distributions, even between the two sides of the narrow Messina strait (Supplementary Note). Other species like *Hipparchia* spp., *Maniola jurtina* and *Pyronia cecilia* had distinct lineages that in some areas occur in sympatry (Fig. 3). Dst and Gst values for each species are reported in Supplementary Table S1. The Principal Coordinate Analysis (PCoA) based on the mean pairwise Gst values among areas showed that Europe and north Africa were genetically well-differentiated. Ustica, the Aeolian, the Aegadian and Maltese islands were genetically similar to Sicily-Italian Peninsula, while Lampedusa was more similar to north Africa. Pantelleria and Marettimo had a genetic signal intermediate between the two continents (Fig. 4a,b, Supplementary Note). When the mean genetic diversification among populations, measured as Dst, for the species occupying each area was plotted on the z-axis, islands with intermediate geographic positions were shown to harbour species with low population genetic differentiation (Fig. 4a).

### Correlations between species occurrence and genetic variation

**Figure 5:**
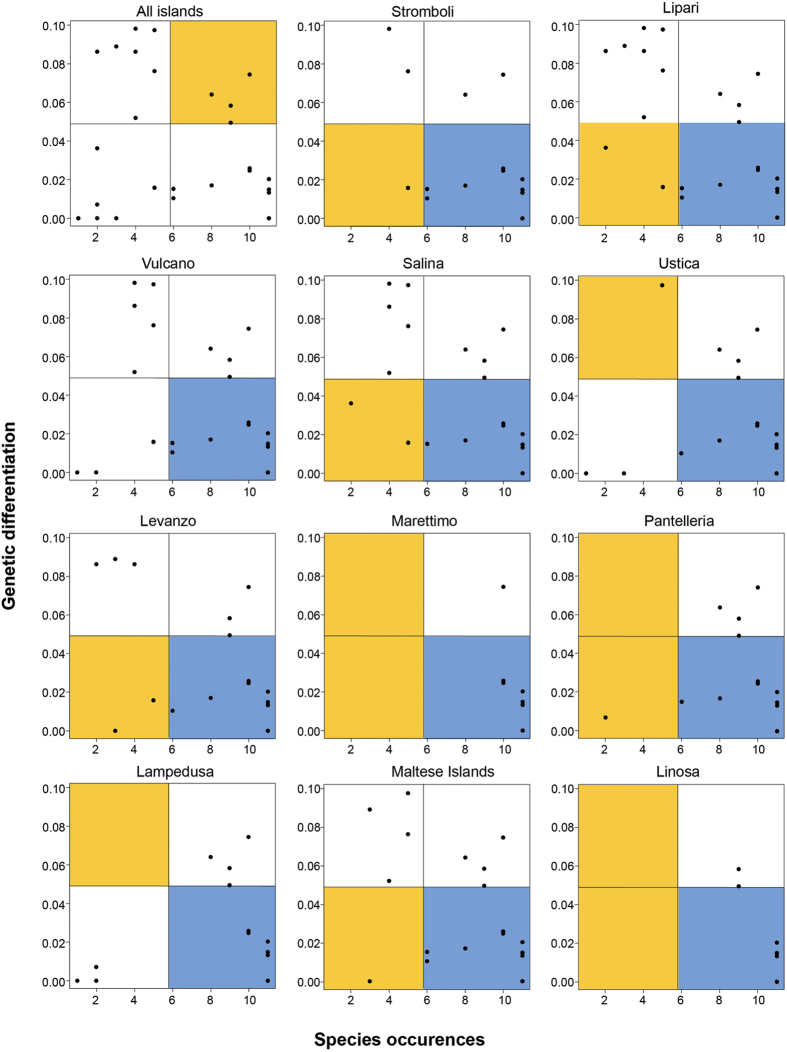
Correlations between species occurrences (x-axis) and population genetic differentiation, Dst (y-axis) for the species occurring on the studied islands. The colours indicate if a certain square is significantly filled (blue), significantly empty (orange) or shows no significant difference compared to values obtained by chance (white). All islands are significantly enriched in widespread species with low genetic variation, and most are impoverished in rare ones.

As expected, population genetic differentiation of species (measured with Dst) was significantly anti-correlated with dispersal tendency (Spearman rho = −0.409, P = 0.028, Fig. 3c). However, Dst was not significantly anti-correlated with species occurrence on islands (Spearman rho = −0.051, P = 0.791). The graph representing occurrence on islands (x-axis) and population genetic differentiation (Dst, y-axis) showed a non-random distribution of the species occurring in circum-Sicilian islands: the sector of widespread and genetically diversified species was significantly empty (Fig. 5a). When communities were examined individually, the faunas of Stromboli, Lipari, Salina, Marettimo, and Linosa showed a significant negative correlation between Dst and frequency (Supplementary Table S5). Moreover, the widespread and genetically undiversified species were always more common than expected in a random distribution. On all islands except for Lampedusa, Ustica and Vulcano there was a deficit of rare, genetically homogeneous species (Supplemetary Table S5 and Fig. 5). The Aeolian Islands, together with Levanzo and the Maltese Islands did not show a significant deficit of rare and genetically diverse species.

## Discussion

### Differentiation of populations at an intercontinental crossroad

The integration of faunistic and genetic differentiation information ultimately allowed the recognition of the main processes shaping island communities in a key biogeographic region. Similar comprehensive assessments of island communities are scarce, especially due to the difficulty of acquiring high quality data at both faunistic and genetic levels for large groups of organisms^8^,^22^,^26^,^27^. The evaluation of the contribution of singular factors in characterizing island diversity facilitated the identification of species that deserve conservation consideration on individual islands. The composition of butterfly communities and their genetic structure were shaped by neutral (frequency at source) and non-neutral (differences in dispersal tendency among species, interactions generating chequered patterns) determinants. In particular, frequency at source had a strong contribution in determining the occurrence of species on islands, as expected according to a neutral hypothesis, but species dispersal tendency also played an important role and many sedentary species were rare on islands despite their ubiquitous presence on mainland and Sicily (*M. jurtina, C. pamphilus, P. aegeria, P. cecilia* and *Aricia spp.*

One of the main findings of this study, stemming from the community and genetic data, is that even in a relatively restricted area, island populations have very different histories and are subjected to different re-colonization probabilities following local extinctions. In contrast to very isolated oceanic islands, the Western Mediterranean islands maintained relatively strong biotic relationships with the neighbouring mainland, especially for organisms with high dispersal ability, such as butterflies^24^,^28^. Accordingly, our analyses of faunistic diversity and population genetic differentiation supported that contemporary island-source dynamics, filtered by the main ecological characteristics of islands (predominantly area and isolation), are the main determinants shaping butterfly communities in the circum-Sicilian islands. Accordingly, a direct comparison between dispersal tendency and genetic variation resulted in a significant negative correlation (Fig. 2c), and about half of the species in these communities are taxa with high dispersal ability (*P. rapae, P. brassicae, C. croceus, Leptotes pirithous, Lampides boeticus, Vanessa atalanta* and *V. cardui*)^29^ and/or with low genetic diversification (Fig. 5). Island populations of these species likely include both local residents and recent immigrants^30^. Besides these widespread species, island communities include rare species (i.e. species that occur on only few islands), most of which showed considerable population differentiation over the study area. Out of 32 species, 10 occur only on one or two islands and 18 on less than half of them. Although their overall number is similar to that of the widespread species, they represent a relatively small part of each island’s diversity (on average 27.8%). These rare species did not show a nested pattern and generated inter-island faunistic dissimilarity mostly because of turnover.

The non-nested pattern of rare species revealed that basic rules of ecological filtering and colonization-extinction dynamics were not sufficient to explain occupancy patterns and that a larger array of determinants is involved, including: i) dispersal from local source populations (e.g. *P. baton* to Aeolian Islands, widespread north African species to Lampedusa), ii) interaction with sister species and lineages (as revealed by mutual exclusion, see below), iii) human impact and iv) Pleistocene connections. We confirmed the existence of marked differences in terms of butterfly diversity between north Africa and southern Europe^24^,^25^,^31^,^32^, and individual islands showed different degrees of similarity to the two sources, evidently based on their relative distance and paleogeographic connections. The richness analysis attributed to Malta, Levanzo and Lampedusa a set of species that supposedly colonized them during the LGM, likely through the land-bridges that connected these islands to mainland at that moment. This hypothesis is further supported by the finding that all the rare species with regional genetic diversification occurring on these islands are genetically very similar to the populations inhabiting the areas to which these islands were connected during the LGM.

Another important finding is the pervasive occurrence of chequered distributions^31^,^32^. This is evident not only for certain pairs of cryptic species (*Pontia edusa-P. daplidice* and *P. icarus-P. celina*), but also at the intraspecific level. Many species displaying over 1% intraspecific genetic divergence (*C. alceae, P. machaon, L. phlaeas, Aricia* spp.*, L. megera, P. aegeria, C. pamphilus*) show chequered distributions not only across the wide channel separating Sicily from north Africa, but also across the narrow (3 km) Messina strait between Sicily and southern Italy. Chequered distributions challenge the hypothesis of neutrality for species and lineages, but the relative importance of the potential mechanisms involved is still unknown. A combination of factors, such as reproductive interference, reduced dispersal, density-dependent phenomena and differences in climatic niches is probably at the basis of the observed patterns^32^.

### Island communities and the need for specifically tailored conservation measures

All the islands investigated host a significantly high fraction of dispersive species having widespread distributions in the study area and displaying low levels of genetic differentiation. Their long-term presence on islands is expected because any local extinction event would soon be followed by recolonization involving a population genetically similar to the original one. This is obvious for those species that reach Europe every spring and are unable to overwinter there^30^ [for *V. cardui*], but also applies to many others with permanent populations on islands and with high dispersal capacities (e.g. *P. rapae, P. brassicae, C. croceus, L. pirithous, L. boeticus, V. atalanta*). Any actions aiming to preserve the local island populations of these widespread species should be cautiously considered since it could represent a waste of economic resources in detriment of other, more important, conservation endeavours. On the contrary, rare species should be carefully monitored and conservation efforts immediately directed towards declining populations^33^. Their non-nested distribution patterns, with a prevalence of unpredictable species replacements between islands, and their regionally structured genetic diversity, are strong indications that these island populations have unique histories^1^,^6^,^12^. Moreover, the rare species generated the faunistic identity and the particular genetic structure of each island community, thus providing an unbalanced contribution to diversity^31^.

*Hipparchia leighebi* is a taxon endemic to the Aeolian islands with still debated status. Our results show that it represents at least a slightly diverged (0.6%) COI lineage endemic to this group of islands, where no other lineage of *Hipparchia* was detected. A COI haplotype of *M. jurtina* was the only one detected on Vulcano and the most common on Lipari, where it coexists with typical Sicilian haplotypes, and was not found elsewhere^34^. Similarly, Lampedusa hosts a substantially diverged lineage (0.8%) of the north African clade of *P. machaon* (Fig. 3, Supplementary Note). If population declines are detected for these entities, they should be considered as priority taxa for conservation in the islands where they occur. Many single individuals with unique haplotypes on islands have also been found, but their uniqueness probably represents the effect of random sampling in species with high genetic variation. This was the case for a single specimen of *L. boeticus* collected on Gozo island, having a highly diverged COI sequence close to a genetic lineage previously detected in Madagascar (Supplementary Note).

Priority for conservation should also be given to populations that apparently represent glacial relicts and that, in the current ecological settings, would unlikely recolonize a specific island. In fact, these species are the most threatened on the studied islands (Supplementary Note), possibly because they lack metapopulation dynamics that maintain population numbers (rescue effect) and are subjected to inbreeding depression. The most striking example is that of the Maltese islands, where *L. phlaeas* and *A. agestis* are believed to be extinct, while *M. jurtina, C. pamphilus* and *P. aegeria* are rapidly declining due to human impact^35^, [Paul Sammut personal observations], therefore these populations require immediate conservation actions to preserve the uniqueness of the Maltese butterfly fauna.

Levanzo and Lampedusa also had connections with Sicily and Tunisia, respectively, during the LGM. Due to the proximity to Sicily, most butterflies on Levanzo are probably part of a metapopulation and no positive richness residuals were found for this island in the GLM including contemporary determinants of richness (Fig. 1a). An exception could be *C. pamphilus*, which was represented on Levanzo by three slightly differentiated (single mutation) endemic haplotypes. On Lampedusa, all the rare species are highly dispersive and widely distributed in north Africa but not in Europe, probably because of climatic restrictions or species interactions leading to mutual exclusion^31^,^32^. However, the substantially divergent haplotype of *P. machaon* only found on this island should be considered as a priority for local conservation actions.

Chequered distributions should also be taken into consideration for conservation decisions. The possibility that density-dependent and founder-takes-all mechanisms are at the basis of the maintenance of these patterns^36^,^37^ suggests that the populations existing in an area/island represent the main barrier to the colonization by other lineages, which would change the original genetic structure, probably established after a series of unrepeatable historical events^23^. For example, the fauna of Pantelleria is mostly composed of widespread and undiversified species, but three of them (*P. celina, L. phlaeas, L. megera*) have different lineages in Sicily and Tunisia. Interestingly, all three species are represented on Pantelleria by typical north African populations, although the island was closer to Sicily during the glaciations. The similarity to Tunisia for these three taxa is the most important characteristic of this island’s butterfly fauna, but its cause is unknown. The extinction of these populations may result in recolonization from Sicily, thus erasing a key biogeographic signal on Pantelleria.

Our approach provides an example of how a series of comprehensive analyses on a wide area and large taxonomic group can test rarely assessed biogeographic principles like the links of genetic structure with dispersal tendency and frequency on islands, or the relative effects of contemporary and historical determinants on island populations. The challenge of integrating community ecology and phylogeographic approaches can also provide the baseline information for developing conservation strategies that maximize biodiversity at both the species and intraspecific genetic levels.

## Methods

### Study area and data collection

We analysed the butterfly faunas of 11 circum-Sicilian islands (Lampedusa, Levanzo, Linosa, Lipari, Maltese islands, Marettimo, Pantelleria, Salina, Stromboli, Ustica, Vulcano), Sicily itself and nearby mainland locations in southern Italy (Calabria), Tunisia and Algeria. Presence data were gathered from several post 1980 literature sources and from field surveys carried out by the authors between 1999 and 2015. Specimens used for genetic analyses were collected outside protected areas and were deposited at the Institute of Evolutionary Biology (CSIC-UPF), Barcelona, Spain.

### Determinants for island species richness

To evaluate the influence of different factors on species richness for the studied islands, we used Generalized Linear Models (GLM) with Akaike Information Criterion (AIC). We also assessed the relative importance of potentially correlated variables using hierarchical partitioning of variance, employing the *lmg* metric implemented in the “relaimpo” R Package. To place the richness of the study islands in a broader framework, we used presence data on the butterfly fauna from all the western Mediterranean islands^24^ (excluding Sicily, Sardinia and Corsica because their large size would provide an unbalanced contribution to the analysis). Islands were included in the study if at least four out of five migrant species (*P. brassicae*, *P. rapae*, *C. croceus*, *V. atalanta* and *V. cardui*) were recorded there. These species are conspicuous and widespread throughout Europe and north Africa, and their records establish a minimal surveying standard^24^.

In the GLM, species richness was modelled against the following biotic, geographic and climatic predictors: i) mean annual temperatures (MT), ii) annual precipitation (AP), iii) island area (IA), iv) maximum elevation of island (EL), v) isolation (IS), vi) source richness (SR) and vii) the occurrence of a Pleistocene connection (PC, factor variable). MT and AP were obtained by computing the mean values of the cells corresponding to islands in Bio1 and Bio12 layers from Bioclim (http://www.worldclim.org). For each island, the faunistic source was identified as the nearest 50×50 km area, either mainland (southern Europe or north Africa), large island (Sicily, Sardinia, Corsica), or an island at least ten times larger than the target one. We calculated SR as the number of species reported from the source area and IS as the minimum distance between the target island and its faunistic source. To linearize the relationships, we log transformed richness, area, isolation and source richness. We computed a first GLM using only contemporary variables and a second GLM in which we included the PC. We compared the AIC, explanatory power and residuals of the selected variables between the two models.

### Community structure

We estimated the degree of nestedness of butterfly communities in circum-Sicilian islands by applying the widely accepted NODF metric^9^. A recent study has shown that the detected degree of nestedness depends heavily on the selected null models due to their different tendency to preserve the features of the original matrix^12^. To assess the significance of the observed NODF, we used the NeD program^38^ and computed the z-values using 999 null matrices, built by applying a series of different models (equiprobable rows and columns, EE; proportional rows and columns total, CE; and fixed rows and columns, FF)^12^.

Prior studies have divided species into “core” and “satellite” species^39^ in which “core” species are those occurring on more than half of the islands, and “satellite” those present on fewer islands. We used the same approach and divided the 27 species occurring on the 11 studied islands into “widespread” (those occurring on six or more of the islands) and “rare” (those found on no more than five islands). We also separately analysed the degree of nestedness of the two subsets.

The overall faunistic dissimilarities among areas can be partitioned between a component generated by nestedness and one determined by species replacement (turnover)^40^. We calculated the relative contribution of nestedness and turnover as the mean value of the ratio between the faunistic dissimilarities obtained by nestedness and Sørensen indexes (see Supplementary Information and Supplementary Data)^41^. This ratio has been computed for all species, as well as for the rare and widespread species groups, separately. We tested for the effect of species dispersal tendency and their frequency at source on their occupancy on islands. Measurements of the dispersal tendency in butterflies are complex and, in previous studies, have been mostly based on the agreement among subjective evaluations made by experts, and less commonly by using objective species traits. Here we combined the indexes provided by four studies^42^,^43^,^44^,^45^ by standardizing their range between 0 (low dispersal) and 1 (high dispersal) and computed, for each species, an average dispersal tendency based on the available measurements. To estimate the frequency at source we counted the number of cells of 0.1 × 0.1 degrees between 34° and 40° latitude and 7° and 18° longitude, where each of the 32 investigated species has been reported. As standardized sources we used “CKmap2000”, an online checklist of the Italian butterfly fauna^46^, and the database of the Butterfly Diversity and Evolution Lab at The Institute of Evolutionary Biology (CSIC-UPF), Spain. The frequency of each species on islands was regressed against dispersal tendency and frequency at source, and the relative importance of these factors was tested by hierarchical partitioning of variance.

### Genetic analyses and identification of study units

Using standard sequencing procedures (see Supplementary Information) we obtained cytochrome *c* oxidase subunit I (COI) sequences for 1044 specimens (Supplementary Table S2) from the study islands and from five surrounding areas: southern Italy (Calabria), eastern Sicily (>14° longitude), western Sicily (<14° longitude), Tunisia and Algeria. We considered only the islands for which we had available more than 80% of the reported fauna. This led to the exclusion of Linosa and left ten islands and a total of 15 areas to be analysed.

The butterfly species currently recognized by taxonomists show different levels of intraspecific genetic divergence and they can have an unbalanced contribution to the overall biogeographic pattern^31^,^47^. To reduce this bias, we identified as units the groups of individuals having COI p-distances less than 3%, a measure that, in Lepidoptera, was reported to separate more than 90% of recognized sister species^48^. By applying this threshold we identified 29 units (termed species) closely matching the taxonomy proposed by Fauna Europaea (www.faunaeur.org). The list of species is provided in Supplementary Table S1.

### Overall population differentiation pattern

To examine patterns of genetic differentiation for each species in the study area we constructed haplotype networks with the TCS Network method in PopART^49^ (http://popart.otago.ac.nz). We calculated the genetic uncorrected p-distances among all sequenced specimens for each species, as well as two measures for population differentiation: Dst and Gst^50^:


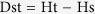


where Ht represents the average intraspecific p-distances for all specimens of a given species, and Hs is the average of the intra-population p-distances. Thus, Dst represents average genetic differentiation among populations in p-distance units. The second measure (Gst) is a standardized index defined as:


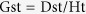


representing which fraction of the total genetic differentiation is encompassed by differentiation among populations^50^. This index ranges from negative values to 1 (complete differentiation). Negative values (intra-area differentiation higher than inter-area differentiation) can have different subtle meanings, but they are mostly generated as a bias due to relatively small sample sizes; usually they are set to zero^51^ and we used this solution for two cases in our study. We also calculated pairwise Gst among pairs of populations using the following formula:


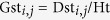


representing which fraction of the overall genetic diversity (Ht) is expressed by inter-population diversification (Dst_i,j_) between a given pair of areas (i and j).

By using the Gst pairwise matrices for each species, we calculated the mean of the available values of the corresponding cells to produce the final mean Gst matrix, representing the degree of genetic differentiation among areas based on all species. A PCoA was applied to this matrix to obtain the overall genetic pattern among areas. Subsequently, we aligned this configuration with the geographic location of the areas by using the “procrustes” analysis from the “vegan” R package (Supplementary Data). To visualize the pattern of similarity among islands over geographic space, we projected the final configuration of average Gst values among areas in the RGB space^24^,^52^ using the R package “recluster”. The colour resemblance of the resulting dots is directly proportional to the genetic differentiation among the communities. These dots were then plotted on a map, where we outlined the −100 m depth contour as a reliable reconstruction of land during the LGM^20^.

Islands located between two genetically contrasting sources can show intermediate communities if: i) islands host species characterized by genetic variation and individuals belong to different sources, or ii) islands host only a reduced fraction of species characterized by genetic differentiation, and therefore look equally similar to both sources. To visualize the pattern of genetic similarity and to compare the degree of genetic differentiation among areas we computed for each area the mean Dst as the mean of all the Dst values for the species available for each area. We then plotted the relationship between the PCoA configuration and the mean Dst of each area in 3D. If the first hypothesis is met, we expect that intermediate islands will have similar mean Dst to other islands, while if the second is true we expect that intermediate islands will have lower mean Dst values.

### Analysis of genetic differentiation among populations

We tested for correlation between Dst and both dispersal tendency and island occupancy (number of studied islands occupied by each species) using the non-parametric Spearman correlation rank test. We also square root transformed the Dst values to improve normality and divided the bivariate relationship between occupancy and Dst into four quadrants, and considered the species either as ‘widespread’ or ‘rare’ and the absolute genetic variation as low (<half) or high (>half) of the maximum square root transformed Dst. The number of species falling into each quadrant was scored. Subsequently, 999 null matrices were constructed by attributing to each species random values of occupancy and Dst. For each square, we assessed the frequency for which the observed number of species was lower than in random configurations. Values <0.050 were considered as significantly empty quadrants, and values >0.950 as significantly full quadrants.

## Additional Information

**How to cite this article**: Vodă, R. *et al*. Historical and contemporary factors generate unique butterfly communities on islands. *Sci. Rep.* **6**, 28828; doi: 10.1038/srep28828 (2016).

## Supplementary Material

Supplementary Information

Supplementary Note

Supplementary Dataset 1

## Figures and Tables

**Table 1:** Results for the AIC-based stepwise Generalized Linear Model predicting species richness on islands based on: IA = island area; EL = maximum elevation of island; IS = island isolation from the nearest source; SR = butterfly richness of the nearest source; PC = occurrence of Pleistocene connection.

	Est.	S.E.	t	P	lmg (%)
MT					
AP					
IA	0.069	0.039	1.775	0.088	14.0
EL	0.188	0.069	2.722	0.011	12.6
IS	−0.111	0.048	−2.299	0.030	14.9
SR	0.588	0.127	4.637	<0.001	20.1
PC	0.159	0.070	2.268	0.031	11.6
					73.2%

Mean annual temperature and annual precipitation did not enter the model. Abbreviations: Est = estimated parameter; SE = Standard Error; t = t value; P = P value; lmg = percentage of explained variance attributed by hierarchical partition of variance.

**Table 2:** The residuals scored in the Generalized Linear Models for each island having had contact with mainland areas during the LGM, obtained either by removing (Res. 1) or including (Res. 2) the Pleistocene connection as a categorical predictor.

Island	Res. 1	Res. 2	Diff.
Maltese	3.5	0.8	2.7
Levanzo	−0.3	−4.3	4.0
Lampedusa	4.5	1.6	2.9

The difference between the two values can serve as an estimate for the number of species that colonized the islands during the LGM and survived until present.

**Table 3:** Results for the ratio between the nestedness beta diversity index (βnest) and the unpartitioned Sørensen index (βsor) and observed NODF for the nestedness analyses, using all species, only rare species, and only widespread species.

	βnest/βsor	NODF	Null Model	Mean NODF	Z
All species	0.603	75.613	EE	51.673	9.664***
			CE	60.560	4.739***
			FF	77.549	−2.698**
Rare species	0.271	39.491	EE	32.451	1.649*
			CE	37.739	0.275
			FF	43.557	−2.016*
Widespread species	0.814	82.013	EE	71.095	2.378*
			CE	74.553	1.989*
			FF	82.206	−1.196

Mean NODF and Z values are provided for different series of 999 matrices generated by different null models (EE, equiprobable rows and columns; CE, proportional rows and columns total; and FF, fixed rows and columns). Asterisks represent associated P values.
