# Supplementary material for: Historical and contemporary factors generate unique butterfly communities on islands
Source: Sci Rep. 2016 Jun 29;6:28828. doi: 10.1038/srep28828 (PMC4926222; doi:10.1038/srep28828)
Supplement: Supplementary Information [file srep28828-s1.pdf]

Supplementary Information

Historical and contemporary factors generate unique butterfly communities on islands

Raluca Vodă[a,b‡], Leonardo Dapporto[a,c‡], Vlad Dincă[a,d], Tim G. Shreeve[e], Mourad Khaldi[f], Ghania Barech[g], Khellaf Rebbas[g], Paul Sammut[h], Stefano Scalercio[i], Paul D.N. Hebert[d], Roger Vila[a*]

[a]Institut de Biologia Evolutiva (CSIC-Universitat Pompeu Fabra), Passeig Marítim de la Barceloneta 37, 08003, Barcelona, Spain

[b]Departament de Genètica i Microbiologia, Universitat Autònoma de Barcelona, 08193, Bellaterra, Spain

[c]Department of Biology, University of Florence, 50019 Florence, Italy

[d]Biodiversity Institute of Ontario, University of Guelph, Guelph, N1G 2W1, Ontario, Canada

[e]Department of Biological and Medical Sciences, Oxford Brookes University, Headington, Oxford, OX3 0BP, UK

[f]Département d'Agronomie, Université Mohamed Boudiaf de M'sila, 28000 M'sila, Algeria

[g]Département des sciences de la nature et de la vie, Université Mohamed Boudiaf de M'sila, 28000 M'sila, Algeria

[h]137, "Fawkner/2" Dingli Road, Rabat RBT 9023, Malta

[i]Consiglio per la ricerca in agricoltura e l'analisi dell'economia agraria, Unità di Ricerca per la Selvicoltura in Ambiente Mediterraneo, c.da Li Rocchi, I-87036 Rende (CS), Italy

*Equal contribution

Supplementary Methods

COI sequencing

Total genomic DNA was extracted using Chelex 100 resin, 100–200 mesh, sodium form (Biorad), under the following protocol: one leg was removed and introduced into 100 µL of Chelex 10% and 5 µL of Proteinase K (20 mg⁄mL) were added. The samples were incubated overnight at 55°C and were subsequently incubated at 100°C for 15 minutes. Samples were then centrifuged for 10 seconds at 3.000 rpm. A 658-bp fragment at the 5' end of the mitochondrial gene (COI) was amplified by polymerase chain reaction using the primers LepF1 (5′-ATTCAACCAATCATAAAGATATTGG-3′) and LepR1 (5′-TAAACTTCTGGATGTCCAAAAAATCA-3′) (Hebert et al., 2004). Double-stranded DNA was amplified in 25-µL volume reactions containing: 14.4 µL autoclaved Milli-Q water, 5 µL 5x buffer, 2 µL 25 mM MgCl2, 0.5 µL 10 mM dNTPs, 0.5 µL of each primer (10 µM), 0.1 µLTaq DNA Polymerase (Promega, 5U/µL) and 2 µL of extracted DNA. The typical thermal cycling profile was: first denaturation at 92 ºC for 60 s, followed by five cycles of 92 ºC for 15 s, 49 ºC for 45 s and 62 ºC for 150 s, and then by 35 cycles of 92 ºC for 15 s, 52 ºC for 45 s and 62 ºC for 150 s and a final extension at 62 ºC for 420 s. PCR products were purified and sequenced by Macrogen Inc. Sequences were edited and aligned using GENEIOUS PRO 6.0.5 created by Biomatters (http://www.geneious.com/). 301 of the 333 novel sequences generated by this study have been obtained at the Biodiversity Institute of Ontario, Canada. In this case a glass fibre protocol (Ivanova et al., 2006) was employed to extract DNA and polymerase chain reactions (PCR) and DNA sequencing were carried out following standard DNA barcoding procedures for Lepidoptera (deWaard et al., 2008).

Species analysed in this study

We identified as units the groups of individuals showing COI p-distances lower than 3%. By applying this threshold we identified 29 units (termed species) closely matching the taxonomy proposed by Fauna Europaea (www.faunaeur.org).

Supplementary Table S1 The units considered as species and the corresponding taxonomic identification according to Fauna Europaea. Population differentaition measured as Gst and Dst is also reported. The specific status of *Coenonympha lyllus* is still debated and the low divergence we found (about 1%) suggests that C. *lyllus* should be considered as a subspecies of C. *pamphilus*.

Family	Genetic Units	Species in Fauna Europea	No. of species analysed for COI	Gst	Dst
**Papilionidae**					
	Papilio machaon	*Papilio machaon*	38	0.549	0.411
**Pieridae**					
	Pieris rapae	*Pieris rapae*	72	0.052	0.018
	Pieris brassicae	*Pieris brassicae*	42	0.299	0.060
	Pieris mannii	*Pieris mannii*	18	0.128	0.025
	Pontia edusa	*Pontia edusa*	29	0.052	0.011
	Pontia daplidice	*Pontia daplidice*	20	0.011	0.005
	Euchloe ausonia	*Euchloe ausonia*	15	NA	0.000
	Colias croceus	*Colias croceus*	50	NA	0.000
	Gonepteryx cleopatra	*Gonepteryx cleopatra*	21	NA	0.000
**Lycaenidae**					
	Lycaena phlaeas	*Lycaena phlaeas*	45	0.920	0.245
	Callophrys rubi	*Callophrys rubi*	-		
	Zizeeria karsandra	*Zizeeria karsandra*	-		
	Celastrina argiolus	*Celastrina argiolus*	37	0.279	0.023
	Lampides boeticus	*Lampides boeticus*	41	0.207	0.041
	Leptotes pirithous	*Leptotes pirithous*	32	0.659	0.067
	Pseudophilotes baton	*Pseudophilotes baton*	11	NA	0.000
	Aricia agestis	*Aricia agestis*<br>*Aricia cramera*	58	0.921	0.948
	Polyommatus celina	*Polyommatus celina*	90	0.557	0.340
**Nymphalidae**					
	Danaus chryshippus	*Danaus chryshippus*	9	0.000	0.000
	Vanessa atalanta	*Vanessa atalanta*	28	0.261	0.022
	Vanessa cardui	*Vanessa cardui*	31	0.000	0.000
	Polygonia egea	*Polygonia egea*	-		
	Charaxes jasius	*Charaxes jasius*	8	1.000	0.130
	Coenonympha pamphilus	*Coenonympha pamphilus*<br>*Coenonympha lyllus**	39	0.861	0.791
	Pyronia cecilia	*Pyronia cecilia*	45	0.557	0.742
	Maniola jurtina	*Maniola jurtina*	54	0.534	0.270
	Lasiommata megera	*Lasiommata megera*	77	0.901	0.556
	Pararge aegeria	*Pararge aegeria*	50	0.955	0.579
	Hipparchia semele	*Hipparchia semele*<br>*Hipparchia leighebi*<br>*Hipparchia algirica*<br>*Hipparchia blachieri*	34	0.704	0.964
**Hesperiidae**					
	Carcharodus alceae	*Carcharodus alceae*	18	0.830	0.743
	Gegenes nostrodamus	*Gegenes nostrodamus*	11	0.000	0.000
	Gegenes pumilio	*Gegenes pumilio*	21	0.405	0.029

Supplementary Table S2 List of specimens used in this study and their corresponding localities

Sample ID	Genbank	Family	Genus	Species	Date collected	Area	Latitude	Longitude
RVcoll11D385	KM459051	Lycaenidae	*Aricia*	*agestis*	2011-04-13	Sicily	37,158	15,049
RVcoll11D368	KM459050	Lycaenidae	*Aricia*	*agestis*	2011-04-13	Sicily	37,197	15,118
RVcoll12M384	-	Lycaenidae	*Aricia*	*agestis*	2012-05-10	Sicily	37,493	13,684
RVcoll11D296	KM459049	Lycaenidae	*Aricia*	*agestis*	2011-04-12	Sicily	37,626	15,070
RVcollLD2988	KM459102	Lycaenidae	*Aricia*	*agestis*	2010-06-30	Sicily	37,699	13,144
RVcoll12Q581	KM459091	Lycaenidae	*Aricia*	*agestis*	2012-08-01	Sicily	37,800	15,040
RVcoll11I054	KM459067	Lycaenidae	*Aricia*	*agestis*	2011-06-18	Sicily	37,810	15,070
RVcoll11D214	KM459048	Lycaenidae	*Aricia*	*agestis*	2011-04-11	Sicily	37,838	13,429
RVcollLD2968	KM517846	Lycaenidae	*Aricia*	*agestis*	2010-06-30	Sicily	37,845	13,438
RVcollLD2969	KM517836	Lycaenidae	*Aricia*	*agestis*	2010-06-30	Sicily	37,845	13,438
RVcollLD0621	JX678137	Lycaenidae	*Aricia*	*agestis*	2008-05-26	Sicily	37,868	13,384
RVcollLD0622	JX678138	Lycaenidae	*Aricia*	*agestis*	2008-05-26	Sicily	37,868	13,384
RVcoll11H946	KM459066	Lycaenidae	*Aricia*	*agestis*	2011-06-16	Sicily	37,880	14,030
RVcoll11H951	KM517835	Lycaenidae	*Aricia*	*agestis*	2011-06-16	Sicily	37,880	14,030
RVcoll11H340	KM459060	Lycaenidae	*Aricia*	*agestis*	2011-07-21	Sicily	37,900	14,010
RVcoll11H342	KM517847	Lycaenidae	*Aricia*	*agestis*	2011-07-21	Sicily	37,900	14,010
RVcoll11J702	KM517838	Lycaenidae	*Aricia*	*agestis*	2011-07-21	Sicily	37,900	14,010
RVcoll11J703	KM459079	Lycaenidae	*Aricia*	*agestis*	2011-07-21	Sicily	37,900	14,010
RVcoll11J704	KM459080	Lycaenidae	*Aricia*	*agestis*	2011-07-21	Sicily	37,900	14,010
RVcoll11I007	KM517839	Lycaenidae	*Aricia*	*agestis*	2011-06-17	Sicily	37,900	14,020
RVcoll12Q584	KM459093	Lycaenidae	*Aricia*	*agestis*	2012-08-02	Sicily	37,980	14,870
RVcoll12Q585	KM459094	Lycaenidae	*Aricia*	*agestis*	2012-08-02	Sicily	37,980	14,870
RVcoll12Q580	KM459090	Lycaenidae	*Aricia*	*agestis*	2012-07-31	Italy	38,067	15,817
RVcoll10C504	KM459044	Lycaenidae	*Aricia*	*agestis*	2010-06-26	Italy	38,120	15,670
RVcoll11D035	KM459047	Lycaenidae	*Aricia*	*agestis*	2011-04-07	Sicily	38,139	13,126
RVcoll12Q583	KM459092	Lycaenidae	*Aricia*	*agestis*	2012-08-01	Sicily	38,160	15,470
RVcoll09T582	KM459035	Lycaenidae	*Aricia*	*agestis*	2009-07-03	Italy	38,192	15,993
RVcollLD2840	KM459101	Lycaenidae	*Aricia*	*agestis*	2010-06-27	Sicily	38,208	15,499
RVcoll12Q579	KM459089	Lycaenidae	*Aricia*	*agestis*	2012-07-31	Italy	38,250	15,950
RVcoll11H859	KM459061	Lycaenidae	*Aricia*	*agestis*	2011-06-14	Vulcano	38,380	14,980
RVcoll11H860	KM459062	Lycaenidae	*Aricia*	*agestis*	2011-06-14	Vulcano	38,380	14,980
RVcoll11H861	KM459063	Lycaenidae	*Aricia*	*agestis*	2011-06-14	Vulcano	38,380	14,980
RVcoll11H862	KM459064	Lycaenidae	*Aricia*	*agestis*	2011-06-14	Vulcano	38,380	14,980
RVcoll11H863	KM459065	Lycaenidae	*Aricia*	*agestis*	2011-06-14	Vulcano	38,380	14,980
RVcoll10C676	-	Lycaenidae	*Aricia*	*agestis*	2010-06-30	Lipari	38,482	14,931
RVcoll09T554	KM517842	Lycaenidae	*Aricia*	*agestis*	2009-07-01	Lipari	38,490	14,930
RVcoll12M512	-	Lycaenidae	*Aricia*	*agestis*	2012-05-12	Italy	38,601	16,184
LEPSS00045	-	Lycaenidae	*Aricia*	*agestis*	2014-05-23	Italy	39,315	16,528
RVcoll07E047	JX678119	Lycaenidae	*Aricia*	*agestis*	2007-07-11	Italy	39,336	16,358
LEPSS00072	-	Lycaenidae	*Aricia*	*agestis*	2014-07-08	Italy	39,354	16,544
RVcoll15A823	-	Lycaenidae	*Aricia*	*agestis*	2015-06-06	Italy	39,388	16,602
LEPSS00044	-	Lycaenidae	*Aricia*	*agestis*	2014-05-20	Italy	39,443	16,604
LEPSS00043	-	Lycaenidae	*Aricia*	*agestis*	2014-05-20	Italy	39,560	16,751
RVcoll11I276	KM459068	Lycaenidae	*Aricia*	*agestis*	2011-06-20	Italy	39,860	16,070
RVcoll14L020	-	Lycaenidae	*Aricia*	*cramera*	2007-05-22	Algeria	35,100	1,150
RVcoll14L026	-	Lycaenidae	*Aricia*	*cramera*	2007-05-23	Algeria	35,280	0,920
RVcoll12N581	KM459148	Lycaenidae	*Aricia*	*cramera*	2012-05-18	Algeria	35,326	4,297
RVcoll08J935	KM517845	Lycaenidae	*Aricia*	*cramera*	2008-07-09	Tunisia	35,745	8,384
RVcoll12N247	-	Lycaenidae	*Aricia*	*cramera*	2012-05-28	Tunisia	36,294	9,811
RVcoll12N994	-	Lycaenidae	*Aricia*	*cramera*	2012-07-12	Algeria	36,479	3,998
RVcoll12N937	-	Lycaenidae	*Aricia*	*cramera*	2012-06-16	Algeria	36,630	4,598
RVcoll12N207	KM459147	Lycaenidae	*Aricia*	*cramera*	2012-05-28	Tunisia	36,651	10,161
RVcoll08J944	KM459112	Lycaenidae	*Aricia*	*cramera*	2008-07-10	Tunisia	36,782	8,751
RVcoll08J946	KM459113	Lycaenidae	*Aricia*	*cramera*	2008-07-10	Tunisia	36,782	8,751
RVcoll13T385	-	Lycaenidae	*Aricia*	*cramera*	2013-07-04	Tunisia	37,164	10,110
RVcollLD0078	JX678037	Lycaenidae	*Aricia*	*cramera*	2008-04-26	Tunisia	37,177	9,725
RVcollLD0081	JX678038	Lycaenidae	*Aricia*	*cramera*	2008-04-26	Tunisia	37,177	9,725
RVcoll13T396	-	Lycaenidae	*Aricia*	*cramera*	2013-07-05	Tunisia	37,313	9,856
RVcoll11D380	-	Hesperiidae	*Carcharodus*	*alceae*	2011-04-13	Sicily	37,158	15,050
RVcoll12M380	-	Hesperiidae	*Carcharodus*	*alceae*	2012-05-10	Sicily	37,493	13,684
RVcollLD2990	-	Hesperiidae	*Carcharodus*	*alceae*	2010-06-30	Sicily	37,699	13,144
RVcoll11H970	-	Hesperiidae	*Carcharodus*	*alceae*	2011-06-16	Sicily	37,710	13,970
RVcoll14N605	-	Hesperiidae	*Carcharodus*	*alceae*	2014-09-27	Levanzo	37,987	12,342
RVcollLD2596	-	Hesperiidae	*Carcharodus*	*alceae*	2010-04-15	Sicily	37,993	14,056
RVcollLD2786	-	Hesperiidae	*Carcharodus*	*alceae*	2010-04-04	Sicily	38,005	14,351
RVcoll11H592	-	Hesperiidae	*Carcharodus*	*alceae*	2011-06-08	Sicily	38,172	13,325
RVcollLD2587	-	Hesperiidae	*Carcharodus*	*alceae*	2010-06-01	Sicily	38,177	15,474
RVcollLD2854	-	Hesperiidae	*Carcharodus*	*alceae*	2010-06-27	Sicily	38,208	15,499
RVcoll12M480	-	Hesperiidae	*Carcharodus*	*alceae*	2012-05-12	Italy	38,250	15,951
RVcoll12M481	-	Hesperiidae	*Carcharodus*	*alceae*	2012-05-12	Italy	38,250	15,951
RVcoll14A664	-	Hesperiidae	*Carcharodus*	*alceae*	2013-08-19	Italy	38,389	16,194
RVcollLD2568	-	Hesperiidae	*Carcharodus*	*alceae*	2009-06-30	Lipari	38,482	14,931
RVcoll12M515	-	Hesperiidae	*Carcharodus*	*alceae*	2012-05-12	Italy	38,601	16,184
LEPSS00008	-	Hesperiidae	*Carcharodus*	*alceae*	2014-05-23	Italy	39,315	16,528
LEPSS00009	-	Hesperiidae	*Carcharodus*	*alceae*	2014-05-27	Italy	39,323	16,475
RVcoll14A678	-	Hesperiidae	*Carcharodus*	*alceae*	2013-08-20	Italy	39,483	16,801

RVcoll10C584	-	Lycaenidae	*Celastrina*	*argiolus*	2010-06-17	Maltese	35,850	14,390
RVcoll10C585	-	Lycaenidae	*Celastrina*	*argiolus*	2010-06-17	Maltese	35,850	14,390
RVcoll10C583	-	Lycaenidae	*Celastrina*	*argiolus*	2010-06-17	Maltese	35,858	14,399
RVcollLD2733	-	Lycaenidae	*Celastrina*	*argiolus*	2010-06-17	Maltese	35,858	14,399
RVcoll11E111	-	Lycaenidae	*Celastrina*	*argiolus*	2011-05-20	Maltese	36,042	14,274
RVcoll11E112	-	Lycaenidae	*Celastrina*	*argiolus*	2011-05-20	Maltese	36,042	14,274
RVcoll12N287	-	Lycaenidae	*Celastrina*	*argiolus*	2012-05-28	Tunisia	36,116	9,656
RVcoll12N602	-	Lycaenidae	*Celastrina*	*argiolus*	2012-05-10	Algeria	36,592	4,611
RVcoll12N635	-	Lycaenidae	*Celastrina*	*argiolus*	2012-05-18	Algeria	36,594	4,608
RVcoll12N929	-	Lycaenidae	*Celastrina*	*argiolus*	2012-06-16	Algeria	36,630	4,598
RVcoll12N497	-	Lycaenidae	*Celastrina*	*argiolus*	2012-06-01	Tunisia	36,731	8,710
RVcoll11D113	-	Lycaenidae	*Celastrina*	*argiolus*	2011-04-08	Pantelleria	36,785	11,981
RVcoll11H657	-	Lycaenidae	*Celastrina*	*argiolus*	2011-06-11	Pantelleria	36,790	12,000
RVcoll11D152	-	Lycaenidae	*Celastrina*	*argiolus*	2011-04-09	Pantelleria	36,819	12,006
RVcoll12N547	-	Lycaenidae	*Celastrina*	*argiolus*	2012-06-02	Tunisia	36,907	9,176
RVcoll11D387	-	Lycaenidae	*Celastrina*	*argiolus*	2011-04-13	Sicily	37,158	15,050
RVcoll13T397	-	Lycaenidae	*Celastrina*	*argiolus*	2013-07-05	Tunisia	37,313	9,856
RVcoll12Q973	-	Lycaenidae	*Celastrina*	*argiolus*	2012-08-01	Sicily	37,800	15,040
RVcoll11D238	-	Lycaenidae	*Celastrina*	*argiolus*	2011-04-11	Sicily	37,838	13,429
RVcoll11H535	-	Lycaenidae	*Celastrina*	*argiolus*	2011-06-08	Sicily	38,093	13,260
RVcoll11D029	-	Lycaenidae	*Celastrina*	*argiolus*	2011-04-07	Sicily	38,139	13,126
RVcoll11I116	-	Lycaenidae	*Celastrina*	*argiolus*	2011-06-19	Italy	38,221	16,183
RVcoll11D436	-	Lycaenidae	*Celastrina*	*argiolus*	2011-04-14	Sicily	38,238	15,528
RVcoll14N636	-	Lycaenidae	*Celastrina*	*argiolus*	2014-09-30	Vulcano	38,380	14,970
RVcoll11H806	-	Lycaenidae	*Celastrina*	*argiolus*	2011-06-14	Vulcano	38,390	14,970
RVcoll11H807	-	Lycaenidae	*Celastrina*	*argiolus*	2011-06-14	Vulcano	38,390	14,970
RVcoll11H808	-	Lycaenidae	*Celastrina*	*argiolus*	2011-06-14	Vulcano	38,390	14,970
RVcollLD2558	-	Lycaenidae	*Celastrina*	*argiolus*	2009-06-30	Lipari	38,482	14,931
RVcoll11H870	-	Lycaenidae	*Celastrina*	*argiolus*	2011-06-15	Salina	38,560	14,830
RVcoll11H871	-	Lycaenidae	*Celastrina*	*argiolus*	2011-06-15	Salina	38,560	14,840
RVcoll12M506	-	Lycaenidae	*Celastrina*	*argiolus*	2012-05-12	Italy	38,601	16,184
RVcoll11D514	-	Lycaenidae	*Celastrina*	*argiolus*	2011-04-18	Italy	38,674	15,926
RVcoll14N725	-	Lycaenidae	*Celastrina*	*argiolus*	2014-10-03	Stromboli	38,800	15,210
RVcollLD2574	-	Lycaenidae	*Celastrina*	*argiolus*	2009-07-01	Stromboli	38,803	15,228
LEPSS00075	-	Lycaenidae	*Celastrina*	*argiolus*	2014-07-25	Italy	39,335	16,399
LEPSS00076	-	Lycaenidae	*Celastrina*	*argiolus*	2014-07-25	Italy	39,335	16,399
RVcoll14A679	-	Lycaenidae	*Celastrina*	*argiolus*	2013-08-20	Italy	39,483	16,801
RVcoll13T455	-	Nymphalidae	*Charaxes*	*jasius*	2013-07-07	Tunisia	36,726	8,705
RVcoll13T456	-	Nymphalidae	*Charaxes*	*jasius*	2013-07-07	Tunisia	36,726	8,705
RVcoll14V864	-	Nymphalidae	*Charaxes*	*jasius*	2011-07-02	Sicily	38,215	15,505
RVcollLD2557	-	Nymphalidae	*Charaxes*	*jasius*	2009-06-30	Lipari	38,482	14,931
RVcoll14N672	-	Nymphalidae	*Charaxes*	*jasius*	2014-10-01	Salina	38,570	14,830
RVcoll14N673	-	Nymphalidae	*Charaxes*	*jasius*	2014-10-01	Salina	38,570	14,830
RVcoll14V916	-	Nymphalidae	*Charaxes*	*jasius*	2015-06-05	Italy	39,496	16,746
RVcoll14V917	-	Nymphalidae	*Charaxes*	*jasius*	2015-06-05	Italy	39,496	16,746
RVcoll14L080	-	Nymphalidae	*Coenonympha*	*pamphilus*	2009-05-28	Algeria	35,190	1,130
RVcoll14L023	-	Nymphalidae	*Coenonympha*	*pamphilus*	2007-05-23	Algeria	35,280	0,920
RVcoll14L119	-	Nymphalidae	*Coenonympha*	*pamphilus*	2014-05-04	Algeria	35,860	4,750
RVcoll14E251	-	Nymphalidae	*Coenonympha*	*pamphilus*	2014-03-23	Maltese	35,961	14,368
RVcoll14E255	-	Nymphalidae	*Coenonympha*	*pamphilus*	2014-03-23	Maltese	35,961	14,368
RVcoll14E258	-	Nymphalidae	*Coenonympha*	*pamphilus*	2014-03-23	Maltese	35,961	14,368
RVcoll14E259	-	Nymphalidae	*Coenonympha*	*pamphilus*	2014-03-23	Maltese	35,961	14,368
RVcoll13T314	-	Nymphalidae	*Coenonympha*	*pamphilus*	2013-07-08	Tunisia	36,225	8,771
RVcoll12N612	-	Nymphalidae	*Coenonympha*	*pamphilus*	2012-05-12	Algeria	36,260	5,229
RVcoll12N470	-	Nymphalidae	*Coenonympha*	*pamphilus*	2012-05-31	Tunisia	36,290	8,789
RVcoll12N471	-	Nymphalidae	*Coenonympha*	*pamphilus*	2012-05-31	Tunisia	36,290	8,789
RVcoll12N607	-	Nymphalidae	*Coenonympha*	*pamphilus*	2012-05-11	Algeria	36,329	5,360
RVcoll12N628	-	Nymphalidae	*Coenonympha*	*pamphilus*	2012-05-13	Algeria	36,592	4,611
RVcoll12N880	-	Nymphalidae	*Coenonympha*	*pamphilus*	2012-05-24	Algeria	36,615	4,636
RVcoll12N478	-	Nymphalidae	*Coenonympha*	*pamphilus*	2012-06-01	Tunisia	36,731	8,710
RVcoll13T301	-	Nymphalidae	*Coenonympha*	*pamphilus*	2013-07-05	Tunisia	36,860	8,720
RVcoll11D346	-	Nymphalidae	*Coenonympha*	*pamphilus*	2011-04-13	Sicily	37,197	15,118
RVcoll12M342	-	Nymphalidae	*Coenonympha*	*pamphilus*	2012-05-07	Sicily	37,201	14,167
RVcoll12M381	-	Nymphalidae	*Coenonympha*	*pamphilus*	2012-05-10	Sicily	37,493	13,684
RVcoll11Y107	-	Nymphalidae	*Coenonympha*	*pamphilus*	2011-06-29	Sicily	37,620	13,050
RVcoll11D316	-	Nymphalidae	*Coenonympha*	*pamphilus*	2011-04-12	Sicily	37,627	15,071
RVcoll11H745	-	Nymphalidae	*Coenonympha*	*pamphilus*	2011-06-13	Sicily	37,850	14,710
RVcoll11H921	-	Nymphalidae	*Coenonympha*	*pamphilus*	2011-06-16	Sicily	37,880	14,030
RVcoll11H733	-	Nymphalidae	*Coenonympha*	*pamphilus*	2011-06-13	Sicily	37,920	14,660
RVcoll11I063	-	Nymphalidae	*Coenonympha*	*pamphilus*	2011-06-18	Sicily	37,943	15,080
RVcoll11H705	-	Nymphalidae	*Coenonympha*	*pamphilus*	2011-06-12	Levanzo	38,000	12,330
RVcoll11H709	-	Nymphalidae	*Coenonympha*	*pamphilus*	2011-06-12	Levanzo	38,000	12,330
RVcoll11H704	-	Nymphalidae	*Coenonympha*	*pamphilus*	2011-06-12	Levanzo	38,010	12,330
RVcoll11H594	-	Nymphalidae	*Coenonympha*	*pamphilus*	2011-06-08	Sicily	38,172	13,325
RVcoll11D459	-	Nymphalidae	*Coenonympha*	*pamphilus*	2011-04-14	Sicily	38,241	15,562
RVcoll12Q890	-	Nymphalidae	*Coenonympha*	*pamphilus*	2012-07-31	Italy	38,250	15,950
RVcoll12Q891	-	Nymphalidae	*Coenonympha*	*pamphilus*	2012-07-31	Italy	38,250	15,950
RVcoll11H555	-	Nymphalidae	*Coenonympha*	*pamphilus*	2011-06-08	Sicily	38,312	13,341
RVcoll11D486	-	Nymphalidae	*Coenonympha*	*pamphilus*	2011-04-17	Italy	38,466	15,929
RVcoll11D497	-	Nymphalidae	*Coenonympha*	*pamphilus*	2011-04-18	Italy	38,722	16,013
RVcoll14A733	-	Nymphalidae	*Coenonympha*	*pamphilus*	2013-08-21	Italy	39,217	16,140
LEPSS00121	-	Nymphalidae	*Coenonympha*	*pamphilus*	2014-05-23	Italy	39,315	16,528
LEPSS00120	-	Nymphalidae	*Coenonympha*	*pamphilus*	2014-05-20	Italy	39,560	16,751
RVcoll11I220	-	Nymphalidae	*Coenonympha*	*pamphilus*	2011-06-20	Italy	39,930	16,150

RVcoll12N387	-	Pieridae	*Colias*	*croceus*	2012-05-30	Tunisia	33,942	8,173
RVcoll12N335	-	Pieridae	*Colias*	*croceus*	2012-05-29	Tunisia	35,053	9,257
RVcoll14L056	-	Pieridae	*Colias*	*croceus*	2009-05-13	Algeria	35,120	0,830
RVcoll14L046	-	Pieridae	*Colias*	*croceus*	2009-05-28	Algeria	35,190	1,130
RVcoll12N558	-	Pieridae	*Colias*	*croceus*	2012-05-11	Algeria	35,326	4,297
RVcoll11H623	-	Pieridae	*Colias*	*croceus*	2011-06-09	Lampedusa	35,520	12,550
RVcoll11H624	-	Pieridae	*Colias*	*croceus*	2011-06-09	Lampedusa	35,520	12,550
RVcoll11H625	-	Pieridae	*Colias*	*croceus*	2011-06-09	Lampedusa	35,520	12,550
RVcoll11H626	-	Pieridae	*Colias*	*croceus*	2011-06-09	Lampedusa	35,520	12,550
RVcoll08J930	-	Pieridae	*Colias*	*croceus*	2008-07-09	Tunisia	35,745	8,384
RVcoll10C592	-	Pieridae	*Colias*	*croceus*	2010-06-17	Maltese	35,927	14,442
RVcoll10C593	-	Pieridae	*Colias*	*croceus*	2010-06-17	Maltese	35,927	14,442
RVcoll11E151	-	Pieridae	*Colias*	*croceus*	2011-05-20	Maltese	36,042	14,274
RVcoll11E152	-	Pieridae	*Colias*	*croceus*	2011-05-20	Maltese	36,042	14,274
RVcoll11E153	-	Pieridae	*Colias*	*croceus*	2011-05-20	Maltese	36,042	14,274
RVcoll11E154	-	Pieridae	*Colias*	*croceus*	2011-05-20	Maltese	36,042	14,274
RVcoll12N618	-	Pieridae	*Colias*	*croceus*	2012-05-12	Algeria	36,257	5,245
RVcoll12N611	-	Pieridae	*Colias*	*croceus*	2012-05-12	Algeria	36,260	5,229
RVcoll12N236	-	Pieridae	*Colias*	*croceus*	2012-05-28	Tunisia	36,415	9,916
RVcoll12N966	-	Pieridae	*Colias*	*croceus*	2012-07-11	Algeria	36,459	4,135
RVcoll12N206	-	Pieridae	*Colias*	*croceus*	2012-05-28	Tunisia	36,651	10,161
RVcoll11D119	-	Pieridae	*Colias*	*croceus*	2011-04-08	Pantelleria	36,785	11,981
RVcoll11D121	-	Pieridae	*Colias*	*croceus*	2011-04-08	Pantelleria	36,785	11,981
RVcoll11D095	-	Pieridae	*Colias*	*croceus*	2011-04-08	Pantelleria	36,818	11,952
RVcoll13S943	-	Pieridae	*Colias*	*croceus*	2013-07-05	Tunisia	36,860	8,720
RVcoll12M360	-	Pieridae	*Colias*	*croceus*	2012-05-07	Sicily	37,150	14,201
RVcoll11D397	-	Pieridae	*Colias*	*croceus*	2011-04-13	Sicily	37,158	15,050
RVcoll13T399	-	Pieridae	*Colias*	*croceus*	2013-07-05	Tunisia	37,313	9,856
RVcollLD3039	-	Pieridae	*Colias*	*croceus*	2010-06-30	Sicily	37,627	13,050
RVcollLD3040	-	Pieridae	*Colias*	*croceus*	2010-06-30	Sicily	37,627	13,050
RVcoll11H920	-	Pieridae	*Colias*	*croceus*	2011-06-16	Sicily	37,880	14,030
RVcoll11D157	-	Pieridae	*Colias*	*croceus*	2011-04-10	Marettimo	37,961	12,067
RVcoll11D158	-	Pieridae	*Colias*	*croceus*	2011-04-10	Marettimo	37,961	12,067
RVcoll11H694	-	Pieridae	*Colias*	*croceus*	2011-06-12	Levanzo	38,000	12,330
RVcoll11H695	-	Pieridae	*Colias*	*croceus*	2011-06-12	Levanzo	38,000	12,330
RVcoll11H696	-	Pieridae	*Colias*	*croceus*	2011-06-12	Levanzo	38,010	12,330
RVcoll11H538	-	Pieridae	*Colias*	*croceus*	2011-06-08	Sicily	38,093	13,260
RVcoll11D407	-	Pieridae	*Colias*	*croceus*	2011-04-14	Sicily	38,130	15,496
RVcoll12Q886	-	Pieridae	*Colias*	*croceus*	2012-07-31	Italy	38,250	15,950
RVcoll10C539	-	Pieridae	*Colias*	*croceus*	2010-06-15	Italy	38,372	16,236
RVcoll11H789	-	Pieridae	*Colias*	*croceus*	2011-06-14	Vulcano	38,390	14,970
RVcoll11H790		Pieridae	*Colias*	*croceus*	2011-06-14	Vulcano	38,390	14,970
RVcoll10C678		Pieridae	*Colias*	*croceus*	2010-06-30	Lipari	38,482	14,931
RVcoll11H869		Pieridae	*Colias*	*croceus*	2011-06-15	Salina	38,560	14,830
RVcoll10C685		Pieridae	*Colias*	*croceus*	2012-07-01	Stromboli	38,803	15,228
LEPSS00038		Pieridae	*Colias*	*croceus*	2014-05-27	Italy	39,323	16,475
LEPSS00039		Pieridae	*Colias*	*croceus*	2014-05-27	Italy	39,323	16,475
RVcoll11I251		Pieridae	*Colias*	*croceus*	2011-06-20	Italy	39,840	16,080
RVcoll11I252		Pieridae	*Colias*	*croceus*	2011-06-20	Italy	39,860	16,070
RVcoll12Q787		Pieridae	*Colias*	*croceus*	2012-07-30	Italy	39,930	16,170
RVcoll14B037		Nymphalidae	*Danaus*	*chrysippus*	2013-03-07	Algeria	33,104	6,088
RVcoll14B038		Nymphalidae	*Danaus*	*chrysippus*	2013-03-07	Algeria	33,104	6,088
RVcoll12N412	-	Nymphalidae	*Danaus*	*chrysippus*	2012-05-30	Tunisia	34,337	9,051
RVcoll12N367		Nymphalidae	*Danaus*	*chrysippus*	2012-05-29	Tunisia	34,345	8,329
RVcoll14L050		Nymphalidae	*Danaus*	*chrysippus*	2009-05-13	Algeria	35,120	0,830
RVcoll12N323		Nymphalidae	*Danaus*	*chrysippus*	2012-05-29	Tunisia	35,425	9,571
RVcoll11H613		Nymphalidae	*Danaus*	*chrysippus*	2011-06-09	Lampedusa	35,520	12,550
RVcoll11H614		Nymphalidae	*Danaus*	*chrysippus*	2011-06-09	Lampedusa	35,520	12,550
LEPSS00230		Nymphalidae	*Danaus*	*chrysippus*	2012-09-09	Sicily	38,280	15,590
RVcoll11D351		Pieridae	*Euchloe*	*ausonia*	2011-04-13	Sicily	37,197	15,118
RVcoll11D307		Pieridae	*Euchloe*	*ausonia*	2011-04-12	Sicily	37,627	15,071
RVcoll11D255		Pieridae	*Euchloe*	*ausonia*	2011-04-11	Sicily	37,838	13,429
RVcoll16A045		Pieridae	*Euchloe*	*ausonia*	2015-04-07	Sicily	37,840	13,991
RVcoll12M264	-	Pieridae	*Euchloe*	*ausonia*	2012-05-04	Italy	38,000	15,801
RVcoll12M270		Pieridae	*Euchloe*	*ausonia*	2012-05-04	Italy	38,000	15,801
RVcoll12M269		Pieridae	*Euchloe*	*ausonia*	2012-05-04	Italy	38,010	15,801
RVcoll10C617		Pieridae	*Euchloe*	*ausonia*	2010-06-26	Italy	38,122	15,675
RVcoll11D409		Pieridae	*Euchloe*	*ausonia*	2011-04-14	Sicily	38,130	15,496
RVcoll11D075		Pieridae	*Euchloe*	*ausonia*	2011-04-07	Sicily	38,138	13,138
RVcoll12M504		Pieridae	*Euchloe*	*ausonia*	2012-05-12	Italy	38,601	16,184
RVcoll14V853		Pieridae	*Euchloe*	*ausonia*	2015-04-30	Ustica	38,709	13,179
RVcoll14V854		Pieridae	*Euchloe*	*ausonia*	2015-04-30	Ustica	38,709	13,179
LEPSS00027		Pieridae	*Euchloe*	*ausonia*	2014-05-20	Italy	39,560	16,751
LEPSS00028		Pieridae	*Euchloe*	*ausonia*	2014-05-20	Italy	39,560	16,751
RVcoll12N385		Hesperiidae	*Gegenes*	*nostrodamus*	2012-05-30	Tunisia	33,942	8,173
RVcoll11H620		Hesperiidae	*Gegenes*	*nostrodamus*	2011-06-09	Lampedusa	35,520	12,550
RVcoll11H621		Hesperiidae	*Gegenes*	*nostrodamus*	2011-06-09	Lampedusa	35,520	12,550
RVcoll11H622		Hesperiidae	*Gegenes*	*nostrodamus*	2011-06-09	Lampedusa	35,520	12,550
RVcoll11I026		Hesperiidae	*Gegenes*	*nostrodamus*	2011-06-17	Sicily	37,840	13,920
RVcoll11I025		Hesperiidae	*Gegenes*	*nostrodamus*	2011-06-17	Sicily	37,850	13,920
LEPSS00239		Hesperiidae	*Gegenes*	*nostrodamus*	2012-08-04	Sicily	38,269	15,652
RVcollLD2569		Hesperiidae	*Gegenes*	*nostrodamus*	2009-06-30	Lipari	38,482	14,931
RVcoll14A097		Hesperiidae	*Gegenes*	*nostrodamus*	2013-08-20	Italy	39,483	16,801
RVcoll15A816		Hesperiidae	*Gegenes*	*nostrodamus*	2015-06-05	Italy	39,555	16,747

RVcoll14V915		Hesperiidae	*Gegenes*	*nostrodamus?*	2015-06-05	Italy	39,555	16,747
RVcollLD2721		Hesperiidae	*Gegenes*	*pumilio*	2010-06-17	Maltese	35,902	14,407
RVcoll10C587		Hesperiidae	*Gegenes*	*pumilio*	2010-06-17	Maltese	35,927	14,442
RVcollLD2713	-	Hesperiidae	*Gegenes*	*pumilio*	2010-06-17	Maltese	35,927	14,442
RVcollLD2714		Hesperiidae	*Gegenes*	*pumilio*	2010-06-17	Maltese	35,927	14,442
RVcoll11D138		Hesperiidae	*Gegenes*	*pumilio*	2011-04-09	Pantelleria	36,761	11,998
RVcoll11H658		Hesperiidae	*Gegenes*	*pumilio*	2011-06-11	Pantelleria	36,790	12,000
RVcoll13T302		Hesperiidae	*Gegenes*	*pumilio*	2013-07-05	Tunisia	36,860	8,720
RVcoll08R544		Hesperiidae	*Gegenes*	*pumilio*	2011-04-13	Sicily	37,158	15,049
RVcoll08R543		Hesperiidae	*Gegenes*	*pumilio*	2011-04-13	Sicily	37,158	15,050
RVcoll08R547		Hesperiidae	*Gegenes*	*pumilio*	2011-04-13	Sicily	37,158	15,050
RVcoll13T300		Hesperiidae	*Gegenes*	*pumilio*	2013-07-04	Tunisia	37,164	10,110
RVcoll12M382		Hesperiidae	*Gegenes*	*pumilio*	2012-05-10	Sicily	37,551	13,684
RVcoll11J732		Hesperiidae	*Gegenes*	*pumilio*	2011-07-22	Sicily	37,850	13,430
RVcoll11J733	-	Hesperiidae	*Gegenes*	*pumilio*	2011-07-22	Sicily	37,850	13,430
RVcoll14N608		Hesperiidae	*Gegenes*	*pumilio*	2014-09-27	Levanzo	37,987	12,342
RVcoll10C618		Hesperiidae	*Gegenes*	*pumilio*	2010-06-26	Italy	38,122	15,675
RVcoll10C619		Hesperiidae	*Gegenes*	*pumilio*	2010-06-26	Italy	38,122	15,675
RVcoll11H867		Hesperiidae	*Gegenes*	*pumilio*	2011-06-15	Salina	38,560	14,830
RVcollLD2401		Hesperiidae	*Gegenes*	*pumilio*	2010-06-30	Ustica	38,709	13,179
LEPSS00110		Hesperiidae	*Gegenes*	*pumilio*	2014-09-08	Italy	39,616	16,766
LEPSS00109		Hesperiidae	*Gegenes*	*pumilio*	2014-09-18	Italy	39,616	16,766
RVcoll12N422		Pieridae	*Gonepteryx*	*cleopatra*	2012-05-31	Tunisia	35,199	8,674
RVcoll14L084		Pieridae	*Gonepteryx*	*cleopatra*	2009-05-10	Algeria	35,280	0,920
RVcollLD2732		Pieridae	*Gonepteryx*	*cleopatra*	2010-06-17	Maltese	35,858	14,399
RVcoll11E110		Pieridae	*Gonepteryx*	*cleopatra*	2011-05-20	Maltese	36,042	14,274
RVcoll12N299	-	Pieridae	*Gonepteryx*	*cleopatra*	2012-05-28	Tunisia	36,116	9,656
RVcoll12N977		Pieridae	*Gonepteryx*	*cleopatra*	2012-07-11	Algeria	36,492	4,266
RVcoll12N910		Pieridae	*Gonepteryx*	*cleopatra*	2012-06-01	Algeria	36,592	4,611
RVcoll12N914		Pieridae	*Gonepteryx*	*cleopatra*	2012-06-08	Algeria	36,592	4,611
RVcoll14A111		Pieridae	*Gonepteryx*	*cleopatra*	2013-07-07	Tunisia	36,726	8,705
RVcoll12N948		Pieridae	*Gonepteryx*	*cleopatra*	2012-06-22	Algeria	36,763	5,102
RVcoll12N532		Pieridae	*Gonepteryx*	*cleopatra*	2012-06-02	Tunisia	36,880	8,910
RVcoll11D298		Pieridae	*Gonepteryx*	*cleopatra*	2011-04-12	Sicily	37,627	15,071
RVcoll11I029		Pieridae	*Gonepteryx*	*cleopatra*	2011-06-17	Sicily	37,840	13,920
RVcoll11H686		Pieridae	*Gonepteryx*	*cleopatra*	2011-06-12	Levanzo	38,000	12,330
RVcoll11H687		Pieridae	*Gonepteryx*	*cleopatra*	2011-06-12	Levanzo	38,010	12,330
RVcoll11I115	-	Pieridae	*Gonepteryx*	*cleopatra*	2011-06-19	Italy	38,201	15,980
RVcollLD2834		Pieridae	*Gonepteryx*	*cleopatra*	2010-06-27	Sicily	38,208	15,499
RVcoll11H601		Pieridae	*Gonepteryx*	*cleopatra*	2011-06-08	Sicily	38,226	13,222
RVcoll11H559		Pieridae	*Gonepteryx*	*cleopatra*	2011-06-08	Sicily	38,312	13,341
RVcoll15A812		Pieridae	*Gonepteryx*	*cleopatra*	2015-06-05	Italy	39,555	16,747
RVcoll15A819		Pieridae	*Gonepteryx*	*cleopatra*	2015-06-05	Italy	39,555	16,747
RVcoll14L090		Nymphalidae	*Hipparchia*	*algirica*	2009-05-28	Algeria	35,190	1,130
RVcoll08J911		Nymphalidae	*Hipparchia*	*algirica*	2008-07-08	Tunisia	35,205	8,677
RVcoll14B076		Nymphalidae	*Hipparchia*	*algirica*	2013-05-24	Algeria	36,019	4,236
RVcoll12N898		Nymphalidae	*Hipparchia*	*algirica*	2012-05-27	Algeria	36,442	4,207
RVcoll11H973		Nymphalidae	*Hipparchia*	*blachieri*	2011-06-16	Sicily	37,710	13,970
RVcoll12R177		Nymphalidae	*Hipparchia*	*blachieri*	2012-08-04	Sicily	37,840	13,420
RVcoll10C651		Nymphalidae	*Hipparchia*	*blachieri*	2010-06-29	Sicily	37,845	13,434
RVcollLD2112		Nymphalidae	*Hipparchia*	*blachieri*	2010-06-30	Sicily	37,868	13,384
RVcoll08M165		Nymphalidae	*Hipparchia*	*blachieri*	2008-07-11	Sicily	37,890	13,990
RVcollLD2109		Nymphalidae	*Hipparchia*	*blachieri*	2010-06-28	Sicily	37,909	13,977
RVcoll11H731		Nymphalidae	*Hipparchia*	*blachieri*	2011-06-13	Sicily	37,920	14,660
RVcoll11H546		Nymphalidae	*Hipparchia*	*blachieri*	2011-06-08	Sicily	38,093	13,260
RVcoll08M162		Nymphalidae	*Hipparchia*	*leighebi*	2008-07-10	Vulcano	38,390	14,970
RVcoll11H840		Nymphalidae	*Hipparchia*	*leighebi*	2011-06-14	Vulcano	38,390	14,970
RVcoll11H841		Nymphalidae	*Hipparchia*	*leighebi*	2011-06-14	Vulcano	38,390	14,970
RVcoll11H845		Nymphalidae	*Hipparchia*	*leighebi*	2011-06-14	Vulcano	38,390	14,970
RVcollLD3213		Nymphalidae	*Hipparchia*	*leighebi*	2009-06-30	Lipari	38,482	14,931
RVcoll11H873	-	Nymphalidae	*Hipparchia*	*leighebi*	2011-06-15	Salina	38,560	14,830
RVcoll11H872		Nymphalidae	*Hipparchia*	*leighebi*	2011-06-15	Salina	38,560	14,840
RVcoll09T557		Nymphalidae	*Hipparchia*	*leighebi*	2009-07-02	Stromboli	38,800	15,230
RVcoll09T558		Nymphalidae	*Hipparchia*	*leighebi*	2009-07-02	Stromboli	38,800	15,230
RVcoll10C686		Nymphalidae	*Hipparchia*	*leighebi*	2012-07-01	Stromboli	38,803	15,228
RVcollLD2102		Nymphalidae	*Hipparchia*	*neapolitana*	2010-06-26	Italy	38,150	15,810
RVcollLD2100		Nymphalidae	*Hipparchia*	*neapolitana*	2010-06-26	Italy	38,160	15,880
RVcollLD2101		Nymphalidae	*Hipparchia*	*neapolitana*	2010-06-26	Italy	38,160	15,880
RVcollLD2103		Nymphalidae	*Hipparchia*	*neapolitana*	2010-06-26	Italy	38,160	15,880
RVcoll11H751		Nymphalidae	*Hipparchia*	*semele*	2011-06-13	Sicily	37,850	14,710
RVcoll08M171		Nymphalidae	*Hipparchia*	*semele*	2008-07-11	Sicily	37,890	13,977
RVcoll08M168	-	Nymphalidae	*Hipparchia*	*semele*	2008-07-11	Sicily	37,890	13,990
RVcoll10C665		Nymphalidae	*Hipparchia*	*semele*	2010-06-29	Italy	38,084	15,834
LEPSS00273		Nymphalidae	*Hipparchia*	*semele*	2015-07-10	Italy	39,215	16,139
LEPSS00129		Nymphalidae	*Hipparchia*	*semele*	2014-08-20	Italy	39,301	16,438
LEPSS00128		Nymphalidae	*Hipparchia*	*semele*	2014-07-17	Italy	39,376	16,600
RVcoll07E002		Nymphalidae	*Hipparchia*	*semele*	2007-07-10	Italy	39,943	16,147
RVcoll12N405		Lycaenidae	*Lampides*	*boeticus*	2012-05-30	Tunisia	34,337	9,051
RVcoll12N407		Lycaenidae	*Lampides*	*boeticus*	2012-05-30	Tunisia	34,337	9,051
RVcoll12N369		Lycaenidae	*Lampides*	*boeticus*	2012-05-29	Tunisia	34,345	8,329
RVcoll12N331		Lycaenidae	*Lampides*	*boeticus*	2012-05-29	Tunisia	35,053	9,257
RVcoll14L081		Lycaenidae	*Lampides*	*boeticus*	2007-05-23	Algeria	35,280	0,920
RVcoll11H612		Lycaenidae	*Lampides*	*boeticus*	2011-06-09	Lampedusa	35,520	12,550
RVcoll14L130	-	Lycaenidae	*Lampides*	*boeticus*	2013-03-10	Algeria	35,750	4,550

RVcoll10C588		Lycaenidae	*Lampides*	*boeticus*	2010-06-17	Maltese	35,927	14,442
RVcoll10C589		Lycaenidae	*Lampides*	*boeticus*	2010-06-17	Maltese	35,927	14,442
RVcoll11E131		Lycaenidae	*Lampides*	*boeticus*	2011-05-20	Maltese	36,042	14,274
RVcoll11E132		Lycaenidae	*Lampides*	*boeticus*	2011-05-20	Maltese	36,042	14,274
RVcoll11E133		Lycaenidae	*Lampides*	*boeticus*	2011-05-20	Maltese	36,042	14,274
RVcoll11E134		Lycaenidae	*Lampides*	*boeticus*	2011-05-20	Maltese	36,042	14,274
RVcoll12N256		Lycaenidae	*Lampides*	*boeticus*	2012-05-28	Tunisia	36,294	9,811
RVcoll12N897		Lycaenidae	*Lampides*	*boeticus*	2012-05-27	Algeria	36,442	4,207
RVcoll13T603		Lycaenidae	*Lampides*	*boeticus*	2013-07-10	Tunisia	36,713	10,363
RVcoll13S949		Lycaenidae	*Lampides*	*boeticus*	2013-07-05	Tunisia	36,741	8,680
RVcoll11H661	-	Lycaenidae	*Lampides*	*boeticus*	2011-06-11	Pantelleria	36,790	12,000
RVcoll11H662		Lycaenidae	*Lampides*	*boeticus*	2011-06-11	Pantelleria	36,790	12,000
RVcoll11H663		Lycaenidae	*Lampides*	*boeticus*	2011-06-11	Pantelleria	36,790	12,000
RVcoll11H665		Lycaenidae	*Lampides*	*boeticus*	2011-06-11	Pantelleria	36,790	12,000
RVcoll12Q594		Lycaenidae	*Lampides*	*boeticus*	2012-08-01	Sicily	37,800	15,067
RVcoll11J687		Lycaenidae	*Lampides*	*boeticus*	2011-07-21	Sicily	37,840	14,010
RVcoll11H311		Lycaenidae	*Lampides*	*boeticus*	2011-07-20	Sicily	37,860	14,060
RVcoll11H286		Lycaenidae	*Lampides*	*boeticus*	2011-07-20	Sicily	37,880	14,030
RVcoll12R029		Lycaenidae	*Lampides*	*boeticus*	2012-08-02	Sicily	37,980	14,870
RVcoll11H692		Lycaenidae	*Lampides*	*boeticus*	2011-06-12	Levanzo	38,000	12,330
RVcoll11H693	-	Lycaenidae	*Lampides*	*boeticus*	2011-06-12	Levanzo	38,000	12,330
RVcoll11H691		Lycaenidae	*Lampides*	*boeticus*	2011-06-12	Levanzo	38,010	12,330
RVcoll11J740		Lycaenidae	*Lampides*	*boeticus*	2011-07-22	Sicily	38,100	13,240
RVcoll11I154		Lycaenidae	*Lampides*	*boeticus*	2011-06-19	Italy	38,160	15,880
RVcoll12Q935		Lycaenidae	*Lampides*	*boeticus*	2012-07-31	Italy	38,184	15,984
RVcoll14N643		Lycaenidae	*Lampides*	*boeticus*	2014-09-30	Vulcano	38,380	14,970
RVcollLD2566		Lycaenidae	*Lampides*	*boeticus*	2009-06-30	Lipari	38,482	14,931
RVcollLD2567		Lycaenidae	*Lampides*	*boeticus*	2009-06-30	Lipari	38,482	14,931
RVcoll10C679		Lycaenidae	*Lampides*	*boeticus*	2010-06-30	Lipari	38,482	14,931
RVcollLD2394		Lycaenidae	*Lampides*	*boeticus*	2010-06-30	Ustica	38,709	13,179
RVcoll10C683		Lycaenidae	*Lampides*	*boeticus*	2012-07-01	Stromboli	38,803	15,228
RVcoll14A711		Lycaenidae	*Lampides*	*boeticus*	2013-08-21	Italy	39,217	16,140
LEPSS00077		Lycaenidae	*Lampides*	*boeticus*	2014-07-25	Italy	39,335	16,399
LEPSS00078		Lycaenidae	*Lampides*	*boeticus*	2014-07-17	Italy	39,395	16,599
RVcoll12N334		Nymphalidae	*Lasiommata*	*megera*	2012-05-29	Tunisia	35,053	9,257
RVcoll12N424		Nymphalidae	*Lasiommata*	*megera*	2012-05-31	Tunisia	35,199	8,674
RVcoll14L064		Nymphalidae	*Lasiommata*	*megera*	2014-05-15	Algeria	35,280	1,070
RVcoll14V561		Nymphalidae	*Lasiommata*	*megera*	1997-05-28	Lampedusa	35,510	12,560
RVcoll14L124		Nymphalidae	*Lasiommata*	*megera*	2014-05-04	Algeria	35,860	4,750
RVcollLD1987		Nymphalidae	*Lasiommata*	*megera*	2010-06-17	Maltese	35,927	14,442
RVcollLD1988		Nymphalidae	*Lasiommata*	*megera*	2010-06-17	Maltese	35,927	14,442
RVcollLD1989		Nymphalidae	*Lasiommata*	*megera*	2010-06-17	Maltese	35,927	14,442
RVcollLD1990		Nymphalidae	*Lasiommata*	*megera*	2010-06-17	Maltese	35,927	14,442
RVcoll11E126		Nymphalidae	*Lasiommata*	*megera*	2011-05-20	Maltese	36,042	14,274
RVcoll11E127		Nymphalidae	*Lasiommata*	*megera*	2011-05-20	Maltese	36,042	14,274
RVcoll11E128		Nymphalidae	*Lasiommata*	*megera*	2011-05-20	Maltese	36,042	14,274
RVcoll11E129		Nymphalidae	*Lasiommata*	*megera*	2011-05-20	Maltese	36,042	14,274
RVcoll13T316		Nymphalidae	*Lasiommata*	*megera*	2013-07-08	Tunisia	36,225	8,771
RVcoll12N281		Nymphalidae	*Lasiommata*	*megera*	2012-05-28	Tunisia	36,257	9,769
RVcoll12N975		Nymphalidae	*Lasiommata*	*megera*	2012-07-11	Algeria	36,483	4,251
RVcoll12N907		Nymphalidae	*Lasiommata*	*megera*	2012-06-01	Algeria	36,592	4,611
RVcoll12N933		Nymphalidae	*Lasiommata*	*megera*	2012-06-16	Algeria	36,630	4,598
RVcoll13T609		Nymphalidae	*Lasiommata*	*megera*	2013-07-10	Tunisia	36,713	10,363
RVcoll13T306		Nymphalidae	*Lasiommata*	*megera*	2013-07-05	Tunisia	36,741	8,680
RVcoll12N506		Nymphalidae	*Lasiommata*	*megera*	2012-06-01	Tunisia	36,764	8,666
RVcoll11D124		Nymphalidae	*Lasiommata*	*megera*	2011-04-08	Pantelleria	36,782	12,000
RVcoll11D125		Nymphalidae	*Lasiommata*	*megera*	2011-04-08	Pantelleria	36,782	12,000
RVcoll11D126		Nymphalidae	*Lasiommata*	*megera*	2011-04-08	Pantelleria	36,782	12,000
RVcoll11D134		Nymphalidae	*Lasiommata*	*megera*	2011-04-09	Pantelleria	36,782	12,000
RVcoll11H672		Nymphalidae	*Lasiommata*	*megera*	2011-06-11	Pantelleria	36,790	12,000
RVcoll11H673	-	Nymphalidae	*Lasiommata*	*megera*	2011-06-11	Pantelleria	36,790	12,000
RVcoll11H674		Nymphalidae	*Lasiommata*	*megera*	2011-06-11	Pantelleria	36,790	12,000
RVcoll13T320		Nymphalidae	*Lasiommata*	*megera*	2013-07-10	Tunisia	36,837	10,584
RVcollLD0115		Nymphalidae	*Lasiommata*	*megera*	2008-04-26	Tunisia	37,177	9,725
RVcollLD0116		Nymphalidae	*Lasiommata*	*megera*	2008-04-26	Tunisia	37,177	9,725
RVcoll11D355		Nymphalidae	*Lasiommata*	*megera*	2011-04-13	Sicily	37,197	15,118
RVcoll12M339		Nymphalidae	*Lasiommata*	*megera*	2012-05-07	Sicily	37,201	14,167
RVcoll12M391		Nymphalidae	*Lasiommata*	*megera*	2012-05-10	Sicily	37,493	13,684
RVcoll12M392		Nymphalidae	*Lasiommata*	*megera*	2012-05-10	Sicily	37,493	13,684
RVcoll11D321		Nymphalidae	*Lasiommata*	*megera*	2011-04-12	Sicily	37,627	15,071
RVcollLD2133	-	Nymphalidae	*Lasiommata*	*megera*	2010-06-30	Sicily	37,699	13,144
RVcoll11D191		Nymphalidae	*Lasiommata*	*megera*	2011-04-10	Marettimo	37,961	12,067
RVcoll11D192		Nymphalidae	*Lasiommata*	*megera*	2011-04-10	Marettimo	37,961	12,067
RVcoll11D193		Nymphalidae	*Lasiommata*	*megera*	2011-04-10	Marettimo	37,961	12,067
RVcoll11D194		Nymphalidae	*Lasiommata*	*megera*	2011-04-10	Marettimo	37,961	12,067
RVcoll11D195		Nymphalidae	*Lasiommata*	*megera*	2011-04-10	Marettimo	37,961	12,067
RVcoll11D196		Nymphalidae	*Lasiommata*	*megera*	2011-04-10	Marettimo	37,961	12,067
RVcoll11D197		Nymphalidae	*Lasiommata*	*megera*	2011-04-10	Marettimo	37,961	12,067
RVcoll11H718		Nymphalidae	*Lasiommata*	*megera*	2011-06-12	Levanzo	38,000	12,330
RVcoll11H719		Nymphalidae	*Lasiommata*	*megera*	2011-06-12	Levanzo	38,000	12,330
RVcoll11H720		Nymphalidae	*Lasiommata*	*megera*	2011-06-12	Levanzo	38,000	12,330
RVcollLD1884		Nymphalidae	*Lasiommata*	*megera*	2010-05-05	Italy	38,120	15,670
RVcollLD1885		Nymphalidae	*Lasiommata*	*megera*	2010-05-05	Italy	38,120	15,670
RVcoll11D031		Nymphalidae	*Lasiommata*	*megera*	2011-04-07	Sicily	38,139	13,126

RVcollLD0204		Nymphalidae	*Lasiommata*	*megera*	2008-05-25	Sicily	38,140	15,280
RVcollLD2081		Nymphalidae	*Lasiommata*	*megera*	2010-06-27	Sicily	38,170	15,470
RVcoll11D482		Nymphalidae	*Lasiommata*	*megera*	2011-04-16	Italy	38,233	15,717
RVcoll11D446		Nymphalidae	*Lasiommata*	*megera*	2011-04-14	Sicily	38,238	15,528
RVcoll11H852		Nymphalidae	*Lasiommata*	*megera*	2011-06-14	Vulcano	38,380	14,980
RVcoll11H853	-	Nymphalidae	*Lasiommata*	*megera*	2011-06-14	Vulcano	38,380	14,980
RVcoll12M494		Nymphalidae	*Lasiommata*	*megera*	2012-05-12	Italy	38,383	16,167
RVcollLD1608		Nymphalidae	*Lasiommata*	*megera*	2009-06-30	Lipari	38,496	14,933
RVcollLD1611		Nymphalidae	*Lasiommata*	*megera*	2009-06-30	Lipari	38,496	14,933
RVcoll09T556		Nymphalidae	*Lasiommata*	*megera*	2009-07-01	Lipari	38,496	14,933
RVcoll11H909		Nymphalidae	*Lasiommata*	*megera*	2011-06-15	Salina	38,560	14,830
RVcoll11H910		Nymphalidae	*Lasiommata*	*megera*	2011-06-15	Salina	38,560	14,830
RVcoll11H911		Nymphalidae	*Lasiommata*	*megera*	2011-06-15	Salina	38,560	14,830
RVcoll11H912		Nymphalidae	*Lasiommata*	*megera*	2011-06-15	Salina	38,560	14,830
RVcoll11H913		Nymphalidae	*Lasiommata*	*megera*	2011-06-15	Salina	38,560	14,830
RVcoll11H914	-	Nymphalidae	*Lasiommata*	*megera*	2011-06-15	Salina	38,560	14,830
RVcoll11H915		Nymphalidae	*Lasiommata*	*megera*	2011-06-15	Salina	38,560	14,830
RVcollLD2050		Nymphalidae	*Lasiommata*	*megera*	2010-06-30	Ustica	38,709	13,179
RVcollLD2051		Nymphalidae	*Lasiommata*	*megera*	2010-06-30	Ustica	38,709	13,179
RVcollLD2052		Nymphalidae	*Lasiommata*	*megera*	2010-06-30	Ustica	38,709	13,179
RVcollLD1670		Nymphalidae	*Lasiommata*	*megera*	2009-07-01	Stromboli	38,803	15,228
RVcollLD1673		Nymphalidae	*Lasiommata*	*megera*	2009-07-01	Stromboli	38,803	15,228
RVcoll14A784		Nymphalidae	*Lasiommata*	*megera*	2013-08-21	Italy	39,251	16,225
LEPSS00123		Nymphalidae	*Lasiommata*	*megera*	2014-05-27	Italy	39,323	16,475
LEPSS00122		Nymphalidae	*Lasiommata*	*megera*	2014-07-17	Italy	39,376	16,600
RVcoll14A704	-	Nymphalidae	*Lasiommata*	*megera*	2013-08-20	Italy	39,720	16,525
LEPSS00196		Nymphalidae	*Lasiommata*	*megera*	2015-05-20	Italy	39,828	16,077
RVcoll12N392		Lycaenidae	*Leptotes*	*pirithous*	2012-05-30	Tunisia	33,407	9,008
RVcoll12N576		Lycaenidae	*Leptotes*	*pirithous*	2012-05-18	Algeria	35,326	4,297
RVcoll11H611		Lycaenidae	*Leptotes*	*pirithous*	2011-06-09	Lampedusa	35,520	12,550
RVcoll11H616		Lycaenidae	*Leptotes*	*pirithous*	2011-06-09	Lampedusa	35,520	12,550
RVcoll10C603		Lycaenidae	*Leptotes*	*pirithous*	2010-06-17	Maltese	35,902	14,407
RVcoll10C604		Lycaenidae	*Leptotes*	*pirithous*	2010-06-17	Maltese	35,902	14,407
RVcoll10C605		Lycaenidae	*Leptotes*	*pirithous*	2010-06-17	Maltese	35,902	14,407
RVcoll12R417		Lycaenidae	*Leptotes*	*pirithous*	2010-06-17	Maltese	35,927	14,442
RVcoll12N309		Lycaenidae	*Leptotes*	*pirithous*	2012-05-28	Tunisia	36,116	9,656
RVcoll08J915		Lycaenidae	*Leptotes*	*pirithous*	2008-07-09	Tunisia	36,284	8,973
RVcoll11D139		Lycaenidae	*Leptotes*	*pirithous*	2011-04-09	Pantelleria	36,761	11,998
RVcoll11D140		Lycaenidae	*Leptotes*	*pirithous*	2011-04-09	Pantelleria	36,761	11,998
RVcoll13T622		Lycaenidae	*Leptotes*	*pirithous*	2013-07-10	Tunisia	36,837	10,584
RVcoll12N541		Lycaenidae	*Leptotes*	*pirithous*	2012-06-02	Tunisia	36,992	9,078
RVcoll13S923		Lycaenidae	*Leptotes*	*pirithous*	2013-07-05	Tunisia	37,010	9,625
RVcoll12Q593		Lycaenidae	*Leptotes*	*pirithous*	2012-08-01	Sicily	37,840	14,990
RVcoll11D169		Lycaenidae	*Leptotes*	*pirithous*	2011-04-10	Marettimo	37,961	12,067
RVcoll11D170		Lycaenidae	*Leptotes*	*pirithous*	2011-04-10	Marettimo	37,961	12,067
RVcoll11D180		Lycaenidae	*Leptotes*	*pirithous*	2011-04-10	Marettimo	37,961	12,067
RVcoll14N602		Lycaenidae	*Leptotes*	*pirithous*	2014-09-27	Levanzo	37,987	12,342
RVcoll10C615		Lycaenidae	*Leptotes*	*pirithous*	2010-06-26	Italy	38,122	15,675
RVcoll14N639		Lycaenidae	*Leptotes*	*pirithous*	2014-09-30	Vulcano	38,380	14,970
RVcoll14N669		Lycaenidae	*Leptotes*	*pirithous*	2014-10-01	Salina	38,570	14,830
RVcoll14W001		Lycaenidae	*Leptotes*	*pirithous*	2013-07-13	Ustica	38,709	13,179
LEPSS00279		Lycaenidae	*Leptotes*	*pirithous*	2015-07-13	Italy	38,732	16,229
RVcoll14N723		Lycaenidae	*Leptotes*	*pirithous*	2014-10-03	Stromboli	38,800	15,210
RVcoll07E046		Lycaenidae	*Leptotes*	*pirithous*	2007-07-11	Italy	39,336	16,358
LEPSS00098	-	Lycaenidae	*Leptotes*	*pirithous*	2014-09-18	Italy	39,616	16,766
LEPSS00099		Lycaenidae	*Leptotes*	*pirithous*	2014-09-18	Italy	39,616	16,766
RVcoll14A708		Lycaenidae	*Leptotes*	*pirithous*	2013-08-20	Italy	39,720	16,525
RVcoll14A709		Lycaenidae	*Leptotes*	*pirithous*	2013-08-20	Italy	39,720	16,525
RVcoll14A710		Lycaenidae	*Leptotes*	*pirithous*	2013-08-20	Italy	39,720	16,525
RVcoll14L060		Lycaenidae	*Lycaena*	*phlaeas*	2009-05-28	Algeria	35,190	1,130
RVcoll14L105		Lycaenidae	*Lycaena*	*phlaeas*	2007-05-23	Algeria	35,280	0,920
RVcoll13T562		Lycaenidae	*Lycaena*	*phlaeas*	2013-07-09	Tunisia	35,447	9,224
RVcoll11H615		Lycaenidae	*Lycaena*	*phlaeas*	2011-06-09	Lampedusa	35,520	12,550
RVcoll08J929		Lycaenidae	*Lycaena*	*phlaeas*	2008-07-09	Tunisia	35,750	8,361
RVcoll12N306	-	Lycaenidae	*Lycaena*	*phlaeas*	2012-05-28	Tunisia	36,116	9,656
RVcoll12N251		Lycaenidae	*Lycaena*	*phlaeas*	2012-05-28	Tunisia	36,294	9,811
RVcoll13T590		Lycaenidae	*Lycaena*	*phlaeas*	2013-07-10	Tunisia	36,333	10,093
RVcoll12N995		Lycaenidae	*Lycaena*	*phlaeas*	2012-07-12	Algeria	36,479	3,998
RVcoll12N911		Lycaenidae	*Lycaena*	*phlaeas*	2012-06-08	Algeria	36,592	4,611
RVcoll12N956		Lycaenidae	*Lycaena*	*phlaeas*	2012-06-29	Algeria	36,625	4,598
RVcoll12N931		Lycaenidae	*Lycaena*	*phlaeas*	2012-06-16	Algeria	36,630	4,598
RVcoll12N477		Lycaenidae	*Lycaena*	*phlaeas*	2012-06-01	Tunisia	36,731	8,710
RVcoll11H666		Lycaenidae	*Lycaena*	*phlaeas*	2011-06-11	Pantelleria	36,790	12,000
RVcoll11H667		Lycaenidae	*Lycaena*	*phlaeas*	2011-06-11	Pantelleria	36,790	12,000
RVcoll11H668	-	Lycaenidae	*Lycaena*	*phlaeas*	2011-06-11	Pantelleria	36,790	12,000
RVcoll11D153		Lycaenidae	*Lycaena*	*phlaeas*	2011-04-09	Pantelleria	36,819	12,006
RVcoll13T390		Lycaenidae	*Lycaena*	*phlaeas*	2013-07-04	Tunisia	37,164	10,110
RVcoll11D360		Lycaenidae	*Lycaena*	*phlaeas*	2011-04-13	Sicily	37,197	15,118
RVcoll12M340		Lycaenidae	*Lycaena*	*phlaeas*	2012-05-07	Sicily	37,201	14,167
RVcollLD2983		Lycaenidae	*Lycaena*	*phlaeas*	2010-06-30	Sicily	37,699	13,144
RVcoll12R162		Lycaenidae	*Lycaena*	*phlaeas*	2012-08-04	Sicily	37,840	13,420
RVcoll11H746		Lycaenidae	*Lycaena*	*phlaeas*	2011-06-13	Sicily	37,850	14,710
RVcoll12Q926		Lycaenidae	*Lycaena*	*phlaeas*	2012-07-31	Italy	38,067	15,817
RVcoll12Q927		Lycaenidae	*Lycaena*	*phlaeas*	2012-07-31	Italy	38,067	15,817

RVcoll11H543	-	Lycaenidae	*Lycaena*	*phlaeas*	2011-06-08	Sicily	38,093	13,260
RVcoll11D032		Lycaenidae	*Lycaena*	*phlaeas*	2011-04-07	Sicily	38,139	13,126
RVcoll11I125		Lycaenidae	*Lycaena*	*phlaeas*	2011-06-19	Italy	38,221	16,183
RVcoll11D435		Lycaenidae	*Lycaena*	*phlaeas*	2011-04-14	Sicily	38,238	15,528
RVcoll11H820		Lycaenidae	*Lycaena*	*phlaeas*	2011-06-14	Vulcano	38,390	14,970
RVcoll11H823		Lycaenidae	*Lycaena*	*phlaeas*	2011-06-14	Vulcano	38,390	14,970
RVcoll11H826		Lycaenidae	*Lycaena*	*phlaeas*	2011-06-14	Vulcano	38,390	14,970
RVcoll12M299		Lycaenidae	*Lycaena*	*phlaeas*	2012-05-05	Lipari	38,485	14,918
RVcoll12M295		Lycaenidae	*Lycaena*	*phlaeas*	2012-05-05	Lipari	38,485	14,950
RVcoll12M297		Lycaenidae	*Lycaena*	*phlaeas*	2012-05-05	Lipari	38,485	14,950
RVcoll11H884		Lycaenidae	*Lycaena*	*phlaeas*	2011-06-15	Salina	38,560	14,830
RVcoll11H885		Lycaenidae	*Lycaena*	*phlaeas*	2011-06-15	Salina	38,560	14,840
RVcoll11H887		Lycaenidae	*Lycaena*	*phlaeas*	2011-06-15	Salina	38,560	14,840
RVcollLD2387		Lycaenidae	*Lycaena*	*phlaeas*	2010-06-30	Ustica	38,709	13,179
RVcollLD2388		Lycaenidae	*Lycaena*	*phlaeas*	2010-06-30	Ustica	38,709	13,179
RVcoll14A731		Lycaenidae	*Lycaena*	*phlaeas*	2013-08-21	Italy	39,217	16,140
LEPSS00054		Lycaenidae	*Lycaena*	*phlaeas*	2014-05-23	Italy	39,315	16,528
LEPSS00055		Lycaenidae	*Lycaena*	*phlaeas*	2014-05-23	Italy	39,315	16,528
LEPSS00198		Lycaenidae	*Lycaena*	*phlaeas*	2015-05-20	Italy	39,807	16,042
LEPSS00197		Lycaenidae	*Lycaena*	*phlaeas*	2015-05-20	Italy	39,828	16,077
RVcoll14L030	-	Nymphalidae	*Maniola*	*jurtina*	2007-05-22	Algeria	35,360	1,030
RVcoll11E144		Nymphalidae	*Maniola*	*jurtina*	2011-05-20	Maltese	36,042	14,274
RVcoll11E145		Nymphalidae	*Maniola*	*jurtina*	2011-05-20	Maltese	36,042	14,274
RVcoll11E146		Nymphalidae	*Maniola*	*jurtina*	2011-05-20	Maltese	36,042	14,274
RVcoll11E147		Nymphalidae	*Maniola*	*jurtina*	2011-05-20	Maltese	36,042	14,274
RVcoll13T315		Nymphalidae	*Maniola*	*jurtina*	2013-07-08	Tunisia	36,225	8,771
RVcoll14L151		Nymphalidae	*Maniola*	*jurtina*	2014-05-30	Algeria	36,305	5,221
RVcoll12N882		Nymphalidae	*Maniola*	*jurtina*	2012-05-26	Algeria	36,396	3,886
RVcoll12N892		Nymphalidae	*Maniola*	*jurtina*	2012-05-27	Algeria	36,442	4,207
RVcoll12N990		Nymphalidae	*Maniola*	*jurtina*	2012-07-12	Algeria	36,479	3,998
RVcoll12N992	KM033857	Nymphalidae	*Maniola*	*jurtina*	2012-07-12	Algeria	36,479	3,998
RVcoll14B082		Nymphalidae	*Maniola*	*jurtina*	2013-06-01	Algeria	36,621	5,816
RVcoll12N954		Nymphalidae	*Maniola*	*jurtina*	2012-06-29	Algeria	36,625	4,598
RVcoll08J938		Nymphalidae	*Maniola*	*jurtina*	2008-07-10	Tunisia	36,782	8,751
RVcoll13T321		Nymphalidae	*Maniola*	*jurtina*	2013-07-10	Tunisia	36,837	10,584
RVcollLD0092		Nymphalidae	*Maniola*	*jurtina*	2008-03-26	Tunisia	36,998	9,618
RVcollLD0093		Nymphalidae	*Maniola*	*jurtina*	2008-03-26	Tunisia	36,998	9,618
RVcoll12M355		Nymphalidae	*Maniola*	*jurtina*	2012-05-07	Sicily	37,068	14,350
RVcoll12M351		Nymphalidae	*Maniola*	*jurtina*	2012-05-07	Sicily	37,201	14,167
RVcoll12M394		Nymphalidae	*Maniola*	*jurtina*	2012-05-10	Sicily	37,493	13,684
RVcollLD2986	-	Nymphalidae	*Maniola*	*jurtina*	2010-06-30	Sicily	37,699	13,144
RVcoll11H761		Nymphalidae	*Maniola*	*jurtina*	2011-06-13	Sicily	37,850	14,710
RVcoll11I008		Nymphalidae	*Maniola*	*jurtina*	2011-06-17	Sicily	37,900	14,020
RVcoll12Q919		Nymphalidae	*Maniola*	*jurtina*	2012-07-31	Italy	38,067	15,817
RVcoll11H539		Nymphalidae	*Maniola*	*jurtina*	2011-06-08	Sicily	38,093	13,260
RVcoll11H566		Nymphalidae	*Maniola*	*jurtina*	2011-06-08	Sicily	38,097	13,240
RVcoll12R386		Nymphalidae	*Maniola*	*jurtina*	2012-06-26	Italy	38,120	15,670
RVcollLD2041		Nymphalidae	*Maniola*	*jurtina*	2010-06-26	Italy	38,150	15,796
RVcoll12R387		Nymphalidae	*Maniola*	*jurtina*	2012-06-26	Italy	38,150	15,810
RVcoll12Q595	KM033924	Nymphalidae	*Maniola*	*jurtina*	2012-08-01	Sicily	38,160	15,470
RVcoll12Q987		Nymphalidae	*Maniola*	*jurtina*	2012-08-01	Sicily	38,160	15,470
RVcoll12Q988		Nymphalidae	*Maniola*	*jurtina*	2012-08-01	Sicily	38,160	15,470
RVcoll12Q597		Nymphalidae	*Maniola*	*jurtina*	2012-08-01	Sicily	38,180	15,480
RVcoll12Q901		Nymphalidae	*Maniola*	*jurtina*	2012-07-31	Italy	38,217	15,883
RVcoll11H599		Nymphalidae	*Maniola*	*jurtina*	2011-06-08	Sicily	38,226	13,222
RVcoll11I171		Nymphalidae	*Maniola*	*jurtina*	2011-06-19	Italy	38,240	15,710
RVcoll11I172		Nymphalidae	*Maniola*	*jurtina*	2011-06-19	Italy	38,240	15,710
RVcoll11H837		Nymphalidae	*Maniola*	*jurtina*	2011-06-14	Vulcano	38,380	14,980
RVcoll11H834		Nymphalidae	*Maniola*	*jurtina*	2011-06-14	Vulcano	38,390	14,970
RVcoll11H836	KM020870	Nymphalidae	*Maniola*	*jurtina*	2011-06-14	Vulcano	38,390	14,970
RVcoll11H838		Nymphalidae	*Maniola*	*jurtina*	2011-06-14	Vulcano	38,390	14,970
RVcoll11H839		Nymphalidae	*Maniola*	*jurtina*	2011-06-14	Vulcano	38,390	14,970
RVcollLD1334		Nymphalidae	*Maniola*	*jurtina*	2009-06-30	Lipari	38,482	14,931
RVcollLD1335		Nymphalidae	*Maniola*	*jurtina*	2009-06-30	Lipari	38,482	14,931
RVcollLD1336		Nymphalidae	*Maniola*	*jurtina*	2009-06-30	Lipari	38,482	14,931
RVcollLD1337		Nymphalidae	*Maniola*	*jurtina*	2009-06-30	Lipari	38,482	14,931
RVcoll12M287		Nymphalidae	*Maniola*	*jurtina*	2012-05-05	Lipari	38,485	14,950
RVcoll11H868		Nymphalidae	*Maniola*	*jurtina*	2011-06-15	Salina	38,560	14,830
RVcoll14A758		Nymphalidae	*Maniola*	*jurtina*	2013-08-21	Italy	39,217	16,140
RVcoll14A697	-	Nymphalidae	*Maniola*	*jurtina*	2013-08-20	Italy	39,483	16,801
LEPSS00127		Nymphalidae	*Maniola*	*jurtina*	2014-05-20	Italy	39,560	16,751
LEPSS00126		Nymphalidae	*Maniola*	*jurtina*	2014-06-08	Italy	39,560	16,751
RVcoll11I264		Nymphalidae	*Maniola*	*jurtina*	2011-06-20	Italy	39,860	16,070
RVcoll11I269		Nymphalidae	*Maniola*	*jurtina*	2011-06-20	Italy	39,860	16,070
RVcoll12N402		Papilionidae	*Papilio*	*machaon*	2012-05-30	Tunisia	34,337	9,051
RVcoll12N336		Papilionidae	*Papilio*	*machaon*	2012-05-29	Tunisia	35,053	9,257
RVcoll12N337		Papilionidae	*Papilio*	*machaon*	2012-05-29	Tunisia	35,053	9,257
RVcoll14L054		Papilionidae	*Papilio*	*machaon*	2009-05-10	Algeria	35,280	0,920
RVcoll11H627		Papilionidae	*Papilio*	*machaon*	2011-06-09	Lampedusa	35,520	12,550
RVcoll11H628	-	Papilionidae	*Papilio*	*machaon*	2011-06-09	Lampedusa	35,520	12,550
RVcoll11H629		Papilionidae	*Papilio*	*machaon*	2011-06-09	Lampedusa	35,520	12,550
RVcoll11H630		Papilionidae	*Papilio*	*machaon*	2011-06-09	Lampedusa	35,520	12,550
RVcoll11J917		Papilionidae	*Papilio*	*machaon*	2008-05-21	Algeria	35,600	3,950
RVcoll14L131		Papilionidae	*Papilio*	*machaon*	2013-03-10	Algeria	35,750	4,550

RVcoll11E119		Papilionidae	*Papilio*	*machaon*	2011-05-20	Maltese	36,042	14,274
RVcoll11E120		Papilionidae	*Papilio*	*machaon*	2011-05-20	Maltese	36,042	14,274
RVcoll11E121		Papilionidae	*Papilio*	*machaon*	2011-05-20	Maltese	36,042	14,274
RVcoll11E122		Papilionidae	*Papilio*	*machaon*	2011-05-20	Maltese	36,042	14,274
RVcoll14L138		Papilionidae	*Papilio*	*machaon*	2014-04-18	Algeria	36,500	4,760
RVcoll11D403	-	Papilionidae	*Papilio*	*machaon*	2011-04-13	Sicily	37,158	15,049
RVcoll11D330		Papilionidae	*Papilio*	*machaon*	2011-04-13	Sicily	37,197	15,118
RVcoll11Y095		Papilionidae	*Papilio*	*machaon*	2011-06-29	Sicily	37,620	13,050
RVcoll12R119		Papilionidae	*Papilio*	*machaon*	2012-08-03	Sicily	37,840	14,060
RVcoll10C608		Papilionidae	*Papilio*	*machaon*	2010-06-26	Italy	38,122	15,675
RVcoll11D414		Papilionidae	*Papilio*	*machaon*	2011-04-14	Sicily	38,130	15,496
RVcoll14U815		Papilionidae	*Papilio*	*machaon*	2010-05-05	Italy	38,180	15,710
RVcoll11H602		Papilionidae	*Papilio*	*machaon*	2011-06-08	Sicily	38,226	13,222
RVcoll11H554		Papilionidae	*Papilio*	*machaon*	2011-06-08	Sicily	38,312	13,341
RVcoll11H791		Papilionidae	*Papilio*	*machaon*	2011-06-14	Vulcano	38,390	14,970
RVcoll11H792	-	Papilionidae	*Papilio*	*machaon*	2011-06-14	Vulcano	38,390	14,970
RVcoll12M286		Papilionidae	*Papilio*	*machaon*	2012-05-05	Lipari	38,485	14,950
RVcoll11H866		Papilionidae	*Papilio*	*machaon*	2011-06-15	Salina	38,560	14,830
RVcoll14N657		Papilionidae	*Papilio*	*machaon*	2014-10-01	Salina	38,570	14,830
RVcoll11D511		Papilionidae	*Papilio*	*machaon*	2011-04-18	Italy	38,674	15,926
RVcollLD2381		Papilionidae	*Papilio*	*machaon*	2010-06-30	Ustica	38,709	13,179
RVcoll11D492		Papilionidae	*Papilio*	*machaon*	2011-04-18	Italy	38,722	16,013
LEPSS00142		Papilionidae	*Papilio*	*machaon*	2014-08-03	Italy	39,282	16,207
LEPSS00141		Papilionidae	*Papilio*	*machaon*	2014-09-18	Italy	39,616	16,766
RVcoll12Q790		Papilionidae	*Papilio*	*machaon*	2012-07-30	Italy	39,930	16,170
RVcoll14O165	-	Papilionidae	*Papilio*	*saharae*	2004-05-12	Tunisia	34,420	8,190
RVcoll14O166		Papilionidae	*Papilio*	*saharae*	1997-10-07	Tunisia	35,420	9,850
RVcoll14O167		Papilionidae	*Papilio*	*saharae*	1997-11-13	Tunisia	35,420	9,850
RVcoll12N426		Nymphalidae	*Pararge*	*aegeria*	2012-05-31	Tunisia	35,199	8,674
RVcoll14L063		Nymphalidae	*Pararge*	*aegeria*	2014-05-15	Algeria	35,280	1,070
RVcoll12N953		Nymphalidae	*Pararge*	*aegeria*	2012-06-26	Algeria	35,742	4,549
RVcoll14L118		Nymphalidae	*Pararge*	*aegeria*	2007-05-22	Algeria	35,948	0,829
RVcoll12L284		Nymphalidae	*Pararge*	*aegeria*	2012-05-30	Maltese	36,040	14,200
RVcoll12L285		Nymphalidae	*Pararge*	*aegeria*	2012-05-30	Maltese	36,040	14,200
RVcoll12L291		Nymphalidae	*Pararge*	*aegeria*	2012-05-30	Maltese	36,042	14,274
RVcoll12L292		Nymphalidae	*Pararge*	*aegeria*	2012-05-30	Maltese	36,042	14,274
RVcoll12N290		Nymphalidae	*Pararge*	*aegeria*	2012-05-28	Tunisia	36,116	9,656
RVcoll12N291		Nymphalidae	*Pararge*	*aegeria*	2012-05-28	Tunisia	36,116	9,656
RVcoll12N463		Nymphalidae	*Pararge*	*aegeria*	2012-05-31	Tunisia	36,290	8,789
RVcoll12N895		Nymphalidae	*Pararge*	*aegeria*	2012-05-27	Algeria	36,442	4,207
RVcoll12N903		Nymphalidae	*Pararge*	*aegeria*	2012-05-27	Algeria	36,442	4,207
RVcoll12N993		Nymphalidae	*Pararge*	*aegeria*	2012-07-12	Algeria	36,479	3,998
RVcoll12N873		Nymphalidae	*Pararge*	*aegeria*	2012-05-20	Algeria	36,608	4,699
RVcoll12N947		Nymphalidae	*Pararge*	*aegeria*	2012-06-22	Algeria	36,763	5,102
RVcoll12N593		Nymphalidae	*Pararge*	*aegeria*	2012-05-25	Algeria	36,783	2,973
RVcoll12N549		Nymphalidae	*Pararge*	*aegeria*	2012-06-02	Tunisia	36,907	9,176
RVcoll13S935		Nymphalidae	*Pararge*	*aegeria*	2013-07-05	Tunisia	36,964	8,971
RVcoll11D400		Nymphalidae	*Pararge*	*aegeria*	2011-04-13	Sicily	37,158	15,049
RVcoll11Y102		Nymphalidae	*Pararge*	*aegeria*	2011-06-29	Sicily	37,620	13,050
RVcoll11D313		Nymphalidae	*Pararge*	*aegeria*	2011-04-12	Sicily	37,627	15,071
RVcoll11H532		Nymphalidae	*Pararge*	*aegeria*	2011-06-08	Sicily	38,093	13,260
RVcoll11H603		Nymphalidae	*Pararge*	*aegeria*	2011-06-08	Sicily	38,139	13,123
RVcoll11I139		Nymphalidae	*Pararge*	*aegeria*	2011-06-19	Italy	38,201	15,980
RVcoll11I138		Nymphalidae	*Pararge*	*aegeria*	2011-06-19	Italy	38,221	16,183
RVcoll11H604		Nymphalidae	*Pararge*	*aegeria*	2011-06-08	Sicily	38,226	13,222
RVcoll11D483	-	Nymphalidae	*Pararge*	*aegeria*	2011-04-16	Italy	38,233	15,717
RVcoll11D450		Nymphalidae	*Pararge*	*aegeria*	2011-04-14	Sicily	38,238	15,528
RVcoll11D453		Nymphalidae	*Pararge*	*aegeria*	2011-04-14	Sicily	38,238	15,528
RVcoll12M476		Nymphalidae	*Pararge*	*aegeria*	2012-05-12	Italy	38,250	15,951
RVcoll12M496		Nymphalidae	*Pararge*	*aegeria*	2012-05-12	Italy	38,383	16,167
RVcoll11H827		Nymphalidae	*Pararge*	*aegeria*	2011-06-14	Vulcano	38,390	14,970
RVcoll11H828		Nymphalidae	*Pararge*	*aegeria*	2011-06-14	Vulcano	38,390	14,970
RVcoll11H833		Nymphalidae	*Pararge*	*aegeria*	2011-06-14	Vulcano	38,390	14,970
RVcoll10C677		Nymphalidae	*Pararge*	*aegeria*	2010-06-30	Lipari	38,482	14,931
RVcoll12M292		Nymphalidae	*Pararge*	*aegeria*	2012-05-05	Lipari	38,485	14,950
RVcoll09T555	-	Nymphalidae	*Pararge*	*aegeria*	2009-07-01	Lipari	38,490	14,930
RVcoll11H895		Nymphalidae	*Pararge*	*aegeria*	2011-06-15	Salina	38,560	14,830
RVcoll11H897		Nymphalidae	*Pararge*	*aegeria*	2011-06-15	Salina	38,560	14,840
RVcoll11H900		Nymphalidae	*Pararge*	*aegeria*	2011-06-15	Salina	38,560	14,840
RVcoll14N731		Nymphalidae	*Pararge*	*aegeria*	2014-10-03	Stromboli	38,800	15,210
RVcoll14N732		Nymphalidae	*Pararge*	*aegeria*	2014-10-03	Stromboli	38,800	15,210
RVcollLD1664		Nymphalidae	*Pararge*	*aegeria*	2009-07-01	Stromboli	38,803	15,228
RVcollLD1665		Nymphalidae	*Pararge*	*aegeria*	2009-07-01	Stromboli	38,803	15,228
RVcoll14A714		Nymphalidae	*Pararge*	*aegeria*	2013-08-21	Italy	39,217	16,140
LEPSS00133		Nymphalidae	*Pararge*	*aegeria*	2014-06-22	Italy	39,392	16,530
LEPSS00134	-	Nymphalidae	*Pararge*	*aegeria*	2014-06-22	Italy	39,392	16,530
LEPSS00195		Nymphalidae	*Pararge*	*aegeria*	2015-05-20	Italy	39,828	16,077
RVcoll11I245		Nymphalidae	*Pararge*	*aegeria*	2011-06-20	Italy	39,860	16,070
RVcoll12N421		Pieridae	*Pieris*	*brassicae*	2012-05-31	Tunisia	35,199	8,674
RVcoll14L083		Pieridae	*Pieris*	*brassicae*	2009-05-10	Algeria	35,280	0,920
RVcoll14L087		Pieridae	*Pieris*	*brassicae*	2014-05-16	Algeria	35,280	1,070
RVcoll11H631		Pieridae	*Pieris*	*brassicae*	2011-06-09	Lampedusa	35,520	12,550
RVcoll11H632		Pieridae	*Pieris*	*brassicae*	2011-06-09	Lampedusa	35,520	12,550
RVcoll11H633		Pieridae	*Pieris*	*brassicae*	2011-06-09	Lampedusa	35,520	12,550

RVcoll11H635		Pieridae	*Pieris*	*brassicae*	2011-06-09	Lampedusa	35,520	12,550
RVcoll12N555		Pieridae	*Pieris*	*brassicae*	2012-05-02	Algeria	35,742	4,550
RVcoll14L141		Pieridae	*Pieris*	*brassicae*	2014-04-20	Algeria	35,850	4,930
RVcoll10C582		Pieridae	*Pieris*	*brassicae*	2010-06-17	Maltese	35,850	14,390
RVcollLD2731		Pieridae	*Pieris*	*brassicae*	2010-06-17	Maltese	35,858	14,399
RVcoll12N630		Pieridae	*Pieris*	*brassicae*	2012-05-13	Algeria	36,573	4,538
RVcoll12N632		Pieridae	*Pieris*	*brassicae*	2012-05-16	Algeria	36,592	4,611
RVcoll12N475		Pieridae	*Pieris*	*brassicae*	2012-06-01	Tunisia	36,731	8,710
RVcoll11D141		Pieridae	*Pieris*	*brassicae*	2011-04-09	Pantelleria	36,761	11,998
RVcoll11D144		Pieridae	*Pieris*	*brassicae*	2011-04-09	Pantelleria	36,761	11,998
RVcoll11D118		Pieridae	*Pieris*	*brassicae*	2011-04-08	Pantelleria	36,785	11,981
RVcoll12M358	-	Pieridae	*Pieris*	*brassicae*	2012-05-07	Sicily	37,068	14,350
RVcoll11D372		Pieridae	*Pieris*	*brassicae*	2011-04-13	Sicily	37,197	15,118
RVcoll11D212		Pieridae	*Pieris*	*brassicae*	2011-04-11	Sicily	37,838	13,429
RVcoll11H732		Pieridae	*Pieris*	*brassicae*	2011-06-13	Sicily	37,920	14,660
RVcoll11D163		Pieridae	*Pieris*	*brassicae*	2011-04-10	Marettimo	37,961	12,067
RVcoll11D164		Pieridae	*Pieris*	*brassicae*	2011-04-10	Marettimo	37,961	12,067
RVcoll11D168		Pieridae	*Pieris*	*brassicae*	2011-04-10	Marettimo	37,961	12,067
RVcoll12R017		Pieridae	*Pieris*	*brassicae*	2012-08-02	Sicily	37,970	14,770
RVcoll11H540		Pieridae	*Pieris*	*brassicae*	2011-06-08	Sicily	38,093	13,260
RVcoll12Q914		Pieridae	*Pieris*	*brassicae*	2012-07-31	Italy	38,100	15,833
RVcollLD2836	-	Pieridae	*Pieris*	*brassicae*	2010-06-27	Sicily	38,208	15,499
RVcoll11I140		Pieridae	*Pieris*	*brassicae*	2011-06-19	Italy	38,221	16,183
RVcoll12Q889		Pieridae	*Pieris*	*brassicae*	2012-07-31	Italy	38,250	15,950
RVcoll11H796		Pieridae	*Pieris*	*brassicae*	2011-06-14	Vulcano	38,390	14,970
RVcoll11H798		Pieridae	*Pieris*	*brassicae*	2011-06-14	Vulcano	38,390	14,970
RVcoll10C680		Pieridae	*Pieris*	*brassicae*	2010-06-30	Lipari	38,482	14,931
RVcoll11H878		Pieridae	*Pieris*	*brassicae*	2011-06-15	Salina	38,560	14,830
RVcoll11H876		Pieridae	*Pieris*	*brassicae*	2011-06-15	Salina	38,560	14,840
RVcoll11H877		Pieridae	*Pieris*	*brassicae*	2011-06-15	Salina	38,560	14,840
RVcollLD2382		Pieridae	*Pieris*	*brassicae*	2010-06-30	Ustica	38,709	13,179
RVcoll10C688	-	Pieridae	*Pieris*	*brassicae*	2012-07-01	Stromboli	38,803	15,228
RVcoll14A723		Pieridae	*Pieris*	*brassicae*	2013-08-21	Italy	39,217	16,140
LEPSS00022		Pieridae	*Pieris*	*brassicae*	2014-05-23	Italy	39,315	16,528
LEPSS00021		Pieridae	*Pieris*	*brassicae*	2014-05-20	Italy	39,560	16,751
RVcoll11I190		Pieridae	*Pieris*	*brassicae*	2011-06-20	Italy	39,930	16,150
RVcoll11J726		Pieridae	*Pieris*	*mannii*	2011-07-22	Sicily	37,850	13,430
RVcollLD1494		Pieridae	*Pieris*	*mannii*	2008-05-26	Sicily	37,868	13,384
RVcoll11I003		Pieridae	*Pieris*	*mannii*	2011-06-17	Sicily	37,900	14,020
RVcollLD1752		Pieridae	*Pieris*	*mannii*	2010-04-03	Sicily	38,003	14,729
RVcoll11H697		Pieridae	*Pieris*	*mannii*	2011-06-12	Levanzo	38,010	12,330
RVcoll10C671	-	Pieridae	*Pieris*	*mannii*	2010-06-29	Italy	38,084	15,834
RVcoll10C672		Pieridae	*Pieris*	*mannii*	2010-06-29	Italy	38,084	15,834
RVcoll11H795		Pieridae	*Pieris*	*mannii*	2011-06-14	Vulcano	38,390	14,970
RVcoll12M302		Pieridae	*Pieris*	*mannii*	2012-05-05	Lipari	38,485	14,918
RVcoll12M300		Pieridae	*Pieris*	*mannii*	2012-05-05	Lipari	38,485	14,950
RVcoll12M301		Pieridae	*Pieris*	*mannii*	2012-05-05	Lipari	38,485	14,950
RVcoll11H874		Pieridae	*Pieris*	*mannii*	2011-06-15	Salina	38,560	14,830
RVcoll11H875		Pieridae	*Pieris*	*mannii*	2011-06-15	Salina	38,560	14,840
RVcoll14A722		Pieridae	*Pieris*	*mannii*	2013-08-21	Italy	39,217	16,140
RVcoll14A724		Pieridae	*Pieris*	*mannii*	2013-08-21	Italy	39,217	16,140
LEPSS00250		Pieridae	*Pieris*	*mannii*	2015-07-05	Italy	39,291	16,462
RVcoll15A805	-	Pieridae	*Pieris*	*mannii*	2015-06-04	Italy	39,814	16,309
RVcoll12Q811		Pieridae	*Pieris*	*mannii*	2012-07-30	Italy	39,930	16,170
RVcoll14B039		Pieridae	*Pieris*	*rapae*	2013-03-07	Algeria	33,104	6,088
RVcoll12N391		Pieridae	*Pieris*	*rapae*	2012-05-30	Tunisia	33,407	9,008
RVcoll12N386		Pieridae	*Pieris*	*rapae*	2012-05-30	Tunisia	33,942	8,173
RVcoll12N370		Pieridae	*Pieris*	*rapae*	2012-05-29	Tunisia	34,345	8,329
RVcoll12N333		Pieridae	*Pieris*	*rapae*	2012-05-29	Tunisia	35,053	9,257
RVcoll14L040		Pieridae	*Pieris*	*rapae*	2009-05-28	Algeria	35,190	1,130
RVcoll08J908		Pieridae	*Pieris*	*rapae*	2008-07-08	Tunisia	35,200	8,674
RVcoll11J930		Pieridae	*Pieris*	*rapae*	2011-12-02	Algeria	35,220	4,190
RVcoll14L068	-	Pieridae	*Pieris*	*rapae*	2009-05-10	Algeria	35,280	0,920
RVcoll12N324		Pieridae	*Pieris*	*rapae*	2012-05-29	Tunisia	35,425	9,571
RVcoll11H617		Pieridae	*Pieris*	*rapae*	2011-06-09	Lampedusa	35,520	12,550
RVcoll11H618		Pieridae	*Pieris*	*rapae*	2011-06-09	Lampedusa	35,520	12,550
RVcoll11H619		Pieridae	*Pieris*	*rapae*	2011-06-09	Lampedusa	35,520	12,550
RVcoll11J918		Pieridae	*Pieris*	*rapae*	2011-11-10	Algeria	35,710	4,520
RVcoll11J921		Pieridae	*Pieris*	*rapae*	2011-11-10	Algeria	35,710	4,520
RVcoll08J919		Pieridae	*Pieris*	*rapae*	2008-07-09	Tunisia	35,749	8,361
RVcoll14L137		Pieridae	*Pieris*	*rapae*	2013-03-10	Algeria	35,750	4,550
RVcoll11E105		Pieridae	*Pieris*	*rapae*	2011-05-19	Maltese	35,859	14,400
RVcoll11E108		Pieridae	*Pieris*	*rapae*	2011-05-19	Maltese	35,859	14,400
RVcoll10C600		Pieridae	*Pieris*	*rapae*	2010-06-17	Maltese	35,902	14,407
RVcoll11E138		Pieridae	*Pieris*	*rapae*	2011-05-20	Maltese	36,042	14,274
RVcoll11E139		Pieridae	*Pieris*	*rapae*	2011-05-20	Maltese	36,042	14,274
RVcoll11E140		Pieridae	*Pieris*	*rapae*	2011-05-20	Maltese	36,042	14,274
RVcoll11E141		Pieridae	*Pieris*	*rapae*	2011-05-20	Maltese	36,042	14,274
RVcoll12N606		Pieridae	*Pieris*	*rapae*	2012-05-11	Algeria	36,329	5,360
RVcoll12N237		Pieridae	*Pieris*	*rapae*	2012-05-28	Tunisia	36,415	9,916
RVcoll12N886		Pieridae	*Pieris*	*rapae*	2012-05-27	Algeria	36,446	4,113
RVcoll12N989		Pieridae	*Pieris*	*rapae*	2012-07-12	Algeria	36,479	3,998
RVcoll12N934		Pieridae	*Pieris*	*rapae*	2012-06-16	Algeria	36,630	4,598
RVcoll12N209		Pieridae	*Pieris*	*rapae*	2012-05-28	Tunisia	36,651	10,161

RVcoll11D146		Pieridae	*Pieris*	*rapae*	2011-04-09	Pantelleria	36,761	11,998
RVcoll08J939		Pieridae	*Pieris*	*rapae*	2008-07-10	Tunisia	36,782	8,751
RVcoll11D137		Pieridae	*Pieris*	*rapae*	2011-04-09	Pantelleria	36,785	11,981
RVcoll11D154		Pieridae	*Pieris*	*rapae*	2011-04-09	Pantelleria	36,819	12,006
RVcoll12N550		Pieridae	*Pieris*	*rapae*	2012-06-02	Tunisia	36,907	9,176
RVcoll13S914		Pieridae	*Pieris*	*rapae*	2013-07-05	Tunisia	37,010	9,625
RVcoll12M357		Pieridae	*Pieris*	*rapae*	2012-05-07	Sicily	37,068	14,350
RVcoll13T372		Pieridae	*Pieris*	*rapae*	2013-07-04	Tunisia	37,164	10,110
RVcoll11D376		Pieridae	*Pieris*	*rapae*	2011-04-13	Sicily	37,197	15,118
RVcoll12M383	-	Pieridae	*Pieris*	*rapae*	2012-05-10	Sicily	37,493	13,684
RVcoll11Y106		Pieridae	*Pieris*	*rapae*	2011-06-29	Sicily	37,620	13,050
RVcoll11H747		Pieridae	*Pieris*	*rapae*	2011-06-13	Sicily	37,850	14,710
RVcoll11I023		Pieridae	*Pieris*	*rapae*	2011-06-17	Sicily	37,900	14,020
RVcoll11D162		Pieridae	*Pieris*	*rapae*	2011-04-10	Marettimo	37,961	12,067
RVcoll12R019		Pieridae	*Pieris*	*rapae*	2012-08-02	Sicily	37,970	14,770
RVcoll11H698		Pieridae	*Pieris*	*rapae*	2011-06-12	Levanzo	38,000	12,330
RVcoll12M266		Pieridae	*Pieris*	*rapae*	2012-05-04	Italy	38,000	15,801
RVcoll11H699		Pieridae	*Pieris*	*rapae*	2011-06-12	Levanzo	38,010	12,330
RVcoll11H703		Pieridae	*Pieris*	*rapae*	2011-06-12	Levanzo	38,010	12,330
RVcoll11H541		Pieridae	*Pieris*	*rapae*	2011-06-08	Sicily	38,093	13,260
RVcoll12Q902	-	Pieridae	*Pieris*	*rapae*	2012-07-31	Italy	38,217	15,883
RVcoll11H597		Pieridae	*Pieris*	*rapae*	2011-06-08	Sicily	38,226	13,222
RVcoll11D439		Pieridae	*Pieris*	*rapae*	2011-04-14	Sicily	38,238	15,528
RVcoll11H799		Pieridae	*Pieris*	*rapae*	2011-06-14	Vulcano	38,390	14,970
RVcoll11H800		Pieridae	*Pieris*	*rapae*	2011-06-14	Vulcano	38,390	14,970
RVcoll11H803		Pieridae	*Pieris*	*rapae*	2011-06-14	Vulcano	38,390	14,970
RVcoll11H804		Pieridae	*Pieris*	*rapae*	2011-06-14	Vulcano	38,390	14,970
RVcoll12M307		Pieridae	*Pieris*	*rapae*	2012-05-05	Lipari	38,485	14,918
RVcoll12M305		Pieridae	*Pieris*	*rapae*	2012-05-05	Lipari	38,485	14,950
RVcoll12M306		Pieridae	*Pieris*	*rapae*	2012-05-05	Lipari	38,485	14,950
RVcoll11H883	-	Pieridae	*Pieris*	*rapae*	2011-06-15	Salina	38,560	14,830
RVcoll11H880		Pieridae	*Pieris*	*rapae*	2011-06-15	Salina	38,560	14,840
RVcoll11H882		Pieridae	*Pieris*	*rapae*	2011-06-15	Salina	38,560	14,840
RVcollLD2386		Pieridae	*Pieris*	*rapae*	2010-06-30	Ustica	38,709	13,179
RVcoll11D505		Pieridae	*Pieris*	*rapae*	2011-04-18	Italy	38,722	16,013
RVcoll14N711		Pieridae	*Pieris*	*rapae*	2014-10-03	Stromboli	38,800	15,210
RVcoll14N712		Pieridae	*Pieris*	*rapae*	2014-10-03	Stromboli	38,800	15,210
RVcoll10C687		Pieridae	*Pieris*	*rapae*	2012-07-01	Stromboli	38,803	15,228
LEPSS00024		Pieridae	*Pieris*	*rapae*	2014-05-23	Italy	39,315	16,528
LEPSS00023		Pieridae	*Pieris*	*rapae*	2014-05-20	Italy	39,443	16,604
RVcoll12Q812		Pieridae	*Pieris*	*rapae*	2012-07-30	Italy	39,930	16,170
RVcoll12Q813	-	Pieridae	*Pieris*	*rapae*	2012-07-30	Italy	39,930	16,170
RVcoll12N400		Lycaenidae	*Polyommatus*	*celina*	2012-05-30	Tunisia	34,337	9,051
RVcoll14L031		Lycaenidae	*Polyommatus*	*celina*	2007-05-23	Algeria	35,280	0,920
RVcoll12N574		Lycaenidae	*Polyommatus*	*celina*	2012-05-18	Algeria	35,326	4,297
RVcoll13T573		Lycaenidae	*Polyommatus*	*celina*	2013-07-09	Tunisia	35,447	9,224
RVcoll08J900		Lycaenidae	*Polyommatus*	*celina*	2008-07-06	Tunisia	35,460	10,605
RVcoll11H644		Lycaenidae	*Polyommatus*	*celina*	2011-06-09	Lampedusa	35,520	12,560
RVcoll11H645		Lycaenidae	*Polyommatus*	*celina*	2011-06-09	Lampedusa	35,520	12,560
RVcoll11H646		Lycaenidae	*Polyommatus*	*celina*	2011-06-09	Lampedusa	35,520	12,560
RVcoll11H647	KM459259	Lycaenidae	*Polyommatus*	*celina*	2011-06-09	Lampedusa	35,520	12,560
RVcoll11H648		Lycaenidae	*Polyommatus*	*celina*	2011-06-09	Lampedusa	35,520	12,560
RVcoll11H649		Lycaenidae	*Polyommatus*	*celina*	2011-06-09	Lampedusa	35,520	12,560
RVcoll11H652		Lycaenidae	*Polyommatus*	*celina*	2011-06-09	Lampedusa	35,520	12,560
RVcoll08J937		Lycaenidae	*Polyommatus*	*celina*	2008-07-09	Tunisia	35,745	8,384
RVcoll08J920		Lycaenidae	*Polyommatus*	*celina*	2008-07-09	Tunisia	35,750	8,361
RVcoll08J926		Lycaenidae	*Polyommatus*	*celina*	2008-07-09	Tunisia	35,750	8,361
RVcoll11E102		Lycaenidae	*Polyommatus*	*celina*	2011-05-19	Maltese	35,859	14,400
RVcollLD2328		Lycaenidae	*Polyommatus*	*celina*	2010-06-15	Maltese	35,891	14,390
RVcollLD2329		Lycaenidae	*Polyommatus*	*celina*	2010-06-15	Maltese	35,891	14,390
RVcollLD2330	KM459307	Lycaenidae	*Polyommatus*	*celina*	2010-06-15	Maltese	35,891	14,390
RVcollLD2331		Lycaenidae	*Polyommatus*	*celina*	2010-06-15	Maltese	35,891	14,390
RVcollLD2728		Lycaenidae	*Polyommatus*	*celina*	2010-06-17	Maltese	35,902	14,407
RVcollLD2729		Lycaenidae	*Polyommatus*	*celina*	2010-06-17	Maltese	35,902	14,407
RVcollLD2730		Lycaenidae	*Polyommatus*	*celina*	2010-06-17	Maltese	35,902	14,407
RVcollLD2717		Lycaenidae	*Polyommatus*	*celina*	2010-06-17	Maltese	35,927	14,442
RVcoll11E113		Lycaenidae	*Polyommatus*	*celina*	2011-05-20	Maltese	36,042	14,274
RVcoll11E114		Lycaenidae	*Polyommatus*	*celina*	2011-05-20	Maltese	36,042	14,274
RVcoll11E115		Lycaenidae	*Polyommatus*	*celina*	2011-05-20	Maltese	36,042	14,274
RVcoll11E116		Lycaenidae	*Polyommatus*	*celina*	2011-05-20	Maltese	36,042	14,274
RVcoll11E117	KM459238	Lycaenidae	*Polyommatus*	*celina*	2011-05-20	Maltese	36,042	14,274
RVcoll11E118		Lycaenidae	*Polyommatus*	*celina*	2011-05-20	Maltese	36,042	14,274
RVcoll12N603		Lycaenidae	*Polyommatus*	*celina*	2012-05-11	Algeria	36,329	5,360
RVcoll12N876		Lycaenidae	*Polyommatus*	*celina*	2012-05-24	Algeria	36,612	4,635
RVcoll12N204		Lycaenidae	*Polyommatus*	*celina*	2012-05-28	Tunisia	36,651	10,161
RVcoll08J940		Lycaenidae	*Polyommatus*	*celina*	2008-07-10	Tunisia	36,782	8,751
RVcoll08J945		Lycaenidae	*Polyommatus*	*celina*	2008-07-10	Tunisia	36,782	8,751
RVcoll11D133		Lycaenidae	*Polyommatus*	*celina*	2011-04-09	Pantelleria	36,782	12,000
RVcoll11H678		Lycaenidae	*Polyommatus*	*celina*	2011-06-11	Pantelleria	36,818	11,952
RVcoll11H679	KM459264	Lycaenidae	*Polyommatus*	*celina*	2011-06-11	Pantelleria	36,818	11,952
RVcoll11H680		Lycaenidae	*Polyommatus*	*celina*	2011-06-11	Pantelleria	36,818	11,952
RVcoll11H683		Lycaenidae	*Polyommatus*	*celina*	2011-06-11	Pantelleria	36,818	11,952
RVcoll11H685		Lycaenidae	*Polyommatus*	*celina*	2011-06-11	Pantelleria	36,818	11,952
RVcoll12N542		Lycaenidae	*Polyommatus*	*celina*	2012-06-02	Tunisia	36,992	9,078

RVcoll12N543		Lycaenidae	*Polyommatus*	*celina*	2012-06-02	Tunisia	36,992	9,078
RVcollLD0163		Lycaenidae	*Polyommatus*	*celina*	2008-04-26	Tunisia	36,998	9,618
RVcoll08R542		Lycaenidae	*Polyommatus*	*celina*	2011-04-13	Sicily	37,158	15,050
RVcoll13T370		Lycaenidae	*Polyommatus*	*celina*	2013-07-04	Tunisia	37,164	10,110
RVcoll13T395		Lycaenidae	*Polyommatus*	*celina*	2013-07-05	Tunisia	37,313	9,856
RVcoll12M386	-	Lycaenidae	*Polyommatus*	*celina*	2012-05-10	Sicily	37,493	13,684
RVcollLD2323		Lycaenidae	*Polyommatus*	*celina*	2010-06-15	Sicily	37,620	13,060
RVcoll12Q582		Lycaenidae	*Polyommatus*	*celina*	2012-08-01	Sicily	37,800	15,040
RVcoll11I052		Lycaenidae	*Polyommatus*	*celina*	2011-06-18	Sicily	37,810	15,070
RVcoll11D263		Lycaenidae	*Polyommatus*	*celina*	2011-04-11	Sicily	37,838	13,429
RVcoll11I086		Lycaenidae	*Polyommatus*	*celina*	2011-06-18	Sicily	37,840	14,989
RVcoll09T512		Lycaenidae	*Polyommatus*	*celina*	2008-05-14	Sicily	37,868	13,384
RVcoll09T513		Lycaenidae	*Polyommatus*	*celina*	2008-05-14	Sicily	37,868	13,384
RVcoll11H958		Lycaenidae	*Polyommatus*	*celina*	2011-06-16	Sicily	37,880	14,030
RVcoll11H725		Lycaenidae	*Polyommatus*	*celina*	2011-06-12	Levanzo	38,000	12,330
RVcoll11H727		Lycaenidae	*Polyommatus*	*celina*	2011-06-12	Levanzo	38,000	12,330
RVcoll11H728		Lycaenidae	*Polyommatus*	*celina*	2011-06-12	Levanzo	38,000	12,330
RVcoll11H729		Lycaenidae	*Polyommatus*	*celina*	2011-06-12	Levanzo	38,000	12,330
RVcoll11H730		Lycaenidae	*Polyommatus*	*celina*	2011-06-12	Levanzo	38,000	12,330
RVcoll11H550		Lycaenidae	*Polyommatus*	*celina*	2011-06-08	Sicily	38,082	13,256
RVcollLD0183		Lycaenidae	*Polyommatus*	*celina*	-	Sicily	38,090	15,300
RVcollLD0186		Lycaenidae	*Polyommatus*	*celina*	-	Sicily	38,090	15,300
RVcoll11H596		Lycaenidae	*Polyommatus*	*celina*	2011-06-08	Sicily	38,120	13,129
RVcoll08R548		Lycaenidae	*Polyommatus*	*celina*	2011-04-14	Sicily	38,130	15,496
RVcoll11D411	KM459230	Lycaenidae	*Polyommatus*	*celina*	2011-04-14	Sicily	38,130	15,496
RVcoll08R500		Lycaenidae	*Polyommatus*	*celina*	2011-04-07	Sicily	38,139	13,126
RVcollLD2345		Lycaenidae	*Polyommatus*	*celina*	2010-06-15	Sicily	38,170	15,470
RVcollLD2325		Lycaenidae	*Polyommatus*	*celina*	2010-06-15	Sicily	38,260	15,590
RVcoll08R549		Lycaenidae	*Polyommatus*	*celina*	2011-04-14	Sicily	38,276	15,590
RVcoll11D473		Lycaenidae	*Polyommatus*	*celina*	2011-04-14	Sicily	38,276	15,590
RVcoll11H846		Lycaenidae	*Polyommatus*	*celina*	2011-06-14	Vulcano	38,380	14,980
RVcoll11H847		Lycaenidae	*Polyommatus*	*celina*	2011-06-14	Vulcano	38,380	14,980
RVcoll11H848		Lycaenidae	*Polyommatus*	*celina*	2011-06-14	Vulcano	38,380	14,980
RVcoll11H849		Lycaenidae	*Polyommatus*	*celina*	2011-06-14	Vulcano	38,380	14,980
RVcollLD1571	JN084677	Lycaenidae	*Polyommatus*	*celina*	2009-06-30	Lipari	38,482	14,931
RVcollLD1572		Lycaenidae	*Polyommatus*	*celina*	2009-06-30	Lipari	38,482	14,931
RVcoll09T550		Lycaenidae	*Polyommatus*	*celina*	2009-07-01	Lipari	38,490	14,930
RVcoll09T551		Lycaenidae	*Polyommatus*	*celina*	2009-07-01	Lipari	38,496	14,933
RVcoll09T552		Lycaenidae	*Polyommatus*	*celina*	2009-07-01	Lipari	38,496	14,933
RVcoll11H902		Lycaenidae	*Polyommatus*	*celina*	2011-06-15	Salina	38,560	14,830
RVcoll11H903		Lycaenidae	*Polyommatus*	*celina*	2011-06-15	Salina	38,560	14,830
RVcoll11H904		Lycaenidae	*Polyommatus*	*celina*	2011-06-15	Salina	38,560	14,830
RVcoll11H905		Lycaenidae	*Polyommatus*	*celina*	2011-06-15	Salina	38,560	14,830
RVcoll11H917		Lycaenidae	*Polyommatus*	*celina*	2011-06-15	Salina	38,560	14,830
RVcollLD2326		Lycaenidae	*Polyommatus*	*celina*	2010-06-30	Ustica	38,709	13,179
RVcollLD2327		Lycaenidae	*Polyommatus*	*celina*	2010-06-30	Ustica	38,709	13,179
RVcollLD2396		Lycaenidae	*Polyommatus*	*celina*	2010-06-30	Ustica	38,709	13,179
RVcollLD2397		Lycaenidae	*Polyommatus*	*celina*	2010-06-30	Ustica	38,709	13,179
RVcoll12N393		Pieridae	*Pontia*	*daplidice*	2012-05-30	Tunisia	33,407	9,008
RVcoll12N410		Pieridae	*Pontia*	*daplidice*	2012-05-30	Tunisia	34,337	9,051
RVcoll12N374		Pieridae	*Pontia*	*daplidice*	2012-05-29	Tunisia	34,345	8,329
RVcoll14L055		Pieridae	*Pontia*	*daplidice*	2009-05-13	Algeria	35,120	0,830
RVcoll11J929		Pieridae	*Pontia*	*daplidice*	2011-12-02	Algeria	35,220	4,190
RVcoll11J931		Pieridae	*Pontia*	*daplidice*	2011-12-02	Algeria	35,220	4,190
RVcoll12N329		Pieridae	*Pontia*	*daplidice*	2012-05-29	Tunisia	35,425	9,571
RVcoll08J901		Pieridae	*Pontia*	*daplidice*	2008-07-06	Tunisia	35,460	10,605
RVcoll11H636		Pieridae	*Pontia*	*daplidice*	2011-06-09	Lampedusa	35,520	12,550
RVcoll11H637		Pieridae	*Pontia*	*daplidice*	2011-06-09	Lampedusa	35,520	12,550
RVcoll11H638		Pieridae	*Pontia*	*daplidice*	2011-06-09	Lampedusa	35,520	12,550
RVcoll11H642		Pieridae	*Pontia*	*daplidice*	2011-06-09	Lampedusa	35,520	12,550
RVcoll11J919		Pieridae	*Pontia*	*daplidice*	2011-11-10	Algeria	35,710	4,520
RVcoll11J920		Pieridae	*Pontia*	*daplidice*	2011-11-10	Algeria	35,710	4,520
RVcoll08J912		Pieridae	*Pontia*	*daplidice*	2008-07-08	Tunisia	35,858	8,493
RVcoll14B092		Pieridae	*Pontia*	*daplidice*	2013-10-17	Algeria	36,001	4,298
RVcoll13T495		Pieridae	*Pontia*	*daplidice*	2013-07-08	Tunisia	36,225	8,771
RVcoll13T587		Pieridae	*Pontia*	*daplidice*	2013-07-10	Tunisia	36,275	10,275
RVcoll12N257		Pieridae	*Pontia*	*daplidice*	2012-05-28	Tunisia	36,294	9,811
RVcoll13S964		Pieridae	*Pontia*	*daplidice*	2013-07-06	Tunisia	36,741	8,680
RVcoll10C453		Pieridae	*Pontia*	*edusa*	2010-06-15	Maltese	35,880	14,400
RVcoll10C594		Pieridae	*Pontia*	*edusa*	2010-06-17	Maltese	35,927	14,442
RVcoll10C595		Pieridae	*Pontia*	*edusa*	2010-06-17	Maltese	35,927	14,442
RVcollLD2719		Pieridae	*Pontia*	*edusa*	2010-06-17	Maltese	35,927	14,442
RVcoll12M354		Pieridae	*Pontia*	*edusa*	2012-05-07	Sicily	37,068	14,350
RVcoll11D369		Pieridae	*Pontia*	*edusa*	2011-04-13	Sicily	37,197	15,118
RVcoll12M379	-	Pieridae	*Pontia*	*edusa*	2012-05-10	Sicily	37,493	13,684
RVcoll11Y096		Pieridae	*Pontia*	*edusa*	2011-06-29	Sicily	37,620	13,050
RVcoll12R182		Pieridae	*Pontia*	*edusa*	2012-08-04	Sicily	37,840	13,420
RVcoll12Q939		Pieridae	*Pontia*	*edusa*	2012-08-01	Sicily	37,840	14,989
RVcoll11H688		Pieridae	*Pontia*	*edusa*	2011-06-12	Levanzo	38,000	12,330
RVcoll11H689		Pieridae	*Pontia*	*edusa*	2011-06-12	Levanzo	38,000	12,330
RVcoll11H690		Pieridae	*Pontia*	*edusa*	2011-06-12	Levanzo	38,010	12,330
RVcoll10C616		Pieridae	*Pontia*	*edusa*	2010-06-26	Italy	38,122	15,675
RVcoll11D461		Pieridae	*Pontia*	*edusa*	2011-04-14	Sicily	38,241	15,562
RVcoll12Q884		Pieridae	*Pontia*	*edusa*	2012-07-31	Italy	38,250	15,950

RVcoll12Q897		Pieridae	*Pontia*	*edusa*	2012-07-31	Italy	38,250	15,950
RVcoll11H598		Pieridae	*Pontia*	*edusa*	2011-06-08	Sicily	38,312	13,341
RVcoll11H810		Pieridae	*Pontia*	*edusa*	2011-06-14	Vulcano	38,390	14,970
RVcoll11H813		Pieridae	*Pontia*	*edusa*	2011-06-14	Vulcano	38,390	14,970
RVcollLD2571		Pieridae	*Pontia*	*edusa*	2009-06-30	Lipari	38,482	14,931
RVcollLD2572		Pieridae	*Pontia*	*edusa*	2009-06-30	Lipari	38,482	14,931
RVcoll10C675		Pieridae	*Pontia*	*edusa*	2010-06-30	Lipari	38,482	14,931
RVcoll14W000		Pieridae	*Pontia*	*edusa*	2014-06-21	Ustica	38,709	13,179
RVcollLD2573		Pieridae	*Pontia*	*edusa*	2009-07-01	Stromboli	38,803	15,228
LEPSS00030		Pieridae	*Pontia*	*edusa*	2014-07-08	Italy	39,323	16,475
LEPSS00029	-	Pieridae	*Pontia*	*edusa*	2014-05-20	Italy	39,560	16,751
RVcoll14A703		Pieridae	*Pontia*	*edusa*	2013-08-20	Italy	39,720	16,525
RVcoll15A807		Pieridae	*Pontia*	*edusa*	2015-06-04	Italy	39,840	16,258
RVcoll11D210		Lycaenidae	*Pseudophilotes*	*baton*	2011-04-11	Sicily	37,838	13,429
RVcoll12M420		Lycaenidae	*Pseudophilotes*	*baton*	2012-05-10	Sicily	37,840	13,424
RVcollLD3105		Lycaenidae	*Pseudophilotes*	*baton*	2010-06-28	Sicily	37,880	14,030
RVcoll12M586		Lycaenidae	*Pseudophilotes*	*baton*	2012-05-06	Sicily	37,901	14,952
RVcoll11H787		Lycaenidae	*Pseudophilotes*	*baton*	2011-06-14	Vulcano	38,390	14,970
RVcoll11H788		Lycaenidae	*Pseudophilotes*	*baton*	2011-06-14	Vulcano	38,390	14,970
LEPSS00232		Lycaenidae	*Pseudophilotes*	*baton*	2015-06-22	Italy	38,535	16,304
LEPSS00051	-	Lycaenidae	*Pseudophilotes*	*baton*	2014-04-24	Italy	39,172	16,352
RVcoll14A094		Lycaenidae	*Pseudophilotes*	*baton*	2013-08-21	Italy	39,217	16,140
LEPSS00100		Lycaenidae	*Pseudophilotes*	*baton*	2014-08-20	Italy	39,395	16,599
RVcoll07E010		Lycaenidae	*Pseudophilotes*	*baton*	2007-07-10	Italy	39,903	16,140
RVcoll12N320		Nymphalidae	*Pyronia*	*cecilia*	2012-05-29	Tunisia	35,425	9,571
RVcoll13T559		Nymphalidae	*Pyronia*	*cecilia*	2013-07-08	Tunisia	35,523	8,642
RVcoll08J925		Nymphalidae	*Pyronia*	*cecilia*	2008-07-09	Tunisia	35,750	8,361
RVcoll12N454		Nymphalidae	*Pyronia*	*cecilia*	2012-05-31	Tunisia	36,290	8,789
RVcoll12N260		Nymphalidae	*Pyronia*	*cecilia*	2012-05-28	Tunisia	36,294	9,811
RVcoll13T602		Nymphalidae	*Pyronia*	*cecilia*	2013-07-10	Tunisia	36,333	10,093
RVcoll12N235	-	Nymphalidae	*Pyronia*	*cecilia*	2012-05-28	Tunisia	36,415	9,916
RVcoll12N999		Nymphalidae	*Pyronia*	*cecilia*	2012-07-13	Algeria	36,457	4,107
RVcoll12N217		Nymphalidae	*Pyronia*	*cecilia*	2012-05-28	Tunisia	36,651	10,161
RVcoll12N218		Nymphalidae	*Pyronia*	*cecilia*	2012-05-28	Tunisia	36,651	10,161
RVcoll13S948		Nymphalidae	*Pyronia*	*cecilia*	2013-07-05	Tunisia	36,741	8,680
RVcoll13T381		Nymphalidae	*Pyronia*	*cecilia*	2013-07-04	Tunisia	37,164	10,110
RVcoll13T398		Nymphalidae	*Pyronia*	*cecilia*	2013-07-05	Tunisia	37,313	9,856
RVcoll11Y098		Nymphalidae	*Pyronia*	*cecilia*	2011-06-29	Sicily	37,610	13,050
RVcoll12R166		Nymphalidae	*Pyronia*	*cecilia*	2012-08-04	Sicily	37,840	13,420
RVcoll12R168		Nymphalidae	*Pyronia*	*cecilia*	2012-08-04	Sicily	37,840	13,420
RVcoll11J686	-	Nymphalidae	*Pyronia*	*cecilia*	2011-07-21	Sicily	37,840	13,990
RVcoll12R038		Nymphalidae	*Pyronia*	*cecilia*	2012-08-02	Sicily	37,980	14,870
RVcoll11H711		Nymphalidae	*Pyronia*	*cecilia*	2011-06-12	Levanzo	38,000	12,330
RVcoll11H712		Nymphalidae	*Pyronia*	*cecilia*	2011-06-12	Levanzo	38,000	12,330
RVcoll11H713		Nymphalidae	*Pyronia*	*cecilia*	2011-06-12	Levanzo	38,000	12,330
RVcoll11H714		Nymphalidae	*Pyronia*	*cecilia*	2011-06-12	Levanzo	38,000	12,330
RVcoll11H717		Nymphalidae	*Pyronia*	*cecilia*	2011-06-12	Levanzo	38,000	12,330
RVcoll11H710		Nymphalidae	*Pyronia*	*cecilia*	2011-06-12	Levanzo	38,010	12,330
RVcoll11H607		Nymphalidae	*Pyronia*	*cecilia*	2011-06-08	Sicily	38,139	13,123
RVcoll12Q989		Nymphalidae	*Pyronia*	*cecilia*	2012-08-01	Sicily	38,160	15,470
RVcoll12Q991	-	Nymphalidae	*Pyronia*	*cecilia*	2012-08-01	Sicily	38,160	15,470
RVcoll12Q910		Nymphalidae	*Pyronia*	*cecilia*	2012-07-31	Italy	38,217	15,883
RVcoll12Q911		Nymphalidae	*Pyronia*	*cecilia*	2012-07-31	Italy	38,217	15,883
RVcoll11H605		Nymphalidae	*Pyronia*	*cecilia*	2011-06-08	Sicily	38,226	13,222
RVcoll14A655		Nymphalidae	*Pyronia*	*cecilia*	2013-08-19	Italy	38,389	16,194
RVcoll11H814		Nymphalidae	*Pyronia*	*cecilia*	2011-06-14	Vulcano	38,390	14,970
RVcoll11H817		Nymphalidae	*Pyronia*	*cecilia*	2011-06-14	Vulcano	38,390	14,970
RVcoll11H818		Nymphalidae	*Pyronia*	*cecilia*	2011-06-14	Vulcano	38,390	14,970
RVcollLD1340		Nymphalidae	*Pyronia*	*cecilia*	2009-06-30	Lipari	38,482	14,931
RVcollLD1435		Nymphalidae	*Pyronia*	*cecilia*	2009-06-30	Lipari	38,482	14,931
RVcoll10C681	-	Nymphalidae	*Pyronia*	*cecilia*	2010-06-30	Lipari	38,482	14,931
RVcoll11H889		Nymphalidae	*Pyronia*	*cecilia*	2011-06-15	Salina	38,560	14,840
RVcoll11H890		Nymphalidae	*Pyronia*	*cecilia*	2011-06-15	Salina	38,560	14,840
RVcoll11H893		Nymphalidae	*Pyronia*	*cecilia*	2011-06-15	Salina	38,560	14,840
LEPSS00237		Nymphalidae	*Pyronia*	*cecilia*	2015-06-28	Italy	39,247	16,220
RVcoll14A700		Nymphalidae	*Pyronia*	*cecilia*	2013-08-20	Italy	39,483	16,801
RVcoll14A701		Nymphalidae	*Pyronia*	*cecilia*	2013-08-20	Italy	39,483	16,801
RVcoll14A702		Nymphalidae	*Pyronia*	*cecilia*	2013-08-20	Italy	39,483	16,801
RVcoll15A810		Nymphalidae	*Pyronia*	*cecilia*	2015-06-04	Italy	39,814	16,309
RVcoll14L035		Nymphalidae	*Vanessa*	*atalanta*	2009-06-01	Algeria	35,280	1,070
RVcoll12N594	-	Nymphalidae	*Vanessa*	*atalanta*	2007-06-27	Algeria	35,673	3,928
RVcoll11E103		Nymphalidae	*Vanessa*	*atalanta*	2011-05-19	Maltese	35,859	14,400
RVcoll11E104		Nymphalidae	*Vanessa*	*atalanta*	2011-05-19	Maltese	35,859	14,400
RVcoll11E109		Nymphalidae	*Vanessa*	*atalanta*	2011-05-20	Maltese	36,042	14,274
RVcoll12N285		Nymphalidae	*Vanessa*	*atalanta*	2012-05-28	Tunisia	36,116	9,656
RVcoll12N243		Nymphalidae	*Vanessa*	*atalanta*	2012-05-28	Tunisia	36,294	9,811
RVcoll13T444		Nymphalidae	*Vanessa*	*atalanta*	2013-07-07	Tunisia	36,726	8,705
RVcoll11D128		Nymphalidae	*Vanessa*	*atalanta*	2011-04-08	Pantelleria	36,782	12,000
RVcoll11D135		Nymphalidae	*Vanessa*	*atalanta*	2011-04-09	Pantelleria	36,782	12,000
RVcoll11D136		Nymphalidae	*Vanessa*	*atalanta*	2011-04-09	Pantelleria	36,782	12,000
RVcoll11D114	-	Nymphalidae	*Vanessa*	*atalanta*	2011-04-08	Pantelleria	36,785	11,981
RVcoll12R094		Nymphalidae	*Vanessa*	*atalanta*	2012-08-03	Sicily	37,880	14,010
RVcoll11D184		Nymphalidae	*Vanessa*	*atalanta*	2011-04-10	Marettimo	37,961	12,067
RVcoll11D185		Nymphalidae	*Vanessa*	*atalanta*	2011-04-10	Marettimo	37,961	12,067

RVcoll11D189		Nymphalidae	*Vanessa*	*atalanta*	2011-04-10	Marettimo	37,961	12,067
RVcoll12Q913		Nymphalidae	*Vanessa*	*atalanta*	2012-07-31	Italy	38,100	15,833
RVcoll12Q596		Nymphalidae	*Vanessa*	*atalanta*	2012-08-01	Sicily	38,180	15,480
RVcoll12Q900		Nymphalidae	*Vanessa*	*atalanta*	2012-07-31	Italy	38,217	15,883
RVcoll11D433		Nymphalidae	*Vanessa*	*atalanta*	2011-04-14	Sicily	38,238	15,528
RVcoll14N646		Nymphalidae	*Vanessa*	*atalanta*	2014-09-30	Vulcano	38,380	14,970
RVcoll14N674		Nymphalidae	*Vanessa*	*atalanta*	2014-10-01	Salina	38,570	14,830
RVcoll11D512		Nymphalidae	*Vanessa*	*atalanta*	2011-04-18	Italy	38,674	15,926
RVcoll14A719		Nymphalidae	*Vanessa*	*atalanta*	2013-08-21	Italy	39,217	16,140
LEPSS00108		Nymphalidae	*Vanessa*	*atalanta*	2014-10-26	Italy	39,237	16,297
RVcoll15A824		Nymphalidae	*Vanessa*	*atalanta*	2015-06-06	Italy	39,388	16,602
RVcoll15A802		Nymphalidae	*Vanessa*	*atalanta*	2015-06-04	Italy	39,908	16,145
RVcoll12Q788		Nymphalidae	*Vanessa*	*atalanta*	2012-07-30	Italy	39,930	16,170
RVcoll14L049		Nymphalidae	*Vanessa*	*cardui*	2009-05-13	Algeria	35,120	0,830
RVcoll11J924		Nymphalidae	*Vanessa*	*cardui*	2011-11-25	Algeria	35,220	4,190
RVcoll11J932		Nymphalidae	*Vanessa*	*cardui*	2011-12-02	Algeria	35,220	4,190
RVcoll14L162	-	Nymphalidae	*Vanessa*	*cardui*	-	Algeria	35,860	4,750
RVcoll14D500		Nymphalidae	*Vanessa*	*cardui*	-	Maltese	35,927	14,442
RVcoll14D501		Nymphalidae	*Vanessa*	*cardui*	-	Maltese	35,927	14,442
RVcoll10C596		Nymphalidae	*Vanessa*	*cardui*	2010-06-17	Maltese	35,927	14,442
RVcoll10C597		Nymphalidae	*Vanessa*	*cardui*	2010-06-17	Maltese	35,927	14,442
RVcoll12N619		Nymphalidae	*Vanessa*	*cardui*	2012-05-12	Algeria	36,257	5,245
RVcoll12N267		Nymphalidae	*Vanessa*	*cardui*	2012-05-28	Tunisia	36,294	9,811
RVcoll12N599		Nymphalidae	*Vanessa*	*cardui*	2012-05-10	Algeria	36,592	4,611
RVcoll12N493		Nymphalidae	*Vanessa*	*cardui*	2012-06-01	Tunisia	36,731	8,710
RVcoll12N504		Nymphalidae	*Vanessa*	*cardui*	2012-06-01	Tunisia	36,764	8,666
RVcoll11D127		Nymphalidae	*Vanessa*	*cardui*	2011-04-08	Pantelleria	36,782	12,000
RVcoll13T620		Nymphalidae	*Vanessa*	*cardui*	2013-07-10	Tunisia	36,837	10,584
RVcoll11D209		Nymphalidae	*Vanessa*	*cardui*	2011-04-11	Sicily	37,838	13,429
RVcoll12R130		Nymphalidae	*Vanessa*	*cardui*	2012-08-03	Sicily	37,840	14,060
RVcoll11D182		Nymphalidae	*Vanessa*	*cardui*	2011-04-10	Marettimo	37,961	12,067
RVcoll11D183		Nymphalidae	*Vanessa*	*cardui*	2011-04-10	Marettimo	37,961	12,067
RVcoll12M567		Nymphalidae	*Vanessa*	*cardui*	2012-05-06	Sicily	37,993	14,869
RVcoll11H533		Nymphalidae	*Vanessa*	*cardui*	2011-06-08	Sicily	38,093	13,260
RVcoll11I117		Nymphalidae	*Vanessa*	*cardui*	2011-06-19	Italy	38,221	16,183
RVcoll14N645		Nymphalidae	*Vanessa*	*cardui*	2014-09-30	Vulcano	38,380	14,970
RVcoll14A662		Nymphalidae	*Vanessa*	*cardui*	2013-08-19	Italy	38,389	16,194
RVcoll14N671		Nymphalidae	*Vanessa*	*cardui*	2014-10-01	Salina	38,570	14,830
RVcoll10C684		Nymphalidae	*Vanessa*	*cardui*	2012-07-01	Stromboli	38,803	15,228
RVcoll14A717		Nymphalidae	*Vanessa*	*cardui*	2013-08-21	Italy	39,217	16,140
LEPSS00106		Nymphalidae	*Vanessa*	*cardui*	2014-05-23	Italy	39,315	16,528
LEPSS00105		Nymphalidae	*Vanessa*	*cardui*	2014-05-20	Italy	39,443	16,604
RVcoll15A806		Nymphalidae	*Vanessa*	*cardui*	2015-06-04	Italy	39,814	16,309
RVcoll11I192		Nymphalidae	*Vanessa*	*cardui*	2011-06-20	Italy	39,930	16,150

Supplementary Results

Supplementary Table S3 Results for the Generalized Linear Models, using contemporary variables. Abbreviations are: Est = estimated parameter; SE = Standard Error; t = t value; P = P value; lmg = percentage of explained variance attributed by hierarchical partition of variance; MT = mean annual temperature; AP =annual precipitation; IA = island area; EL = maximum elevation of island; IS =, island isolation from the nearest source; SR = butterfly richness of the nearest source.

	**Contemporary factors**				
	Est.	S.E.	t	P	lmg%
MT					
AP					
IA	0.110	0.037	2.952	0.006	17.0
EL	0.086	0.056	1.525	0.139	7.4
IS	-0.191	0.036	-5.298	<0.001	26.1
SR	0.562	0.136	4.138	<0.001	18.3
					**67.8%**

Supplementary Table S4 Results for the Generalized Linear Models, using contemporary variables and the Pleistocene connection as a factor variable. Abbreviations Abbreviations as for Table S2 except, PC = occurrence of Pleistocene connection.

	**Contemporary factors + Pleistocene connection**				
	Est.	S.E.	t	P	lmg%
MT					
AP	0.0003	0.0002	1.665	0.108	12.7
IA	0.074	0.038	1.968	0.060	14.6
EL	0.181	0.067	2.690	0.013	11.8
IS	-0.074	0.052	-1.423	0.167	11.6
SR	0.439	0.151	2.900	0.008	13.3
PC	0.181	0.069	2.607	0.015	11.7
					**75.8%**

Supplementary Table S5 Spearman rho correlation and associated P value for the relationship island occurrence and IDV for the butterfly assemblage of each island. The Q1- Q4 columns indicate the number of species occurring in each of the four sectors of the occurrence-IDV scatterplot for each island and the associated P value for sectors (in parentheses). Q1 represents the top-right sector (widespread-diversified), Q2 top-left (uncommon-diversified), Q3 bottom-left (uncommon-not diversified), Q4 bottom-right (widespread-not diversified).

Island	Spearman rho	P	Q1	Q2	Q3	Q4
All	0.051	0.791	**4 (0.048)**	7 (0.382)	8 (0.560)	10 (0.842)
Lampedusa	-0.054	0.855	4 (0.522)	**0 (0.000)**	3 (0.275)	**7 (0.966)**
Levanzo	-0.451	0.069	3 (0.170)	3 (0.162)	**2 (0.048)**	**9 (0.984)**
Linosa	-0.798	**0.032**	2 (0.444)	**0 (0.000)**	**0 (0.000)**	**5 (0.989)**
Lipari	-0.656	**<0.001**	4 (0.139)	7 (0.646)	**2 (0.018)**	**10(0.958)**
Maltese is.	-0.427	0.068	4 (0.275)	4 (0.277)	**1 (0.003)**	**10 (0.991)**
Marettimo	-0.850	**0.007**	1 (0.103)	**0 (0.000)**	**0 (0.000)**	**7 (1.000)**
Pantelleria	-0.326	0.255	4 (0.511)	**0 (0.000)**	**1 (0.021)**	**9 (0.997)**
Salina	-0.650	**0.002**	4 (0.211)	5 (0.413)	**2 (0.029)**	**9 (0.951)**
Stromboli	-0.524	**0.045**	2 (0.074)	2 (0.080)	**1 (0.020)**	**10 (1.000)**
Ustica	-0.103	0.705	4 (0.421)	**1 (0.014)**	2 (0.066)	**9 (0.994)**
Vulcano	-0.256	0.250	4 (0.144)	5 (0.319)	3 (0.056)	**10 (0.977)**

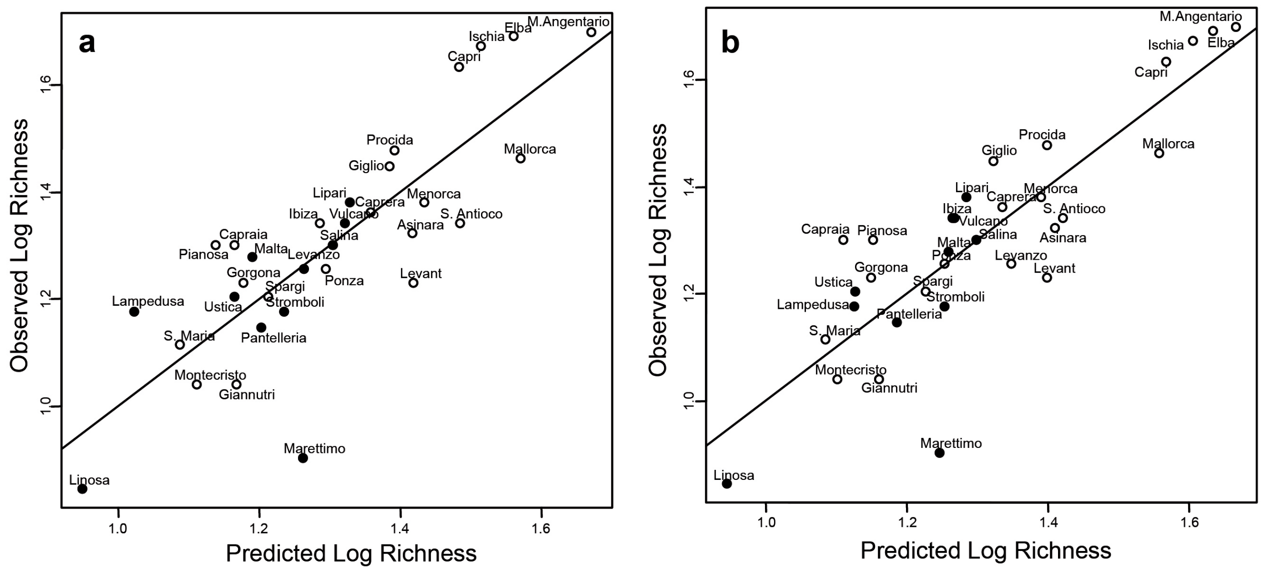

Supplementary Figure S1 Predicted and observed species richness (log transformed) obtained with Generalized Linear Models. Black dots represent the islands analysed in this study, while the white dots indicate the rest of the western Mediterranean islands. Using only contemporary variables(above). The Maltese islands, Lampedusa, Lipari and Vulcano were richer in species than expected, with rounded residuals of +4, +4, +3 and +1 species respectively, while Pantelleria, Linosa, Stromboli and Marettimo had less species than expected, with residuals of -2 for the first three islands and -10 for the last. Levanzo, Salina and Ustica had the same observed and predicted richness. Adding the Pleistocene connection as a variable (below) the species richness on Salina was almost perfectly predicted. The Maltese islands, Lampedusa, Ustica, Vulcano and Lipari were richer than expected and Pantelleria, Levanzo, Stromboli, Marettimo and Linosa had negative residuals.

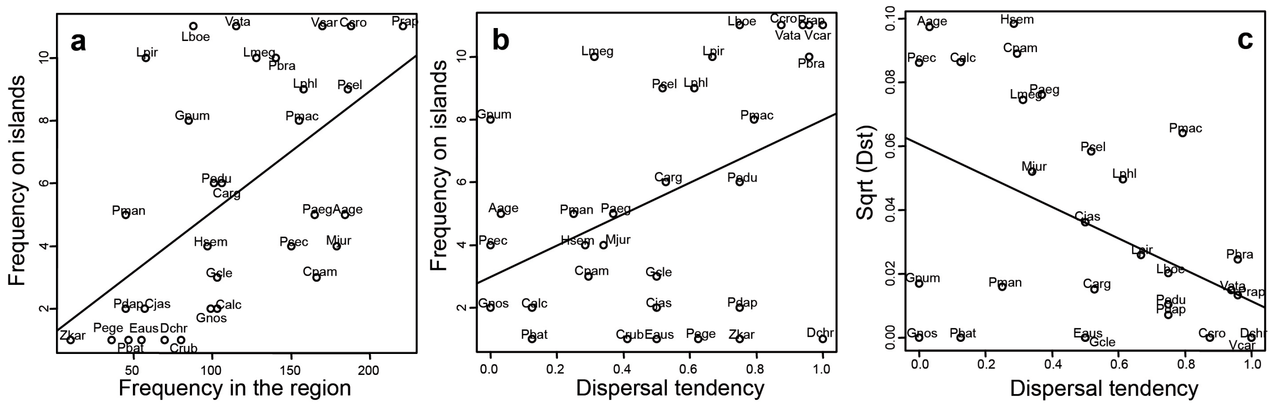

Supplementary Figure S2 (a) The correlations between species occurrence on islands and their frequency at source in cells of 0.1×0.1 latitude-longitude degrees and (b) the correlations between species occurrence on islands and their dispersal tendency; the size of the circles is proportional with the number of species having identical values. (c) The correlation between IDV and dispersal tendency is non-significant. Species names were abbreviated as follows: *Aricia agestis/cramera* (Aage), *Callophrys rubi* (Crub), *Carcharodus alceae* (Calc), *Celastrina argiolus* (Carg), *Charaxes jasius* (Cjas), *Coenonympha pamphilus* (Cpam), *Colias croceus* (Ccro), *Danaus chrysippus* (Dchr), *Euchloe ausionia* (Eaus), *Hipparchia semele/algirica/blachieri/leighebi* (Hlei), *Gegenes nostrodamus* (Gnos), *Gegenes pumilio* (Gpum), *Gonepteryx cleopatra* (Gcle), *Lampides boeticus* (Lboe), *Lasiommata megera* (Lmeg), *Leptotes pirithous* (Lpir), *Lycaena phlaeas* (Lphl), *Maniola jurtina* (Mjur), *Papilio machaon* (Pmac), *Pararge aegeria* (Paeg), *Pieris brassicae* (Pbra), *Pieris mannii* (Pman), *Pieris rapae* (Prap), *Polygonia egea* (Pege), *Polyommatus celina* (Pcel), *Pontia daplidice* (Pdap), *Pontia edusa* (Pedu), *Pseudophilotes baton* (Pbat), *Pyronia cecilia* (Pcec), *Vanessa atalanta* (Vata), *Vanessa cardui* (Vcar) and *Zizeeria karsandra* (Zkar).
