# Supplementary material for: Historical and contemporary factors generate unique butterfly communities on islands
Source: Sci Rep. 2016 Jun 29;6:28828. doi: 10.1038/srep28828 (PMC4926222; doi:10.1038/srep28828)
Supplement: Supplementary Note [file srep28828-s2.pdf]

Supplementary Note

Historical and contemporary factors generate unique butterfly communities on islands

Raluca Vodă, Leonardo Dapporto, Vlad Dincă, Tim G. Shreeve, Mourad Khaldi, Ghania Barech, Khellaf Rebbas, Paul Sammut, Stefano Scalercio, Paul D.N. Hebert, Roger Vila

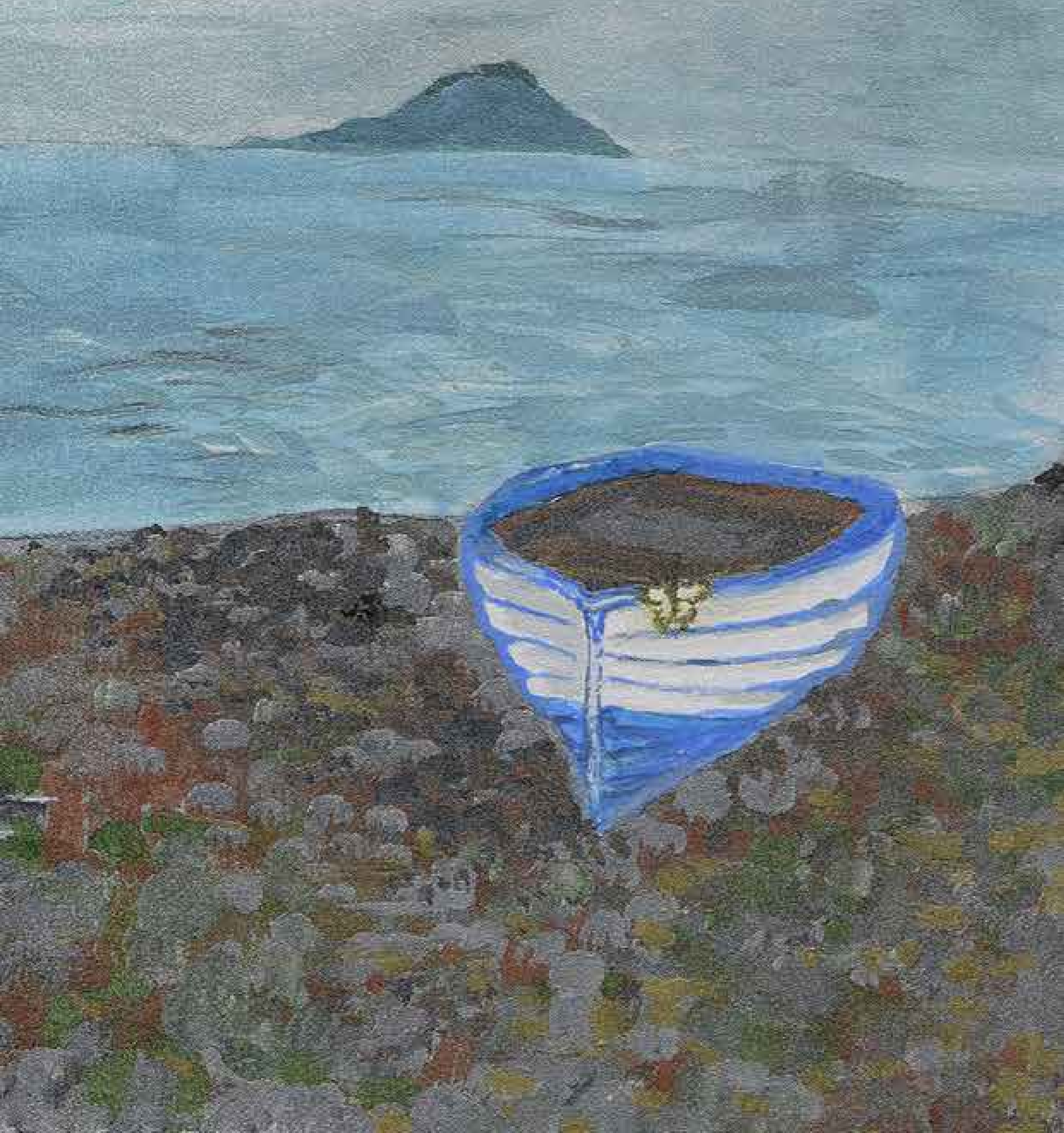

In this Appendix we provide haplotype networks (produced with the TCS Network method in PopART, http://popart.otago.ac.nz, Clement et al. 2002) for all the 29 investigated species (see the methods in the main paper). In addition to the haplotype networks, we represented the genetic variation by projecting the genetic distances among the examined specimens in two-dimensional graphs (circles) by employing the Principal Coordinates Analysis (PCoA). We overlapped the PCoA diagram with a RGB color space, therefore the more similar two haplotypes, the more similar their colors in this graph. To visualize the similarity patterns of the populations in the study area we plotted the color obtained for each sequenced specimen from each area by using pie charts.

Analyses of single species

Papilionidae

Papilio machaon Linnaeus, 1758

This species is commonly occurring throughout the western Mediterranean, but usually on small islands only a reduced number of specimens can be found. In the study area it is reported from several islands: Lampedusa, Pantelleria, the Maltese islands, Ustica, Vulcano, Lipari, Salina and Stromboli (Sammut, 1984; Aistleitner & Aistleitner, 2001; Biermann, 2005; Balletto et al., 2005, Seizmair, 2014). We confirm its presence on all of the islands except for Stromboli and Pantelleria. It has never been seen on Levanzo and Marettimo, but on Aegadian Islands it has been reported from Favignana (Aistleitner & Aistleitner, 2001). There are several different haplotypes across the studied region and, although the genetic differences among the specimens are small, there is a constant and clear separation of five mutations (0.8%) between the populations from Maghreb (including Lampedusa) and Europe (Fig. 1).

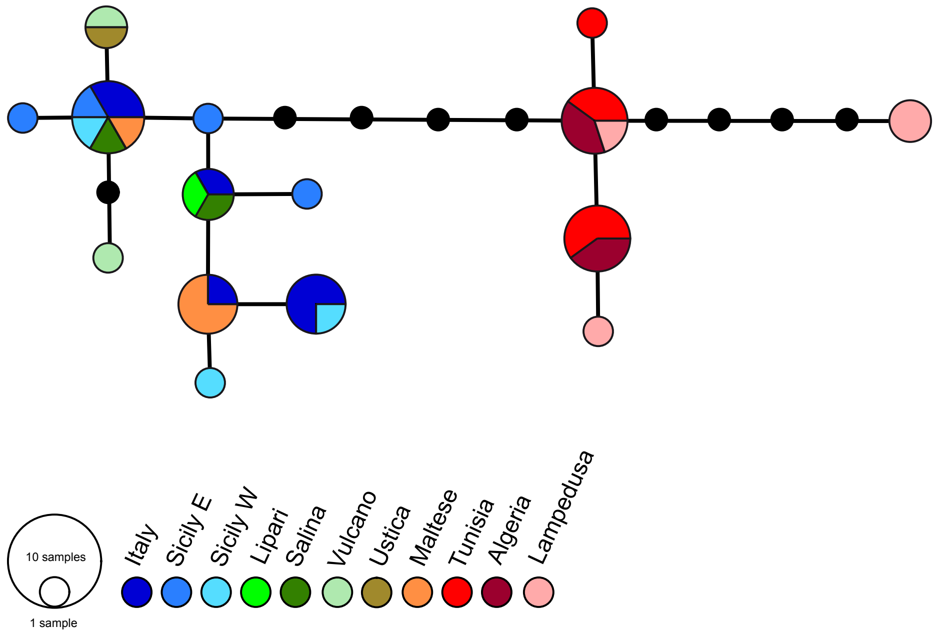

Figure 1 COI haplotype network for 38 specimens of *P. machaon* from the study area.

Many subspecies and local forms of this species have been described, including the ssp. *melitensis* Eller, 1936, considered as endemic for the Maltese islands. However, according to the genetic results, this population has the same haplotype as specimens occurring in Calabria, suggesting that the subspecies described for the Maltese islands is a local morph. This species has good dispersal capabilities and it is highly probable that the populations from the Aeolian and Aegadian Islands form a metapopulation together with the ones occurring in Sicily and Calabria. However, the existence of

two clades (European and north African) and of a different morphological race on the Maltese islands suggests that dispersal over longer distances is unlikely. It has to be noted that among the three haplotypes found in Lampedusa, two were not found in the Maghreb. Two specimens showed a COI sequence having five mutations (0.8%) with respect to the closest haplotype found in north Africa. It is possible that a wider survey of Maghrebian populations of *P. machaon* will allow the detection of these haplotypes, but for the moment most specimens from Lampedusa show a highly diverging genetic fingerprint.
By projecting the PCoA results on the geographic map it is possible to directly evaluate this pattern, with the Maghreb forming a different group from Sicily-Calabria and all circum-Sicilian islands (except for Lampedusa) showing a very similar genetic composition to Sicily (Fig. 2).

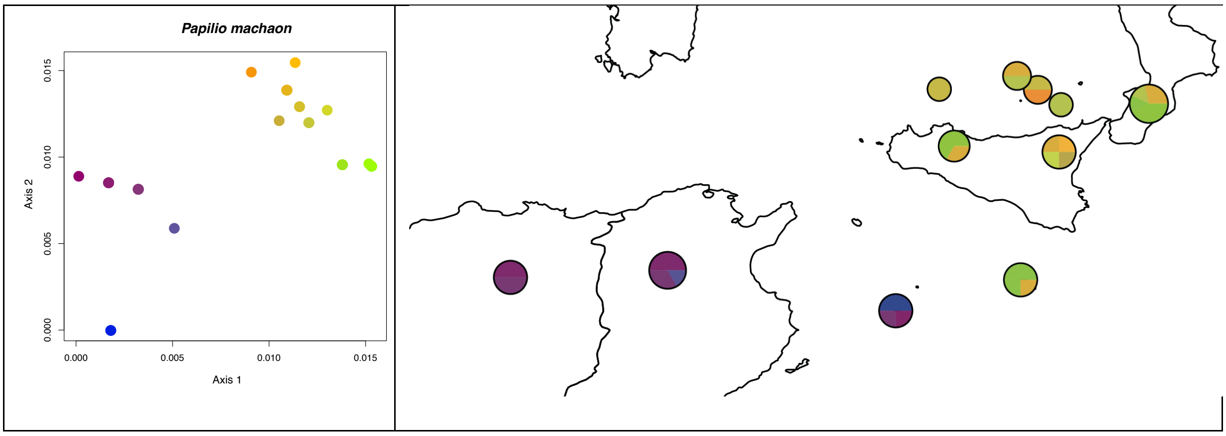

Figure 2 PCoA projection (left) of genetic distances among specimens (dots) in the bidimensional RGB color space. Individual colors have been subsequently plotted on the map (right). The map used is freely available in the R package 'rworldxtra' at CRAN (https://cran.r-project.org/web/packages/rworldxtra/index.html).

Pieridae
Pieris rapae (Linnaeus, 1758)

This species probably represents the most widespread and common butterfly in the Mediterranean region, including the islands (Balletto et al., 2005). Over the study area it has been reported for all the islands (Sammut, 1984; Aistleitner & Aistleitner, 2001; Balletto et al., 2005; Seizmair, 2014) and we confirmed its presence in recent years. Interestingly, we encountered *P. rapae* on the Aeolian Islands during all our field trips but Kudrna and Leigheb (1988) did not report this species for any of these islands, even though they conducted several days of fieldwork. *Pieris rapae* was strangely not reported from several Tyrrhenian islands either, with the exception of Ischia. However, Kudrna and Leigheb recorded *P. napi* for all the Aeolian and Tyrrhenian islands they investigated, and in some cases (Ponza and all the Aeolian islands) these were the first records. The *P. napi* specimens collected by Kudrna and Leigheb from these islands are deposited in the Museo di Scienze Naturali dell'Alto Adige. The museum confirmed that the only *P. napi* specimens present in the collection are from Ischia. Therefore, we suspect that Kudrna and Leigheb (1988) switched the records between *P. napi* and *P. rapae* and that the only reliable record for *P. napi* in their study is for Ischia.
Pieris rapae is reported to perform directional migrations in Europe and in the Mediterranean (Stefanescu et al., 2003; John et al., 2008). The haplotype network indicates the existence of differentiated haplotypes but, as expected for a highly dispersive species, their distribution shows no geographic pattern (Fig 3 and 4).

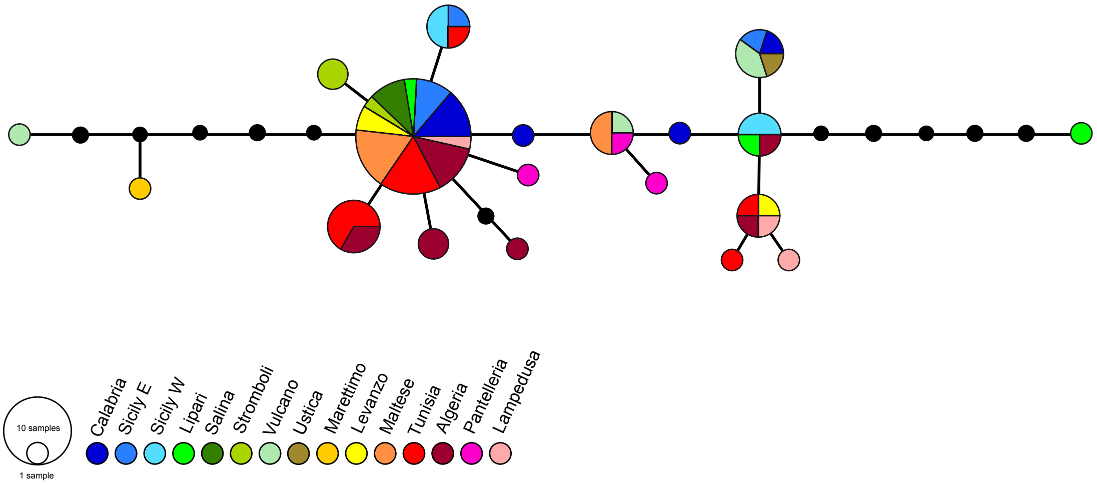

Figure 3 COI haplotype network for 70 specimens of *P. rapae* from the study area.

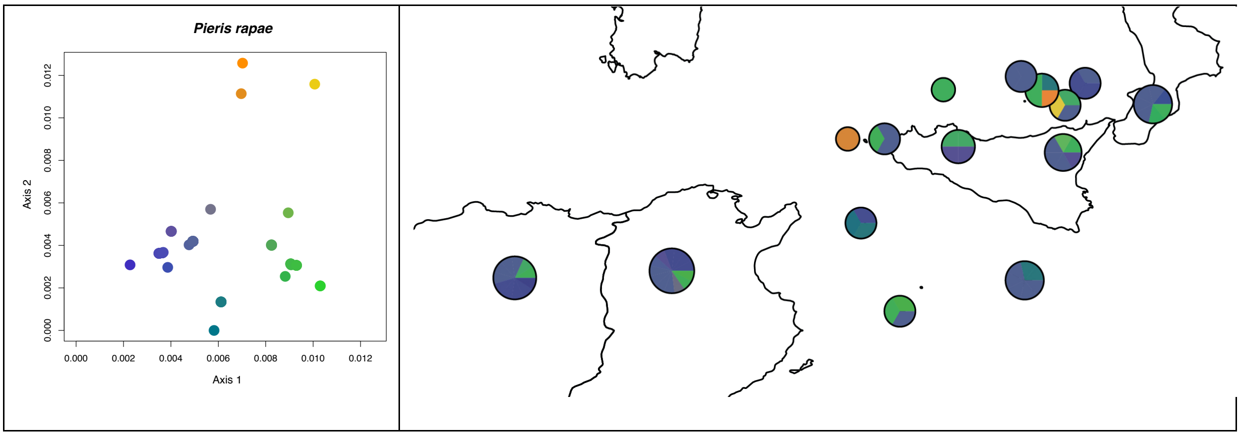

Figure 4 The PCoA representation in the RGB color space and distribution map show very little diversification among the populations of *P. rapae*. The map used is freely available in the R package 'rworldxtra' at CRAN (https://cran.r-project.org/web/packages/rworldxtra/index.html).

Pieris brassicae (Linnaeus, 1758)

This species shows a pattern of distribution very similar to *P. rapae*. However, reports for Linosa are missing so far (Sammut, 1984; Aistleitner & Aistleitner, 2001; Biermann, 2005; Balletto et al., 2005; Seizmair, 2014) and we did not observe it during our visits to the island either. This species too is reported to migrate over Europe and the Mediterranean (Stefanescu et al., 2003) and, accordingly, the observed generally low genetic variation (Fig. 5) showed no marked spatial pattern (Fig. 6). The relatively diverged haplotype from Algeria, detected in a single specimen (Fig. 5), is likely to be more widespread and would probably be found more frequently with increased sampling and geographical area.

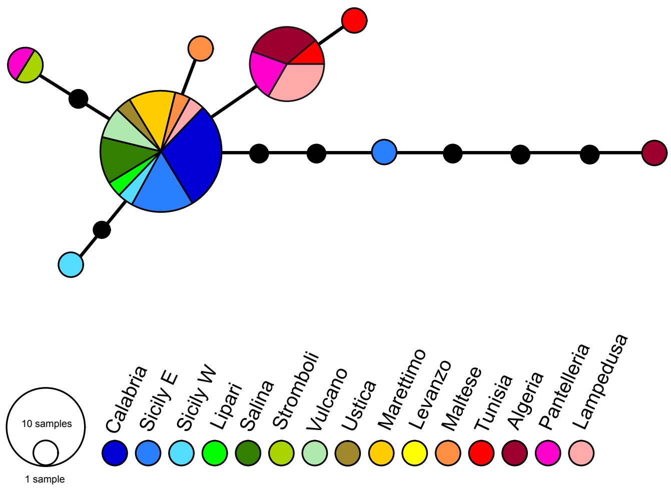

Figure 5 COI haplotype network for 42 specimens of *P. brassicae* from the study area.

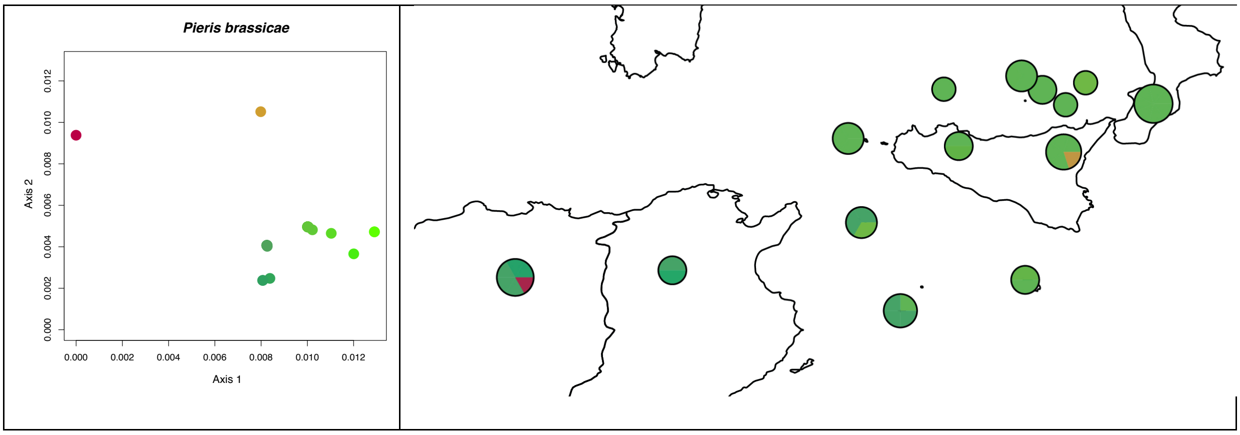

Figure 6 PCoA representation in the RGB color space and its projection on the map show extremely weak diversification among the populations of *P. brassicae.* The map used is freely available in the R package 'rworldxtra' at CRAN (https://cran.r-project.org/web/packages/rworldxtra/index.html).

Pieris mannii (Mayer, 1851)

This species is not reported from Tunisia and Algeria (Tennent, 1996) and most reports for Sicily are from the northeastern part of the island (Balletto et al., 2005). For the circum-Sicilian islands it is reported only from Lipari, Vulcano, Salina and Stromboli (Kudrna & Leigheib, 1988). We also collected a specimen in Levanzo. Like the other congeneric species, *P. mannii* showed a simple genetic structure (Fig. 7) lacking any marked spatial genetic pattern (Fig. 8).

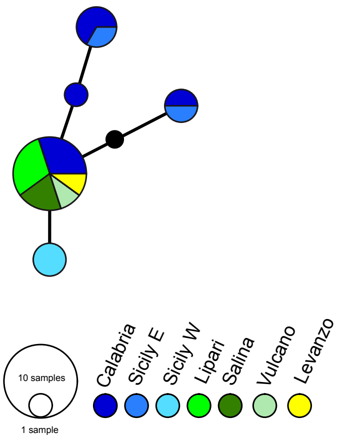

Figure 7 COI haplotype network for 18 specimens of *P. mannii* from the study area.

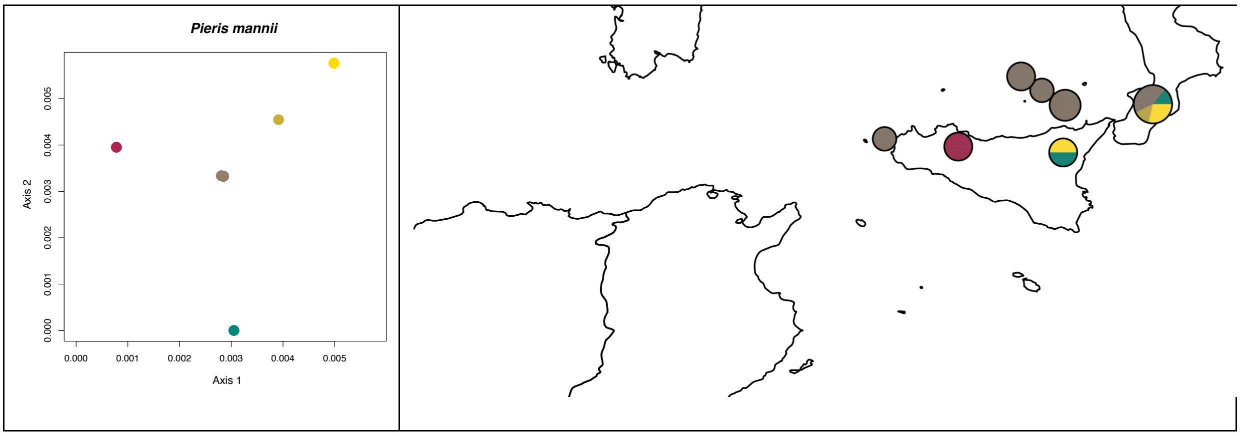

Figure 8 PCoA representation in the RGB color space and its projection on the map showing unclear patterns of differentiation in the slightly diversified specimens of *P. mannii*. The map used is freely available in the R package 'rworldxtra' at CRAN (https://cran.r-project.org/web/packages/rworldxtra/index.html).

Pontia edusa (Fabricius, 1777)

Pontia edusa has been separated from the sibling species *Pontia daplidice* on the basis of several allozyme markers (Geiger et al., 1988; Wegener, 1988) and mtDNA (John et al., 2013). *Pontia edusa* occurs in the Italian Peninsula, Sicily and eastern Europe, while *P. daplidice* flies in north Africa, Cyprus, and western Europe (Balletto et al., 2005; Tolman & Lewington, 2008; John et al., 2013). In the study area, *P. edusa* was reported for the Aeolian Islands (but not from Salina), Levanzo, Ustica and the Maltese Islands (Balletto et al., 2005; Seizmair, 2014). However, these reports were not based on allozyme or DNA analyses. Our DNA data confirmed its presence on Lipari, Vulcano, Stromboli, Ustica and the Maltese Islands, where two haplotypes have been found (Fig. 9), which did not display any notable spatial genetic structure (Fig. 10).

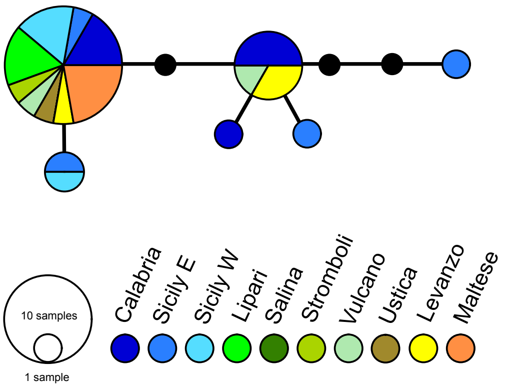

Figure 9 COI haplotype network for 29 specimens of *P. edusa* from the study area.

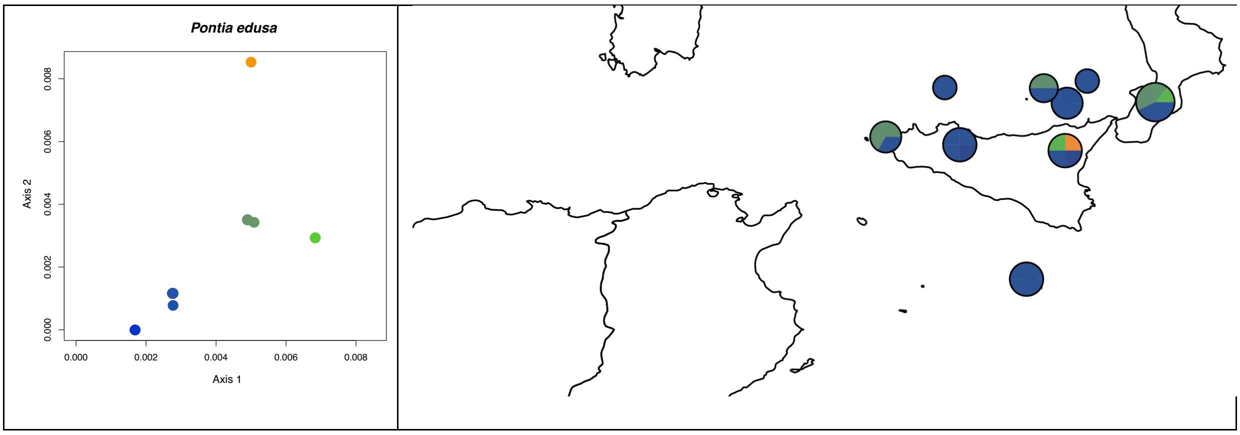

Figure 10 PCoA representation in the RGB color space and its projection on the map showing unclear patterns of differentiation in the slightly diversified specimens of *P. edusa*. The map used is freely available in the R package 'rworldxtra' at CRAN (https://cran.r-project.org/web/packages/rworldxtra/index.html).

Pontia daplidice (Linnaeus, 1758)

We recorded this species only from the Maghreb and Lampedusa, which is located rather close to its faunistic source - Tunisia. It was also reported from Pantelleria in 1995 (Romano & Romano, 1995), but we did not find it during our three visits to the island. Three of the eight haplotypes detected in our study area are present on Lampedusa (Fig. 11), but no overall spatial genetic structure has been observed (Fig. 12). This confirms that *P. daplidice* and *P. edusa* have high dispersal capabilities (Bolotov et al., 2013), suggesting that the pattern of mutual exclusion between north Africa and Sicily-Italian peninsula is likely maintained by mechanisms other than limited dispersal (Vodă et al., 2015).

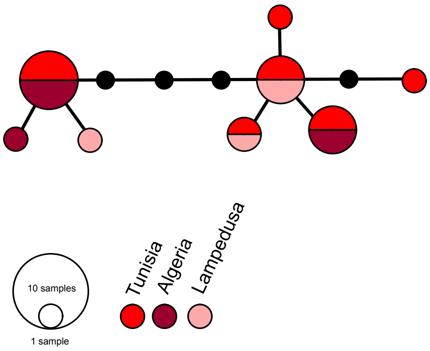

Figure 11 COI haplotype network for 20 specimens of *P. daplidice* from the study area.

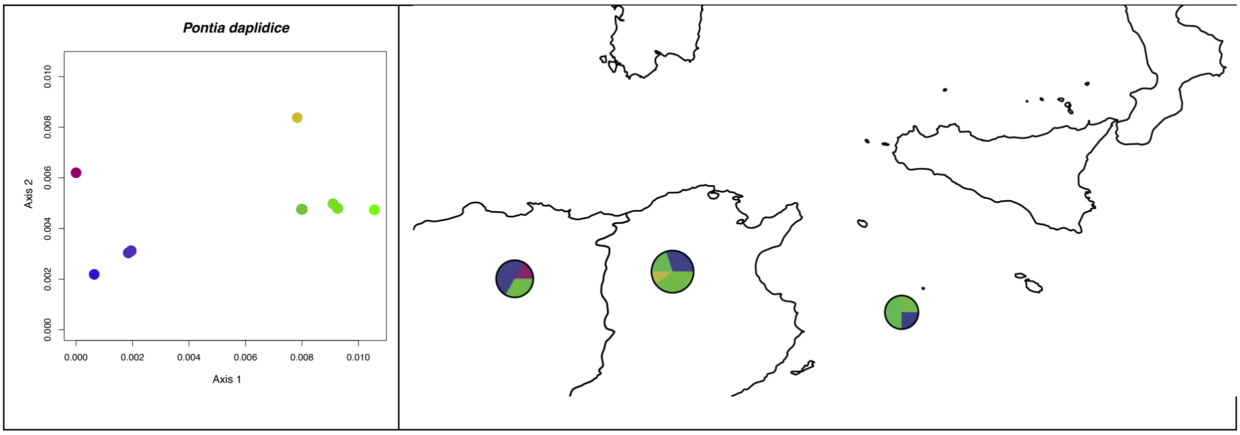

Figure 12 PCoA representation in the RGB color space and its projection on the map showing no pattern of differentiation in *P. daplidice*. The map used is freely available in the R package 'rworldxtra' at CRAN (https://cran.r-project.org/web/packages/rworldxtra/index.html).

Euchloe ausonia (Hübner, 1805)

This species occurs in southern, central and eastern Europe and in several Mediterranean islands. In western Europe and Maghreb it is replaced by a morphologically similar species, *E. crameri*. For circum-Sicilian islands it has only been reported in Ustica (Seizmair, 2014). Only a single haplotype has been found in the study area (Fig. 13).

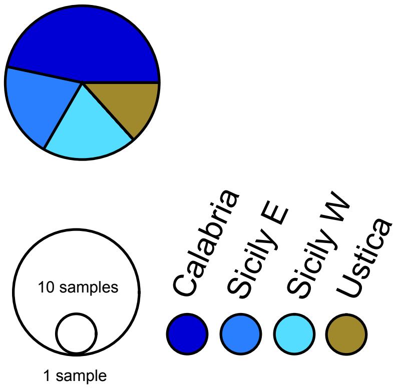

Figure 13 COI haplotype network for 15 specimens of *E. ausonia* from the study area.

Colias croceus (Geoffroy, 1785)

This species represents a constant presence in the European-Mediterranean region, including all the islands under study (Sammut, 1984; Balletto et al., 2005; Seizmair, 2014) and in recent years we confirmed its presence on most of them. This species shows directional migrations in Europe and in the Mediterranean (Stefanescu et al., 2003). Accordingly, no genetic structure has been found over the study area (Fig. 14).

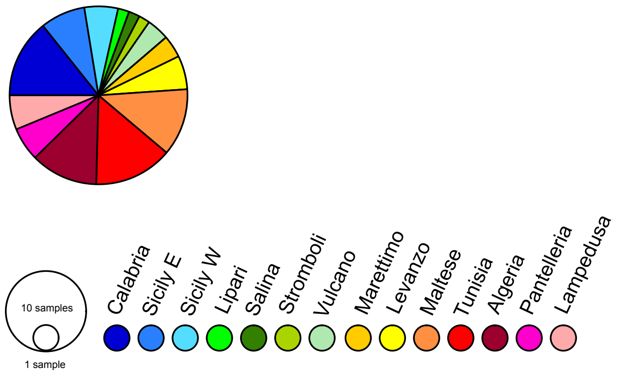

Figure 14 COI haplotype network for 50 specimens of *C. croceus* from the study area.

Gonepteryx cleopatra (Linnaeus, 1767)

This species is widespread and common in Mediterranean areas characterized by maquis. Although it represents a frequent presence on mainland and large islands, it is uncommon on small islands and over the study area it was reported only for Malta, Ustica and Levanzo (Sammut, 1984; Balletto et al., 2005; Seizmair, 2014). We confirmed its presence on Malta and Levanzo. This species showed no genetic variability since the 21 specimens analyzed shared the same haplotype from Algeria to the Italian Peninsula, and therefore we provide only the representation of the haplotype network (Fig. 15).

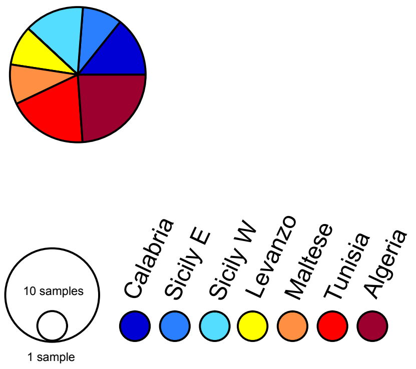

Figure 15 COI haplotype network for 21 specimens of *G. cleopatra* with a single haplotype detected in the study area.

Lycaenidae

Aricia agestis (Denis & Schiffermüller, 1775) & *Aricia cramera* (Eschscholtz, 1821)

These are taxa with very similar morphology that have often been subjected to taxonomical controversy. The taxon *cramera* has often been treated as a subspecies of *agestis* (e.g. Higgins & Riley, 1970; Tolman & Lewington, 2008; Tshikolovets, 2011), but also as a distinct species, based on crossing experiments and on differences in the morphology of the genitalia (Higgins, 1975; Balletto et al., 1981; Kudrna et al., 2011). Recent molecular data support the species status of the two taxa (Sañudo-Restrepo et al., 2013; Vodă et al., 2015), but in this study we treated them as a group since they show a minimum COI p-distance lower than 3%.

Aricia agestis and *A. cramera* are parapatric, with a contact zone in northeastern Iberian Peninsula, and they have never been found in sympatry on any Mediterranean island. Vodă et al. (2015) showed that in Sicily and the Aeolian Islands *A. agestis* has a slightly different genetic pattern with respect to Calabria, pattern that we also confirm in this study (Fig. 16 and 17). In the area we investigated, *A. cramera* occurs in the Maghreb, and *A. agestis* in Italy, Sicily, Vulcano, Lipari, Ustica and the Maltese Islands (Sammut, 1984; Balletto et al., 2005; Seizmair 2014). Recent observations suggest

that this species has dramatically declined or is even extinct in the Maltese islands (Sammut & Borg, 2008). We sampled this species in Italy, Sicily, Lipari and Vulcano.
Mitochondrial DNA shows that, while *A. cramera* is represented by a single haplotype detected in Algeria and Tunisia, the populations of *A. agestis* from islands (Sicily, Lipari and Vulcano) do not share any haplotypes with conspecifics on mainland. Moreover, Sicily hosts a relatively high genetic diversity (four different haplotypes compared to eight detected on the Italian mainland), while Lipari and Vulcano host some haplotypes that are absent from Sicily as well. This interesting pattern suggests that the island haplotypes are either relicts that have gone extinct on mainland, or represent the outcome of local evolution in isolation. In any case, they confer genetic uniqueness to the island populations. Due to the lack of recent specimens, the population from the Maltese islands has been inspected only by means of genitalia, and the males revealed a typical *Aricia agestis* morphology.

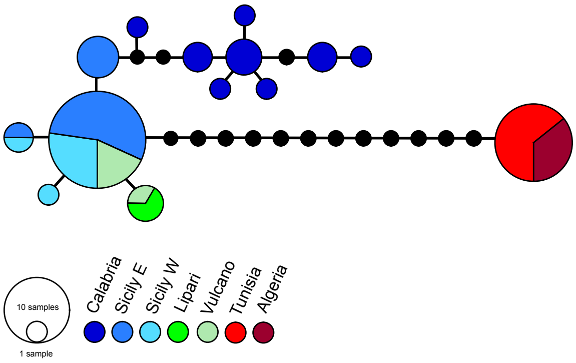

Figure 16 COI haplotype network for 58 specimens of *A. agestis* and *A. cramera* from the study area.

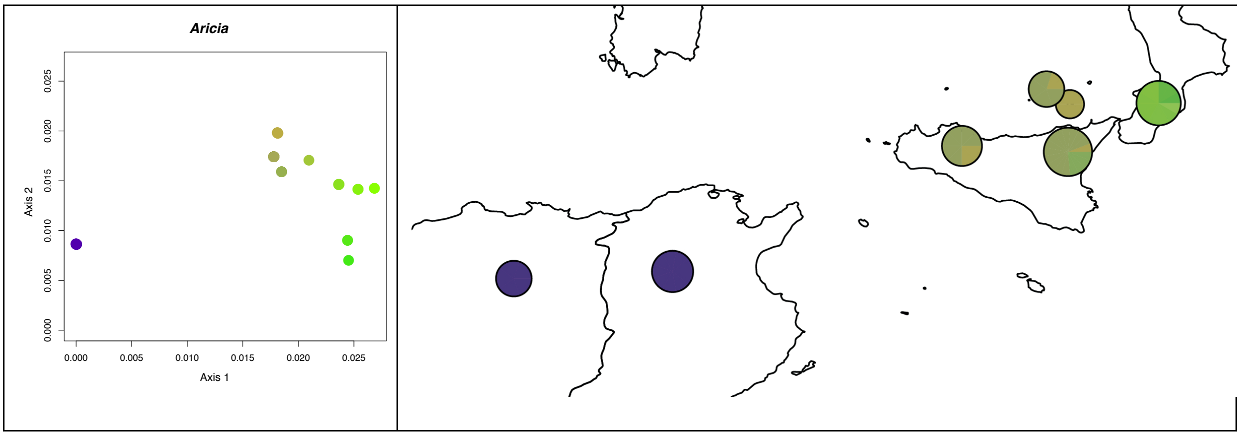

Figure 17 PCoA representation in the RGB color space and its projection on the map showing a strong differentiation between *A. agestis* (Italian Peninsula, Sicily, circum-Sicilian islands) and *A. cramera* (Tunisia and Algeria). A slight degree of differentiation is also visible between Calabria and Sicily. The map used is freely available in the R package 'rworldxtra' at CRAN (https://cran.r-project.org/web/packages/rworldxtra/index.html).

Polyommatus celina (Austaut, 1879)

Until recently, the taxon *celina* was treated as a subspecies or a form of *P. icarus* from which it was distinguished on the basis of minor morphological differences in wing pattern (Tolman & Lewington, 2008; Tarrier & Delacre, 2008). Recent molecular studies (Wiemers et al., 2010; Dincă et al., 2011) showed that *P. celina* is a distinct species, occurring throughout the Maghreb, southern and central Iberian Peninsula, and on several western Mediterranean islands, where it was never found in sympatry with *P. icarus* (Vodă et al., 2015). In the study area *P. celina* forms two main clades; one occurs in the Maghreb, Lampedusa and Pantelleria, and the other in Sicily and the rest of the circum-Sicilian islands (Fig. 18, 19). Up to now, both lineages have been found in sympatry only on the

Maltese islands, thus showing a very clear pattern of regional diversification (Fig. 18). We confirmed the presence of this species on all the islands from which it was reported.

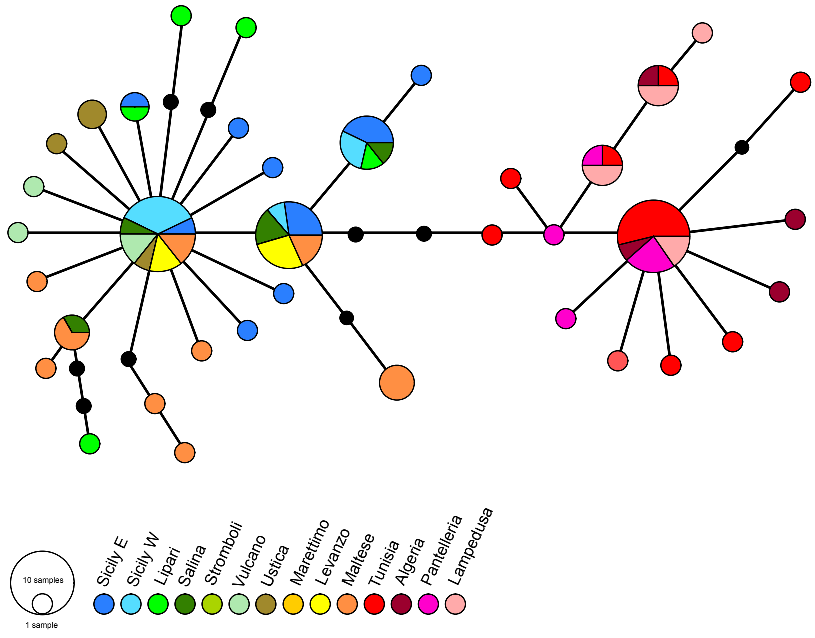

Figure 18 COI haplotype network for 90 specimens of *P. celina* from the study area.

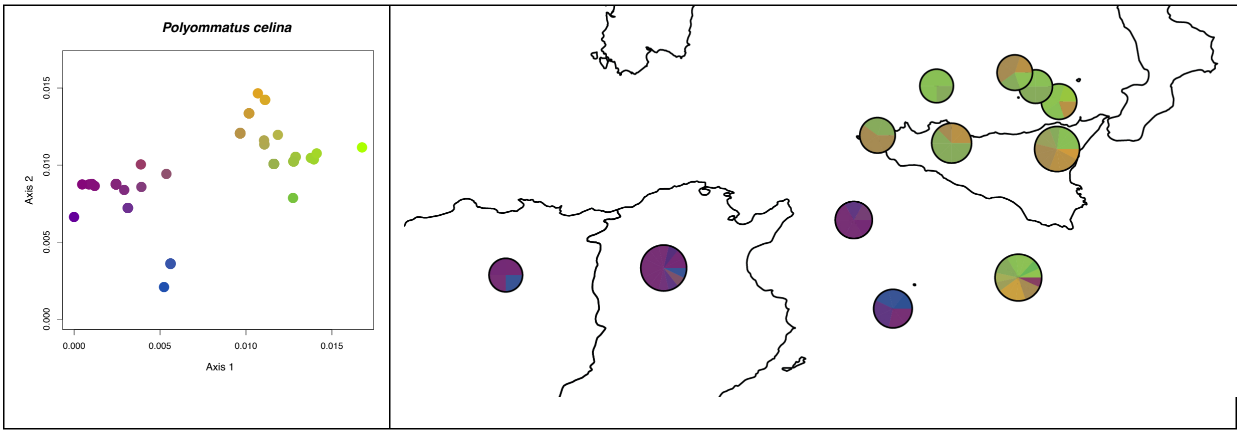

Figure 19 PCoA representation in the RGB color space and its projection on the map showing a strong differentiation between the lineages of *P. celina* occurring between Sicily and the Maghreb. Pantelleria and Lampedusa showed typical north African populations, while the remnant circum-Sicilian islands were very similar to the Sicilian populations. The map used is freely available in the R package 'rworldxtra' at CRAN (https://cran.r-project.org/web/packages/rworldxtra/index.html).

Celastrina argiolus (Linnaeus, 1758)

It is a widespread and common lycaenid, occurring in the Maghreb and most of Europe, including on the islands of the Mediterranean (Balletto et al., 2005; Tolman & Lewington, 2008). The wide distribution of this species is mainly due to its high ecologic adaptability, being often sighted in anthropic habitats, such as parks or agricultural fields. This characteristic is also reflected in its genetic signature, which has low variation across its entire range (Fig. 20, 21). However, in the study area, this species does not occur everywhere and it is present only on the Aeolian Islands, Pantelleria and the Maltese islands (Balletto et al., 2005; Sammut, 1984). We also confirmed this pattern during our investigations. It has to be noted that on Malta this species was not recorded until 1961 (Valletta,

1961). We found three mitochondrial haplotypes on the island, suggesting that this species experienced more than one colonization event, although it is not sure if its presence has only been overlooked, or if all colonizations events occurred since the ´60s.

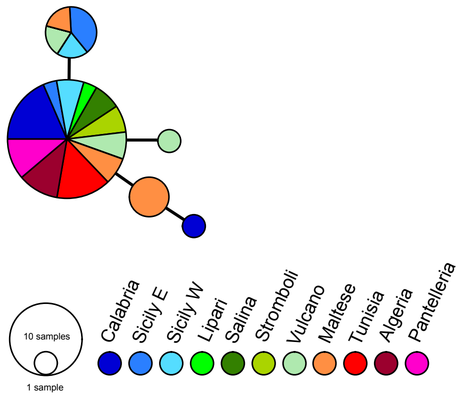

Figure 20 COI haplotype network for 37 specimens of *C. argiolus* from the study area.

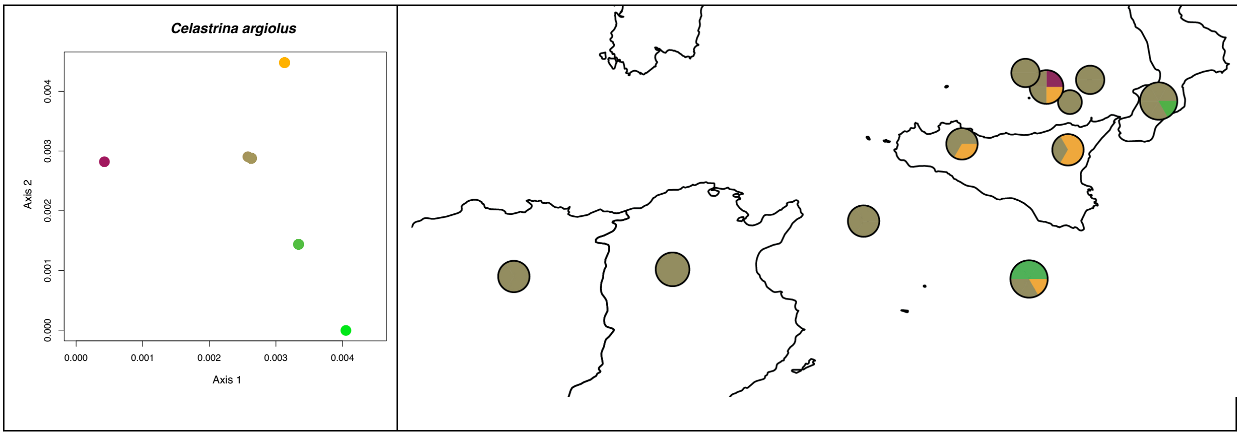

Figure 21 PCoA representation in the RGB color space and its projection on the map showing the patterns of differentiation in the slightly diversified haplotypes of *C. argiolus*. The apparently diversified populations from Sicily are due to the effect of haplotypes that differ only by a single mutation. The map used is freely available in the R package 'rworldxtra' at CRAN (https://cran.r-project.org/web/packages/rworldxtra/index.html).

Lampides boeticus (Linnaeus, 1767)

It is a widespread and generally common species. It is resident in the Mediterranean region, while its occurrence in most parts of Europe depends on regular seasonal migrations (Tolman & Lewington, 2008; Haahtela et al., 2011). According to its high migratory tendency, this species has low genetic variability across large geographic distances. However, Lohman et al. (2008) found two divergent clades (uncorrected pairwise distances ranging between 1.79 – 2.21%) belonging to specimens from Madagascar and Thailand, and northern Australia and the Moluccas, respectively. The study suggested that these lineages may have experienced accelerated differentiation due to infection with the bacterial endosymbiont *Wolbachia.* On Gozo Island, together with widespread Mediterranean haplotypes, we collected one specimen that shares barcode with a specimen from the Madagascar clade (Fig. 22). This suggests that some individuals can disperse for thousands of kilometers resulting in the sympatric occurrence of notably diverged haplotypes, albeit at low frequencies. Due to its dispersal capabilities, this species occurs on all the study islands (Balletto et al., 2005) and no spatial genetic pattern of variation is evident (Fig. 23).

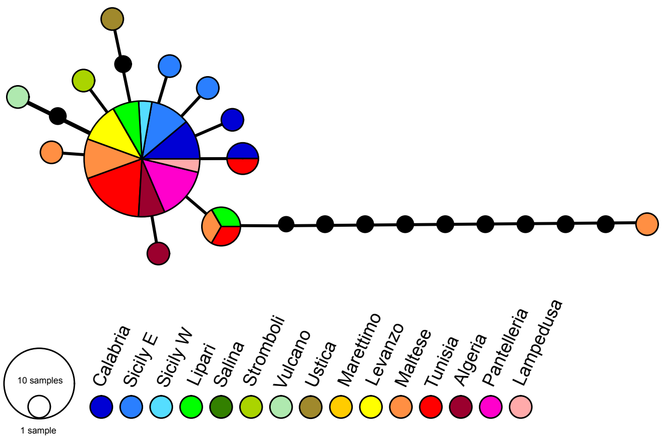

Figure 22 COI haplotype network for 41 specimens of *L. boeticus* from the study area, with the occurrence of a highly diverged specimen (10 mutations) sharing the same haplotype with a Malagasy population.

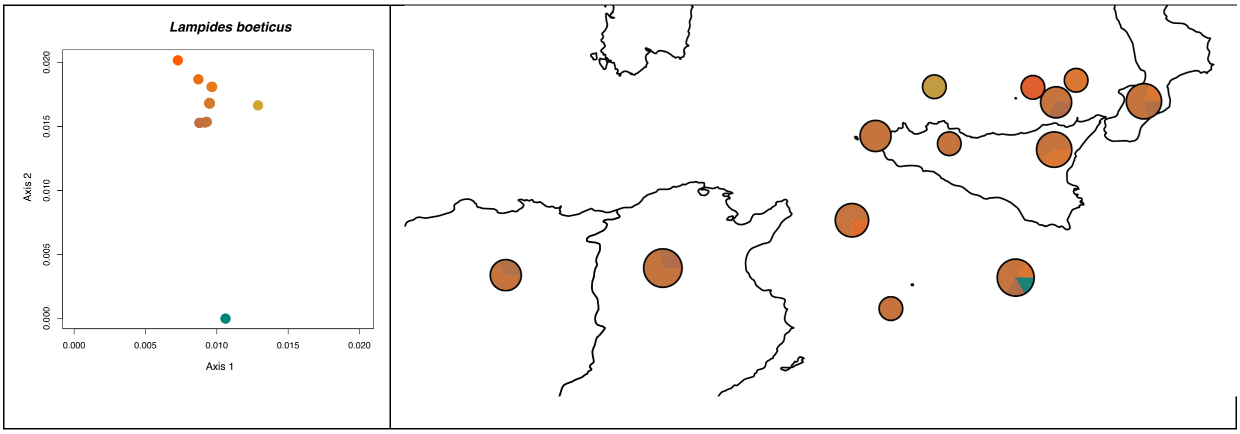

Figure 23 PCoA representation in the RGB color space and its projection on the map showing no pattern of differentiation in *L. boeticus*, except for a single specimen identical to those of a population from Madagascar. The map used is freely available in the R package 'rworldxtra' at CRAN (https://cran.r-project.org/web/packages/rworldxtra/index.html).

Leptotes pirithous (Linnaeus, 1767)

This is another well-known migrant and usually a common species. It is probably resident in the Mediterranean and performs migrations from north Africa to the rest of Europe (Haahtela et al., 2011; Tolman & Lewington, 2008). Similarly to *L. boeticus*, this species is present on all the studied islands (Balletto et al., 2005) and showed very low genetic variation over the study area (Fig. 24, 25).

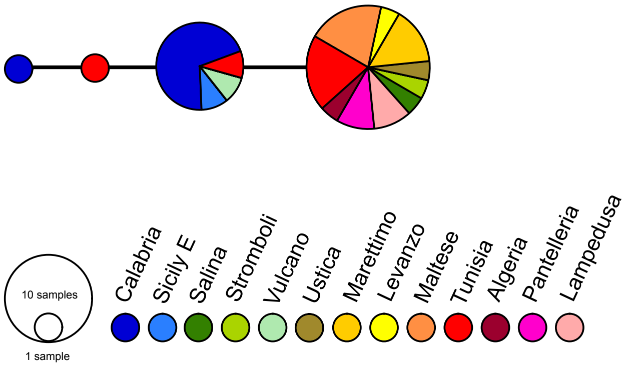

Figure 24 COI haplotype network for 32 specimens of *L. pirithous* from the study area.

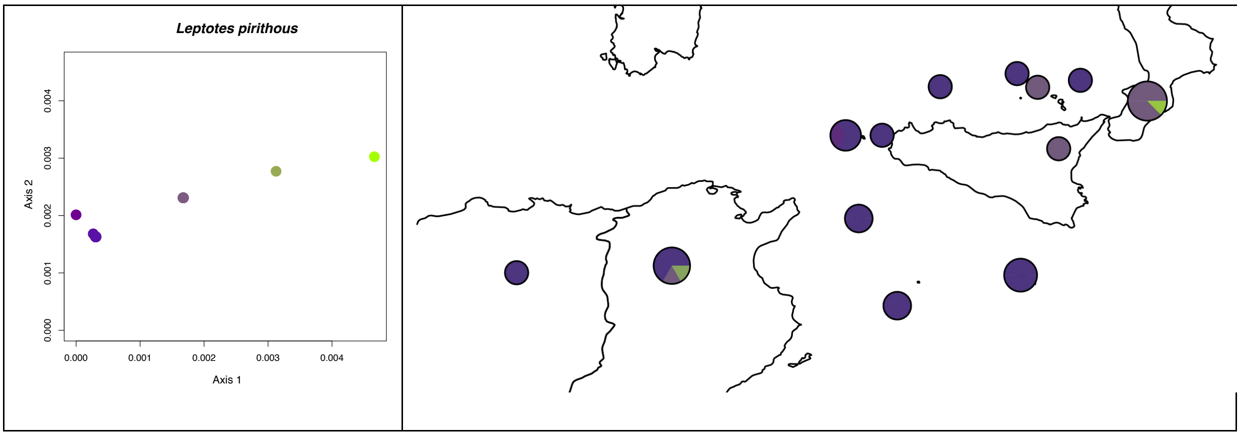

Figure 25 PCoA representation in the RGB color space and its projection on the map showing the patterns of differentiation in the low diversified haplotypes of *L. pirithous*. The apparent diversification of the population from Calabria can be due to the effect of the different frequencies of haplotypes that diverge by a single or two mutations only. The map used is freely available in the R package 'rworldxtra' at CRAN (https://cran.r-project.org/web/packages/rworldxtra/index.html).

Lycaena phlaeas (Linnaeus, 1761)

It is a cosmopolitan species and one of the few butterflies that reaches the most extreme northern latitudes (Haahtela et al., 2011). Although it is a rather common species on mainland, it was not reported from all the studied islands and records for Stromboli and Aegadian Islands are missing for the time being (Sammut, 1984; Aistleitner & Aistleitner, 2001; Biermann, 2005; Balletto et al., 2005; Seizmair, 2014). Its distribution was confirmed by our investigations, although in the Maltese islands it is currently considered extinct (Paul Sammut, personal observations). In the study area the species showed a peculiar genetic pattern with two main lineages separated by minimum three nucleotide substitutions (0.4%), the first occurring in Calabria, Maghreb, Lampedusa and Pantelleria, and the second in Sicily, Ustica and the Aeolian Islands (Fig. 26, 27). This indicates that, although the species is widely distributed, gene flow even across narrow sea barriers like the Messina strait is highly restricted (see Vodă et al., 2015 for possible hypotheses).

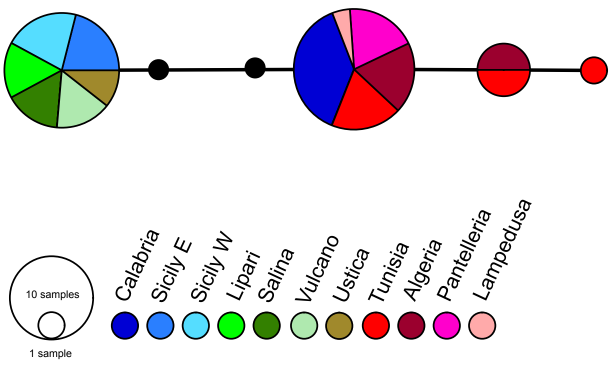

Figure 26 COI haplotype network for 45 specimens of *L. phlaeas* from the study area.

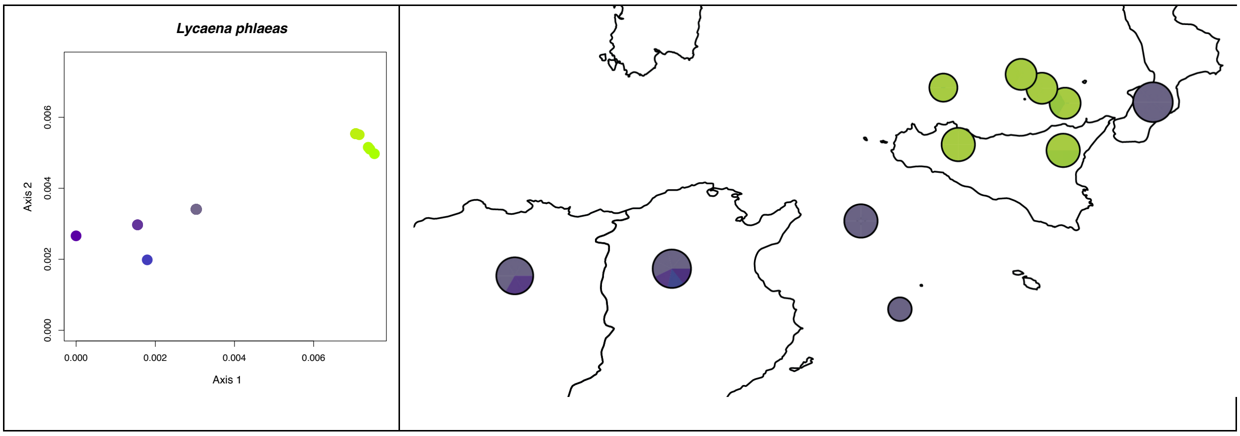

Figure 27 PCoA representation in the RGB color space and its projection on the map showing the patterns of genetic differentiation for *L. phlaeas*. Specimens in Sicily, Aeolian Islands and Ustica have a typical haplotype well differentiated from those occurring in Calabria, Maghreb, Lampedusa and Pantelleria. The map used is freely available in the R package 'rworldxtra' at CRAN (https://cran.r-project.org/web/packages/rworldxtra/index.html).

Pseudophilotes baton (Bergsträsser, 1779)

This species is endemic to western Europe and on islands it has been reported only from Corsica and Sicily (Balletto et al., 2005; Kudrna et al., 2011). On the 14th of June 2011 we collected two females on Vulcano, representing the first report for a small island. The genetic structure is very homogenous, with a single haplotype occurring in the study area (Fig. 28), suggesting that the population discovered on Vulcano represents a recent colonization event.

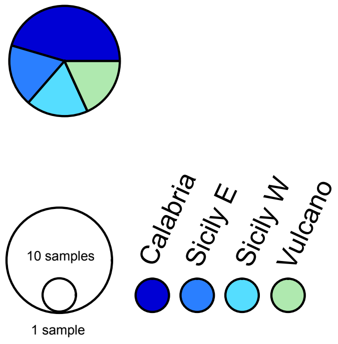

Figure 28 COI haplotype network for 11 specimens of *P. baton* from the study area.

Nymphalidae
Charaxes jasius (Linnaeus, 1767)

This species can be found along the Mediterranean coast and in several islands. However, among the circum-Sicilian islands it was only found in Lipari and Salina (Biermann, 2005). It is uncommon also in the surrounding areas. The few specimens we analyzed showed little diversification between the specimens from Maghreb and Europe (Fig. 29 and 30).

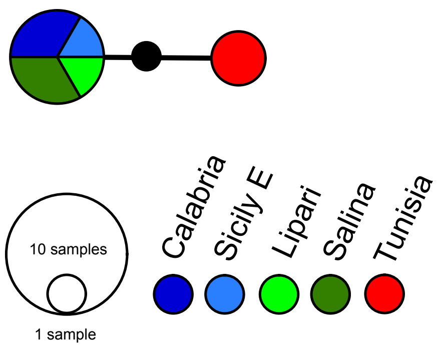

Figure 29 COI haplotype network for 8 specimens of *C. jasius* from the study area.

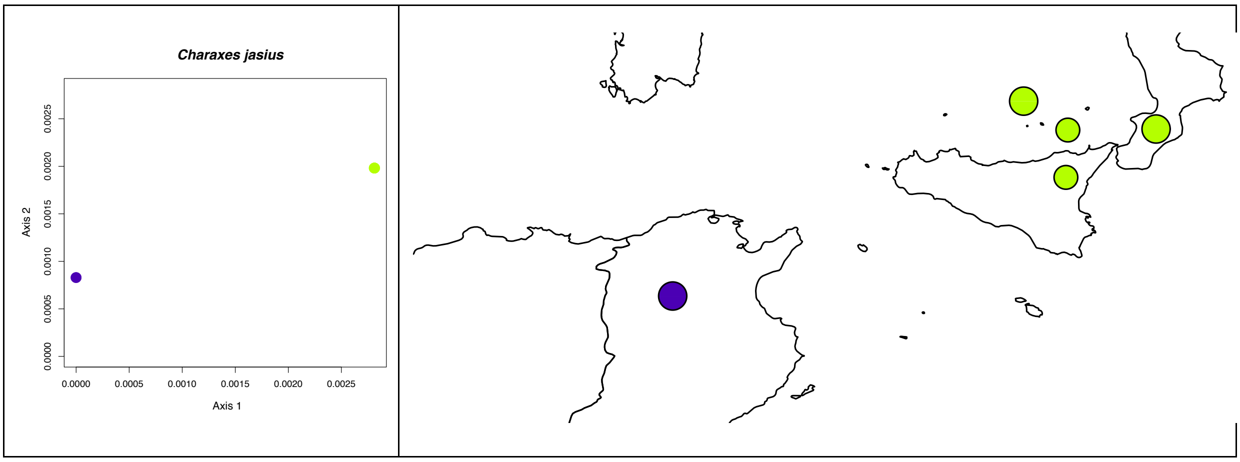

Figure 30 PCoA representation in the RGB color space and its projection on the map showing the patterns of genetic differentiation for *C. jasius*. There is a clear genetic differentiation between the Maghreb and Europe.

Vanessa atalanta (Linnaeus, 1758)

This species represents one of the most widespread and common butterflies in the European-Mediterranean region, including the islands (Balletto et al., 2005). It has been reported from all the circum-Sicilian islands studied here (Sammut, 1984; Balletto et al., 2005; Seizmair, 2014) and in recent years we confirmed its presence on most of them. *Vanessa atalanta* is known to perform directional migrations in Europe and in the Mediterranean (Stefanescu et al., 2003). Accordingly, no spatially structured genetic structure has been found over the study area (Fig. 31 and 32).

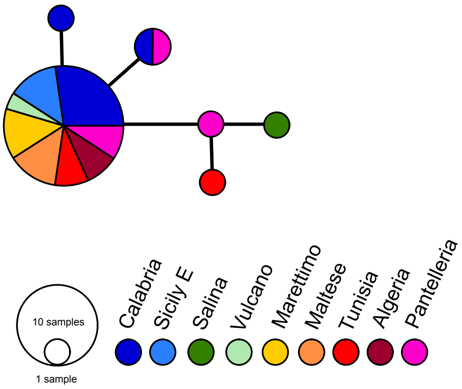

Figure 31 COI haplotype network for 28 specimens of *V. atalanta* from the study area.

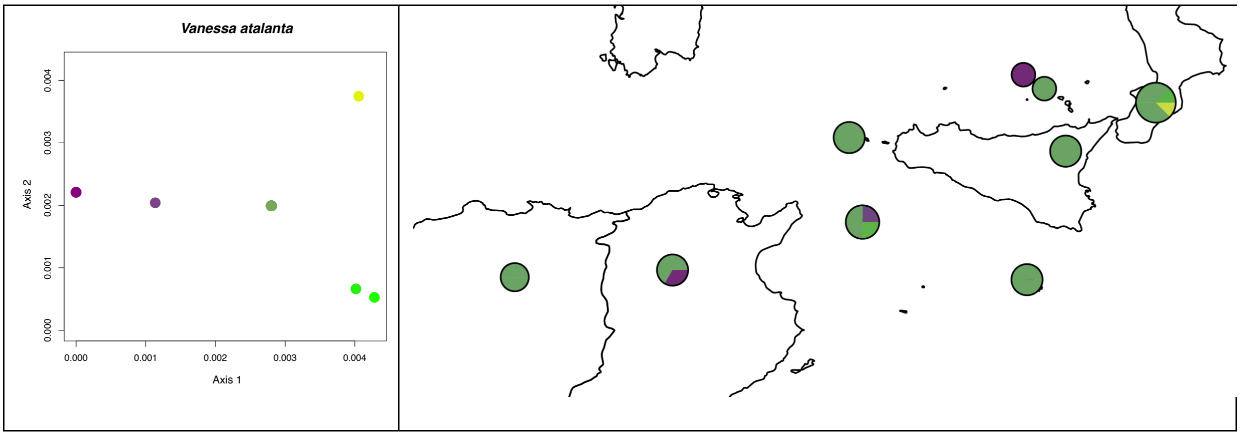

Figure 32 PCoA representation in the RGB color space and its projection on the map showing the absence of patterns of differentiation in the low diversified haplotypes of *V. atalanta*. The map used is freely available in the R package 'rworldxtra' at CRAN (https://cran.r-project.org/web/packages/rworldxtra/index.html).

Vanessa cardui (Linnaeus, 1758)

Vanessa cardui is one of the most widespread species not only in the Mediterranean area but all around the world, occurring on all the continents except for southern America and the polar areas. This species has continuous broods and migrates each year between Africa and Europe, sometimes in very large numbers (Stefanescu et al., 2013). The migratory cycle of this species is probably the best known among European butterflies. It involves six generations moving along a latitudinal shift of thousands of kilometers.

In spring, *Vanessa cardui* experience an annual poleward advance of the populations from Africa to Europe where the individuals reproduce and, two or three generations later, the offspring undergo an equatorward return migration in autumn (Stefanescu et al., 2013). On the basis of such premises it is not surprising that this species occurs all over the studied islands (Sammut, 1984; Aistleitner & Aistleitner, 2001; Biermann, 2005; Balletto et al., 2005; Seizmair, 2014) and that any genetic distances are small and not correlated with geography (Fig. 33, 34).

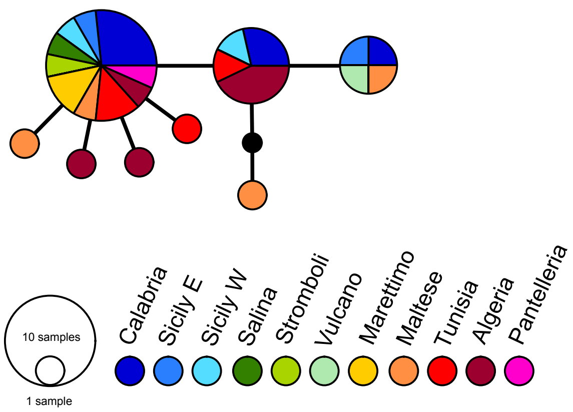

Figure 33 COI haplotype network for 31 specimens of *V. cardui* from the study area.

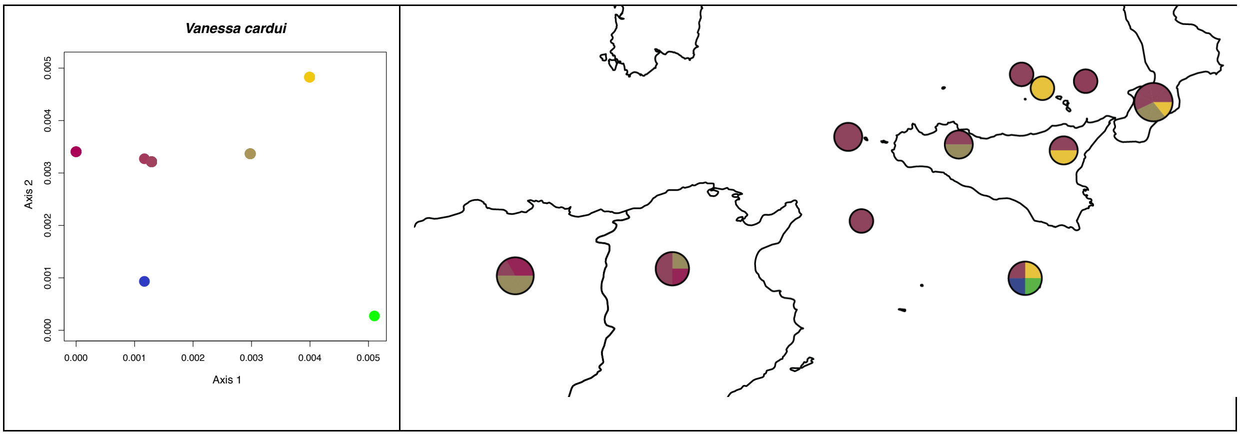

Figure 34 PCoA representation in the RGB color space and its projection on the map showing the absence of patterns of differentiation in the low diversified haplotypes of *V. cardui*. The map used is freely available in the R package 'rworldxtra' at CRAN (https://cran.r-project.org/web/packages/rworldxtra/index.html).

Hipparchia leighebi

The *Hipparchia* group is probably one of the most interesting complex of species in the Mediterranean and Atlantic islands. Based on major and minor variations in allozymes, genitalic and wing morphology, a series of species have been described, among which several insular endemics (Cesaroni et al., 1994; Balletto et al., 2005; Dapporto, 2010). Two main groups are represented by taxa with long and short male genitalia. In the western Mediterranean, *H. aristaeus* (from Sardinia, Corsica and Tuscan Archipelago), *H. algirica* (from Maghreb) and *H. blachieri* (from Sicily) have short genitalic structures, while *H. semele* (from the European mainland and Sicily), *H. leighebi* (from Aeolian islands) and *H. sbordoni* (from Ponza) show long genitalia. *H. neapolitana*, a taxon described from Campanian islands and surrounding mainland, has intermediate genitalia, but the populations from Calabria show a high degree of variation between large and short structures, therefore its taxonomic status is still debated (compare Cesaroni et al., 1994; Balletto et al., 2005; Dapporto, 2010). Even though this group has a fundamental contribution to the overall diversity of Mediterranean island butterflies, no study on mtDNA was carried out until now. Our results (Fig 35, 36) show that the populations from Maghreb (*H. algirica*), are separated by minimum 16 mutations (ca. 2.4% divergence) from populations in Sicily (*H. semele* and *H. blachieri*) and Calabria (with still uncertain status)The clear separation of the Maghrebian populations supports the specific status of *H. algirica*. The Sicilian specimens having both long (*H. semele*) and short (*H. blachieri*) genitalia are genetically very similar, with the exception of a single specimen of *H. blachieri* having a mtDNA haplotype that is more similar to *H. algirica* (7 mutations with respect to *H. algirica*) (Fig. 32). All the specimens from Calabria are genetically similar to the Sicilian ones and together they are almost identical to the European *H. semele* (not shown here but data are available for comparison in

Genbank). All the *H. leighebi* analyzed in this study formed a separate cluster, having about 0.6% minimum divergence with respect to *H. semele*. Interestingly, *H. semele* in Sicily is known to occur at mid-high altitudes and in cooler environments. This is not true for *H. leighebi*, one of the most common and widespread species on all the Aeolian islands, where it can be found also in very dry and hot sea level biotopes. The contrasting results of mtDNA, allozymes, morphology and habitat specialization, require more in depth studies to identify the mechanisms that produced the diversification in this group and ultimately, to recognize which taxa should be considered as species. One of the most interesting results for the circum-Sicilian islands was that, according to mtDNA, *H. leighebi* is the unique potentially endemic butterfly.

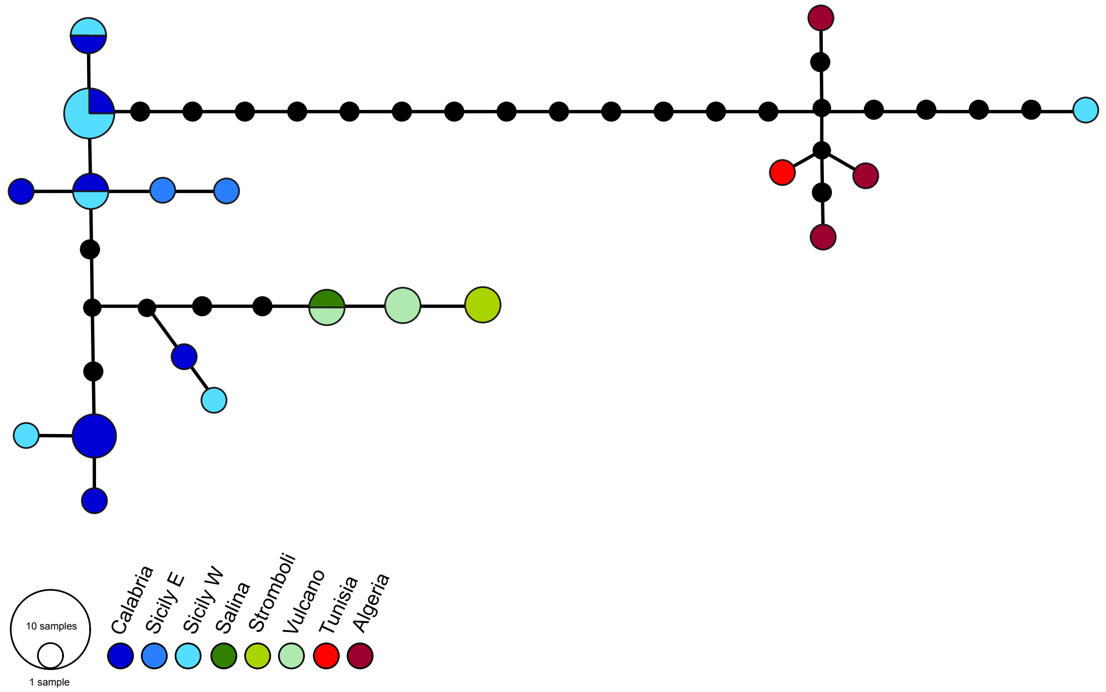

Figure 35 COI haplotype network for 29 specimens of *Hipparchia* from the study area.

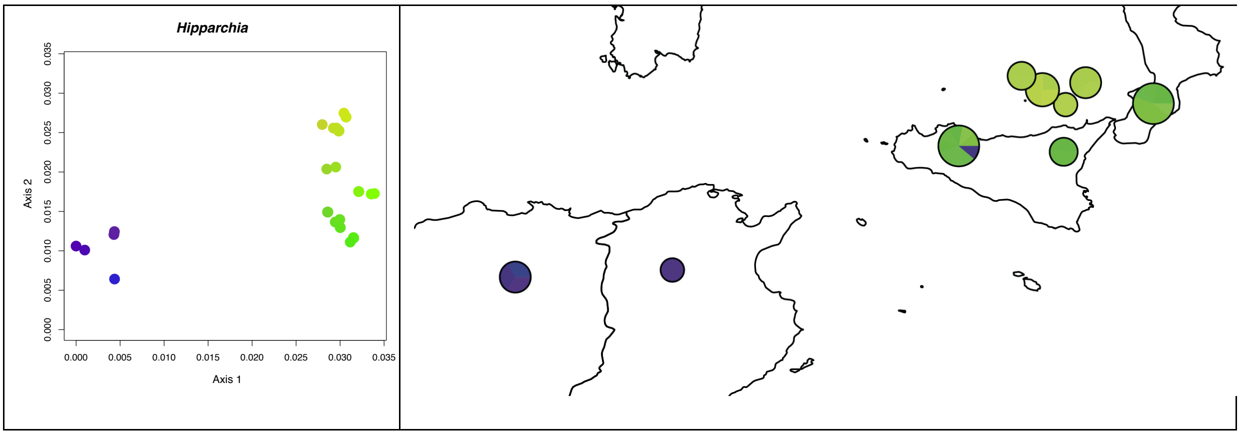

Figure 36 PCoA representation in the RGB color space and its projection on the map showing the existence of three main clades, north Africa, Aeolian Islands and Europe. The map used is freely available in the R package 'rworldxtra' at CRAN (https://cran.r-project.org/web/packages/rworldxtra/index.html).

Coenonympha pamphilus (Linnaeus, 1758)

Coenonympha pamphilus represents one of the most common and widespread butterflies in the Mediterranean. Studies on this species showed minor but constant differences in wing pattern and

genitalia shape that determined some authors to assign the populations occurring in in some parts of the western Mediterranean as belonging to a distinct species: *C. lyllus* (Boillat, 2002; Tarrier & Delacre, 2008; Dapporto et al., 2012). *Coenonympha pamphilus* was reported from most of Europe, including the islands of Sicily and Corsica, while *C. lyllus* from the Maghreb, southern Iberia, the Balearic Islands and Sardinia. This distinction was also confirmed by the morphology of the male genitalia, with intermediate areas in Sicily and southern Iberia (Dapporto et al., 2012).
Although often common on the mainland and on large islands, *Coenonympha* is absent from most small and isolated islands (Balletto et al., 2005; Dapporto et al., 2012), probably as a consequence of limited dispersal capability. Regarding the studied islands, they have been reported only from Levanzo (Dapporto et al., 2012) and the Maltese Islands (Sammut, 1984) where, in recent decades, they started to decline severely (Asher et al., 2001). Mitochondrial DNA recovered two clades, with the populations from the Maghreb (corresponding to taxon *lyllus*) being separated by at least 12 changes (ca. 1.8%) from those in Calabria, Sicily, Levanzo and Malta (Fig. 37, 38).

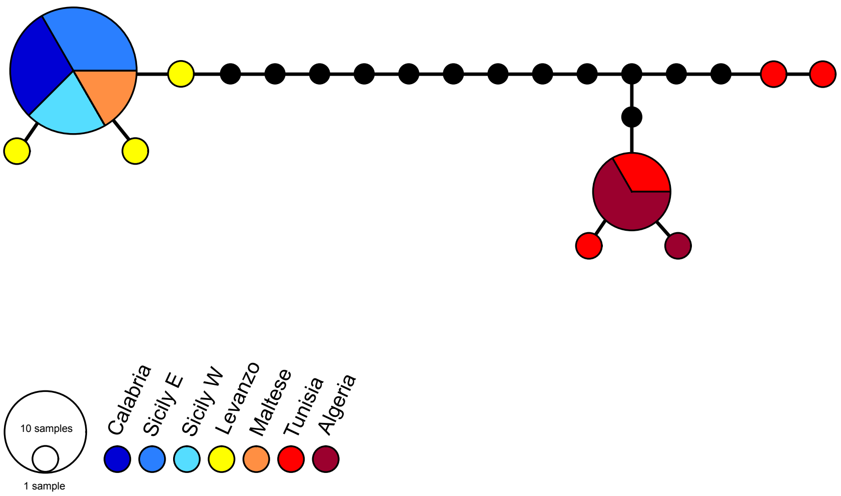

Figure 37 COI haplotype network for 37 specimens of *C. pamphilus/lyllus* from the study area.

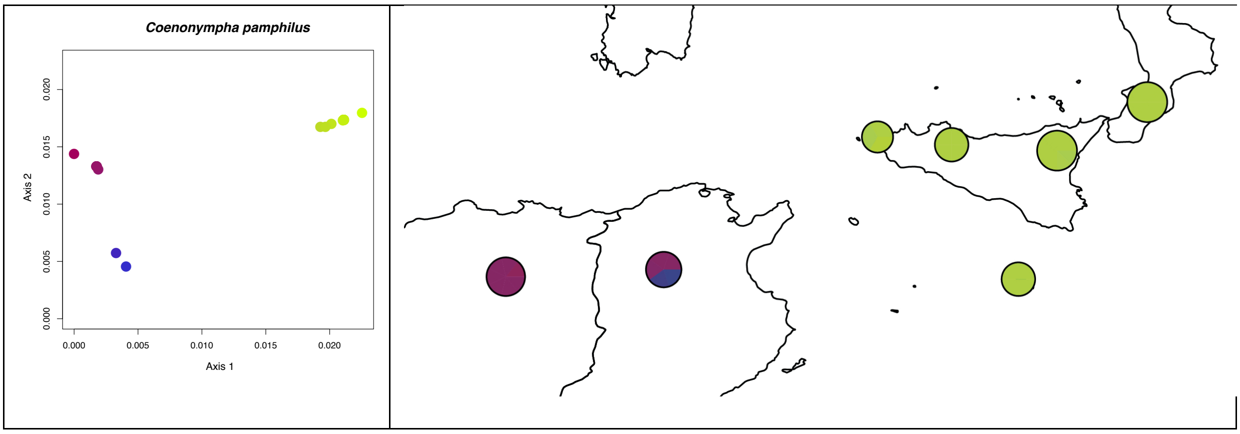

Figure 38 PCoA representation in the RGB color space and its projection on the map showing the patterns of differentiation for *C. pamphilus/lyllus*. As hypothesized by several authors, the populations from Maghreb (taxon *lyllus*) are genetically different from those in Calabria, Sicily, Levanzo and Malta (*C. pamphilus*). The map used is freely available in the R package 'rworldxtra' at CRAN (https://cran.r-project.org/web/packages/rworldxtra/index.html).

Pyronia cecilia (Vallantin, 1894)

Although this species was reported from several western Mediterranean islands, it is uncommon on the islands studied here. It is currently known only from the Aeolian islands of Vulcano, Lipari and Salina, and from Levanzo (Kudrna & Liegheb, 1988; Balletto et al., 2005; Dapporto et al., 2012),

although single very old reports are available for Ustica (Calcara, 1842). Analyses of the morphology of the male genitalia showed that the populations from Italy and Maghreb are well differentiated, while the specimens from Sicily are intermediate between the two areas (Dapporto et al., 2012). This pattern is perfectly reflected by the mtDNA, according to which there are two main clades separated by at least 12 nucleotide changes (ca. 1.8%, Fig. 39). The first clade occurs in Calabria, Sicily, the Aeolian Islands and Levanzo, and the second in the Maghreb and again in Sicily and Levanzo, with a prevalence of the Maghrebian lineage in western Sicily (Fig. 40). This interesting pattern suggests multiple colonization events from different sources on Sicily and Levanzo (the latter possibly sourced by Sicily itself).

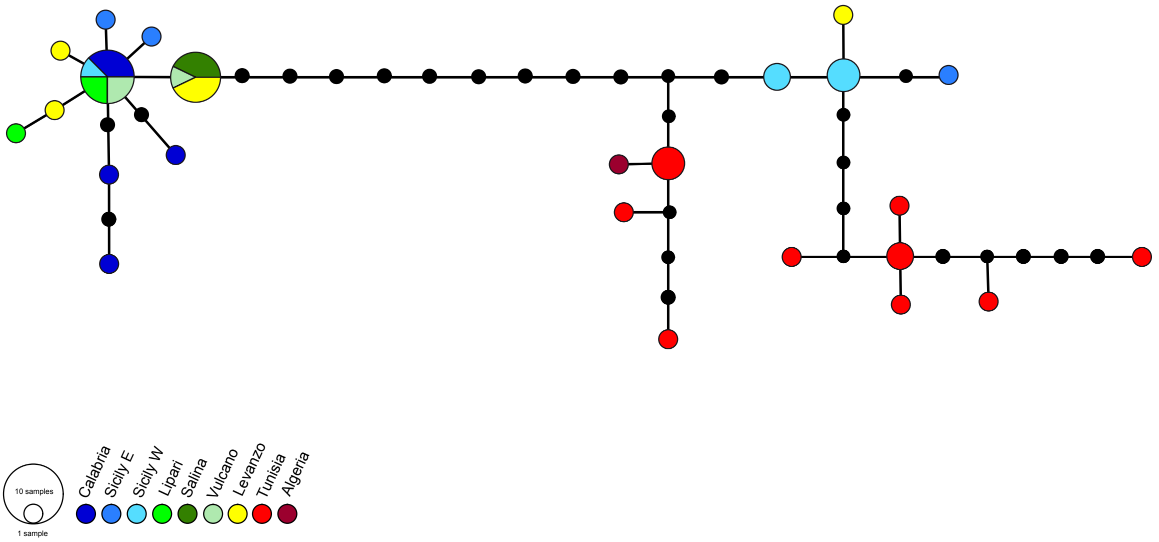

Figure 39 COI haplotype network for 43 specimens of *P. cecilia* from the study area.

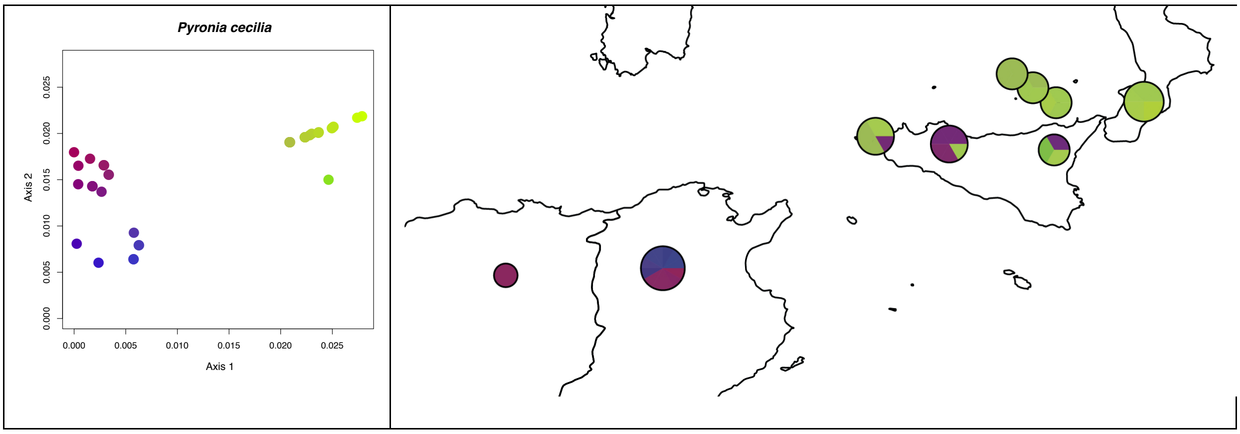

Figure 40 PCoA representation in the RGB color space and its projection on the map showing the patterns of differentiation of *P. cecilia*. There are two genetic lineages that co-occur only in Sicily and Levanzo. Western Sicily hosts mostly the Maghrebian lineage. The map used is freely available in the R package 'rworldxtra' at CRAN (https://cran.r-project.org/web/packages/rworldxtra/index.html).

Maniola jurtina (Linnaeus, 1758)

This species was reported from many western Mediterranean islands but it is uncommon on the islands studied here. It is known only from the Aeolian islands of Vulcano, Lipari and Salina and from Malta and Gozo (Sammut, 1984; Kudrna & Liegheb, 1988; Dapporto et al., 2012). In the last decades this species showed a severe decline, mostly on Malta (Asher et al., 2001; Sammut & Borg, 2008). Several studies reported the existence of two lineages in the Mediterranean, differentiated according

to allozymes, mitochondrial DNA and genitalia morphology (Thomson, 2001; Dapporto et al., 2014b). Other studies suggested more complex or unstructured patterns (Habel et al., 2009; Kreuzinger et al., 2014). Our analyses support the results of Dapporto et al. (2014b) by highlighting two clades, a western one occurring predominantly in Maghreb, Sicily and Gozo, and an eastern one occurring in Calabria and Sicily. Sicily and Calabria thus appears as the contact zones of the two lineages of this species. We also confirmed the existence of a separate lineage occurring only on the islands of Vulcano and Lipari (Fig. 41, 42). This endemic lineage was recovered by the PCoA and haplotype analyses as intermediate between the eastern and western lineages and it could represent a relict ancestral population.

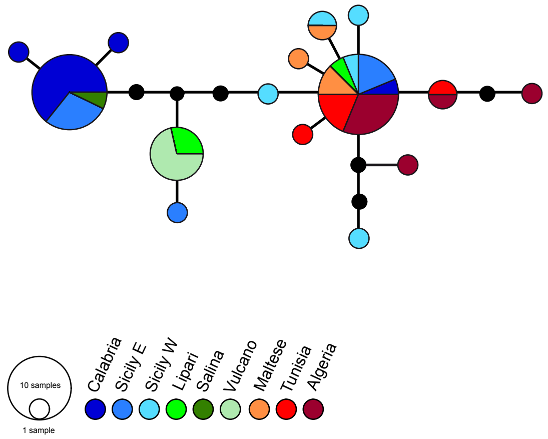

Figure 41 COI haplotype network for 51 specimens of *M. jurtina* from the study area.

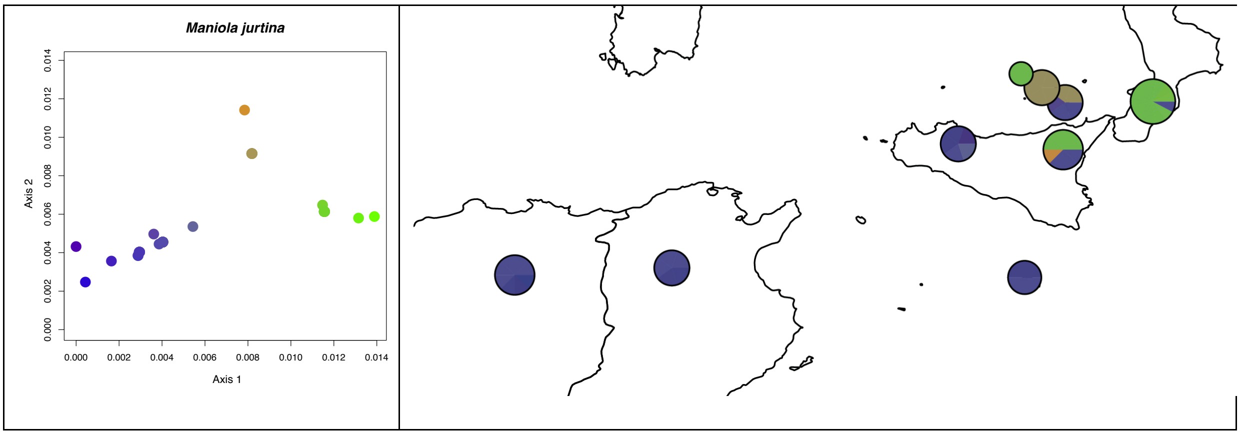

Figure 42 PCoA representation in the RGB color space and its projection on the map showing the patterns of differentiation in *M. jurtina.* The two lineages co-occur in eastern Sicily and the Aeolian Islands, while western Sicily and Malta only have the western lineage. The map used is freely available in the R package 'rworldxtra' at CRAN (https://cran.r-project.org/web/packages/rworldxtra/index.html).

Lasiommata megera (Linnaeus, 1767)

This species, together with its sister taxon *L. paramegaera* (Hübner, 1824), is widespread in the Mediterranean islands and sometimes represents one of the few resident species of smaller islands (Balletto et al., 2005; Dapporto et al., 2012). Excluding Linosa, *L. megera* has been reported from all the islands in the study area (Balletto et al., 2005). With the exception of Lampedusa (last report in 1997 according to Aistleitner & Aistleitner, 2001), in recent years (2009-2014) we confirmed the

above-mentioned distribution pattern. The genetic structure of this species over the Mediterranean was unknown until now. We found that two mitochondrial lineages exist in the study area, showing a minimum divergence of 1.1% (Fig. 43). One lineage occurs in Calabria, Sicily, the Aeolian Islands, Ustica, Levanzo, Marettimo and the Maltese Islands, and the second in Algeria, Tunisia and Pantelleria. The two lineages mutually exclude over the study area, including Pantelleria, which is located at a rather similar distance from Sicily and Tunisia (Fig. 44). Previous morphological studies of the male genitalia did not find conspicuous differences between these two lineages (Dapporto et al., 2012).

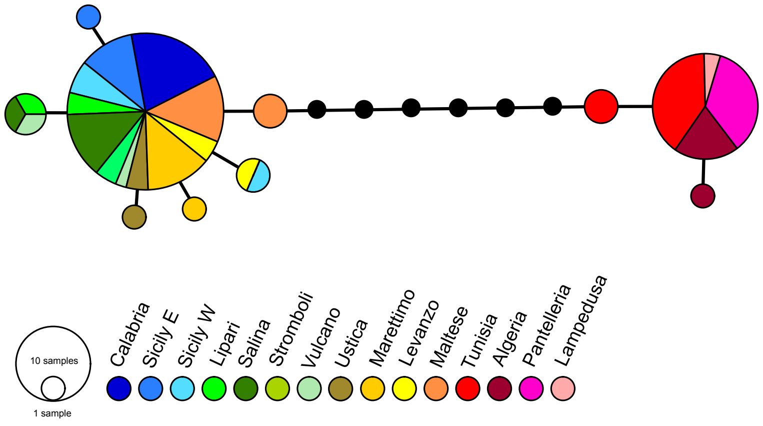

Figure 43 COI haplotype network for 77 specimens of *L. megera* from the study area.

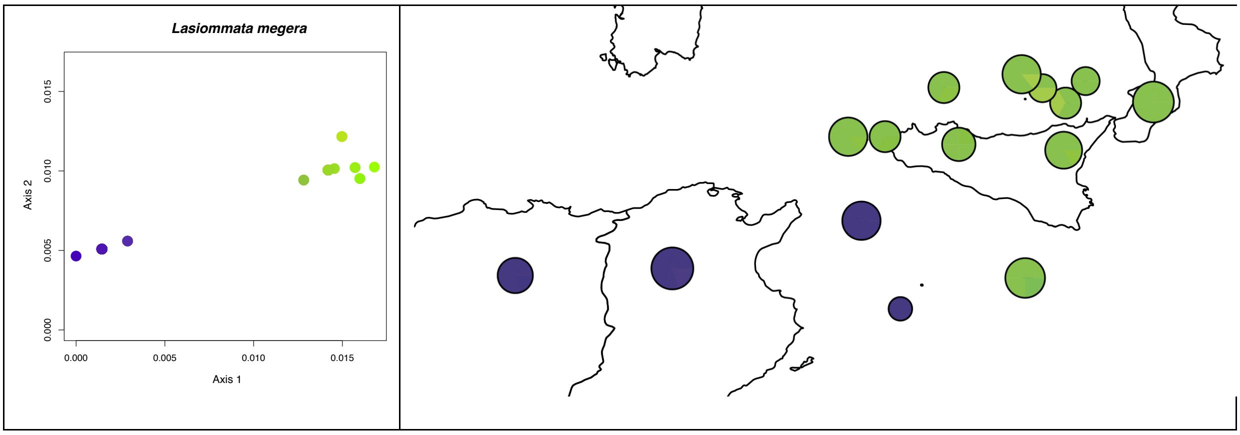

Figure 44 PCoA representation in the RGB color space and its projection on the map showing a strong differentiation between the lineages of *L. megera* in Sicily-Italian Peninsula and Tunisia and Algeria. Pantelleria host typical north African populations, while the rest of the circum-Sicilian islands have only the Sicilian lineage, which indicates a mutually exclusive pattern of the two genetic lineages. The map used is freely available in the R package 'rworldxtra' at CRAN (https://cran.r-project.org/web/packages/rworldxtra/index.html).

Pararge aegeria (Linnaeus, 1758)

This species has been reported from many western Mediterranean islands but it is uncommon on the islands studied here. It has been reported from almost all Aeolian Islands (Stromboli, Vulcano, Salina, Lipari) (Biermann, 2005) and from Malta and Gozo (Sammut, 1984; Kudrna & Liegheb, 1988), although in recent years this species showed severe declines on the latter islands (Paul Sammut personal observation). Previous studies (Weingartner et al., 2006; Habel et al., 2012) found two well-differentiated lineages within *P. aegeria* from Europe and Maghreb according to both mitochondrial and nuclear markers. We confirm these findings (minimum divergence of 1.1%, Fig. 45, 46) and also show that all three specimens analyzed from Gozo Island belong to the European lineage.

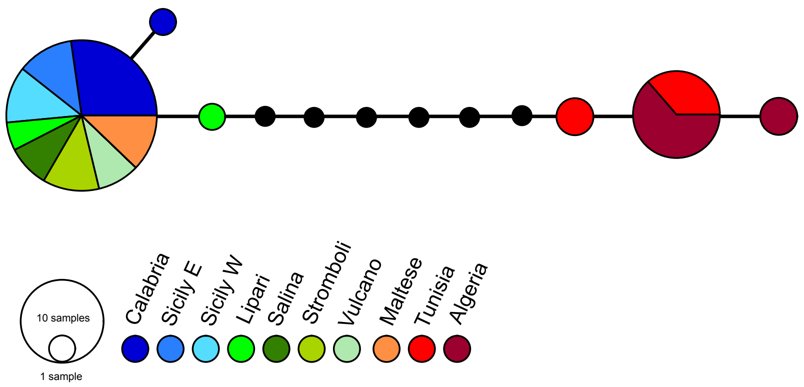

Figure 45 COI haplotype network for 50 specimens of *P. aegeria* from the study area.

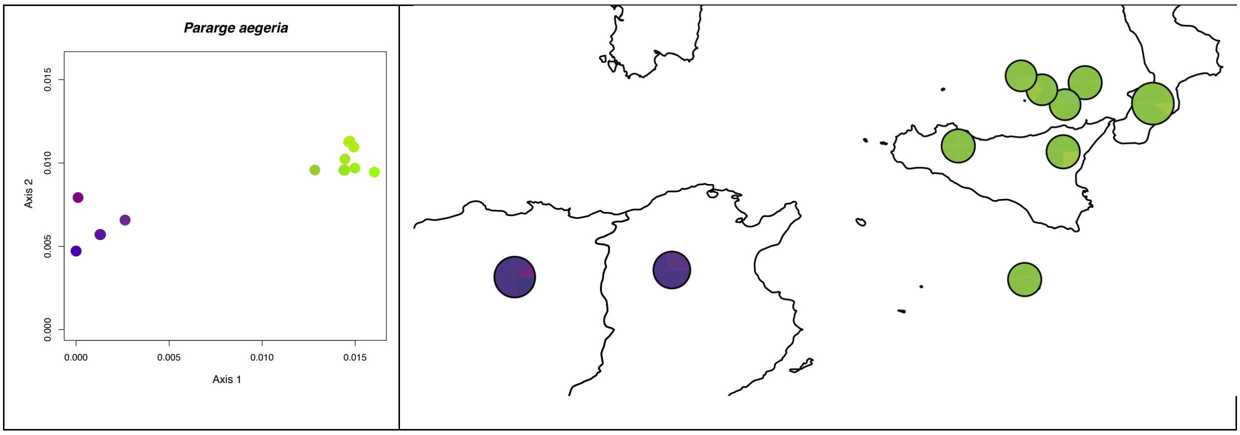

Figure 46 PCoA representation in the RGB color space and its projection on the map showing a strong differentiation between the *P. aegeria* lineages in Sicily-Italian Peninsula and Tunisia and Algeria. The Aeolian and Maltese Islands showed typical Sicilian populations. The map used is freely available in the R package 'rworldxtra' at CRAN (https://cran.r-project.org/web/packages/rworldxtra/index.html).

Danaus chrysippus (Linnaeus, 1758)

Danaus chrysippus is one of the most widespread subtropical butterflies occurring over most of the African continent and in the southern regions of Asia. Thanks to its marked dispersal and migration abilities it often reaches Europe, but it usually does not reproduce there (Tolman & Levington, 2008). In the study area the species has been occasionally recorded from Malta and from many localities in Sicily (Balletto et al., 2005) but locally breeding populations are only reported from Tunisia, Algeria, Lampedusa (Pisciotta et al., 2008) and from one locality in eastern Sicily. Thus, we considered that among the circum-Sicilian islands this species occurred only on Lampedusa. This decision was supported by the fact that, during eight years of collections in Sicily and circum-Sicilian islands, we only collected this species in Lampedusa. The haplotype network indicates the existence of differentiated haplotypes but, as expected for a highly dispersive species, their distribution shows no geographic pattern (Fig 47, 48). Moreover, the presence of different haplotypes on Lampedusa indicates that the island has been reached several times by female specimens.

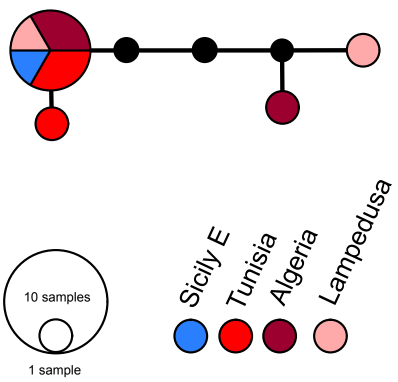

Figure 47 COI haplotype network for 9 specimens of *D. chrysippus* from the study area.

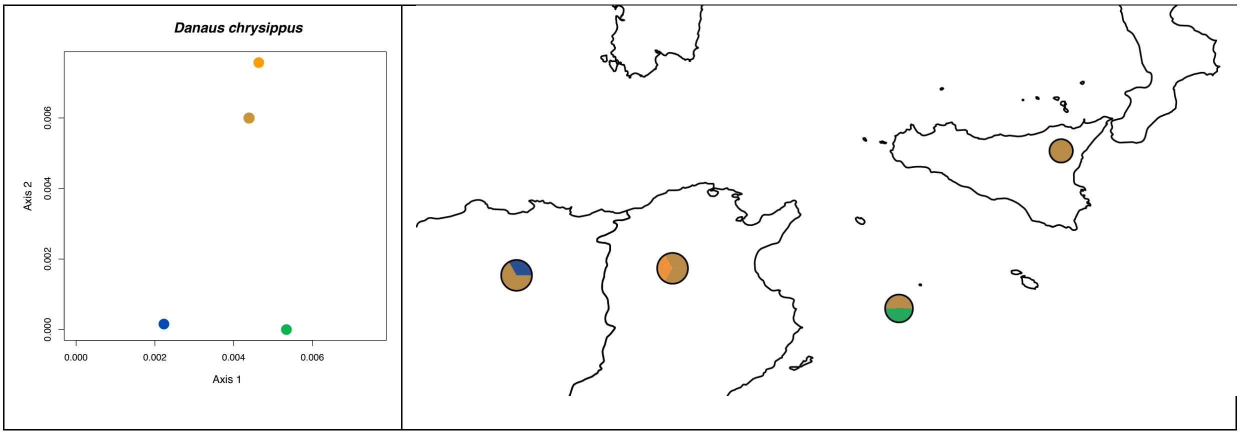

Figure 48 PCoA representation in the RGB color space and its projection on the map showing no pattern of differentiation in *D. chrysippus*. The map used is freely available in the R package 'rworldxtra' at CRAN (https://cran.r-project.org/web/packages/rworldxtra/index.html).

Hesperiidae
Carcharodus alceae (Esper, 1780)

It is considered that, unlike other skippers, this species can fly over large distances and colonize new territories quite easily (Haahtela et al., 2011). On the study islands it has been found only on Levanzo, Lipari and Sicily. Based on wing morphology it is inseparable from *C. tripolinus,* but they display pronounced differences in male genitalia and DNA (more than 2.5% COI divergence) (Dinca et al. 2015). *C. tripolinus* replaces *C. alceae* in north Africa and they have a parapatric distribution in southern Iberian Peninsula. The genetic structure of populations of *C. alceae* from Sicily, Lipari and Levanzo on one side and from Calabria on the other side, showed a constant separation of at least ten changes (1.5%, Fig. 49, 50). This suggests that, despite reports of high dispersal capability, some still unknown mechanisms are maintaining the separation between the populations from Sicily, Lipari and Levanzo and those of the Italian peninsula, and that these factors may also be at the basis of its low frequency on islands.

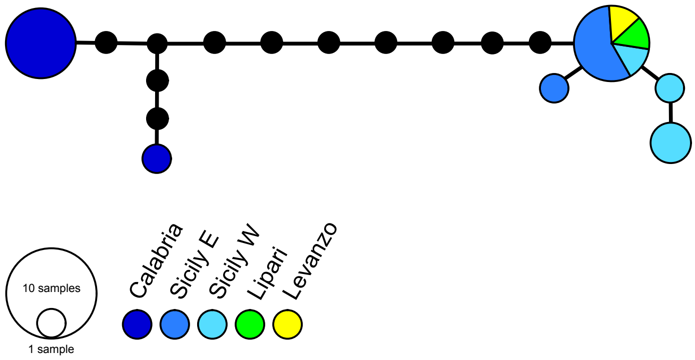

Figure 49 COI haplotype network for 18 specimens of *C. alceae* from the study area.

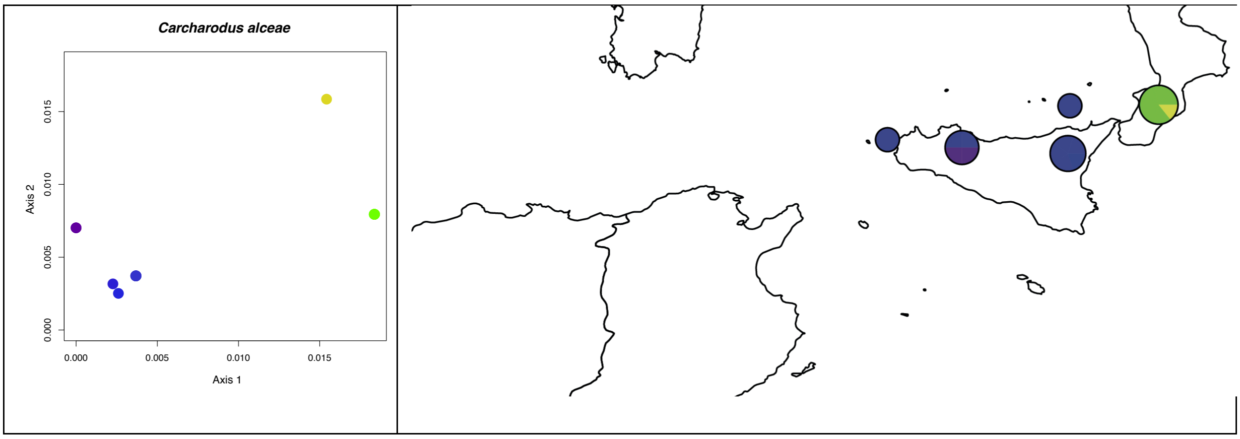

Figure 50 PCoA representation in the RGB color space and its projection on the map showing the differentiation between lineages of *C. alceae* occurring in Sicily and in the Italian Peninsula. These lineages mutually exclude over the Messina strait. Lipari and Levanzo host typical Sicilian populations. The map used is freely available in the R package 'rworldxtra' at CRAN (https://cran.r-project.org/web/packages/rworldxtra/index.html).

Gegenes nostrodamus (Fabricius, 1793)

In the western Mediterranean, this butterfly is typically associated to coastal areas. Among the studied islands it has been reported only from Sicily (where we confirm its presence), Lampedusa and Lipari (Balletto et al., 2005). It should however be noted that the high external resemblance with *G. pumilio* could explain the lack of records for some islands. In Sicily both species are known to co-exist. Only two haplotypes differentiated by one mutation have been found, suggesting that this species possesses high dispersal capabilities (Fig. 51, 52).

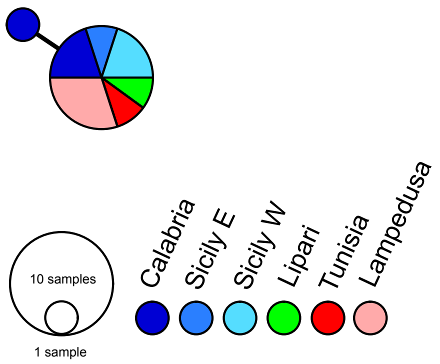

Figure 51 COI haplotype network for 11 specimens of *G. nostrodamus* from the study area.

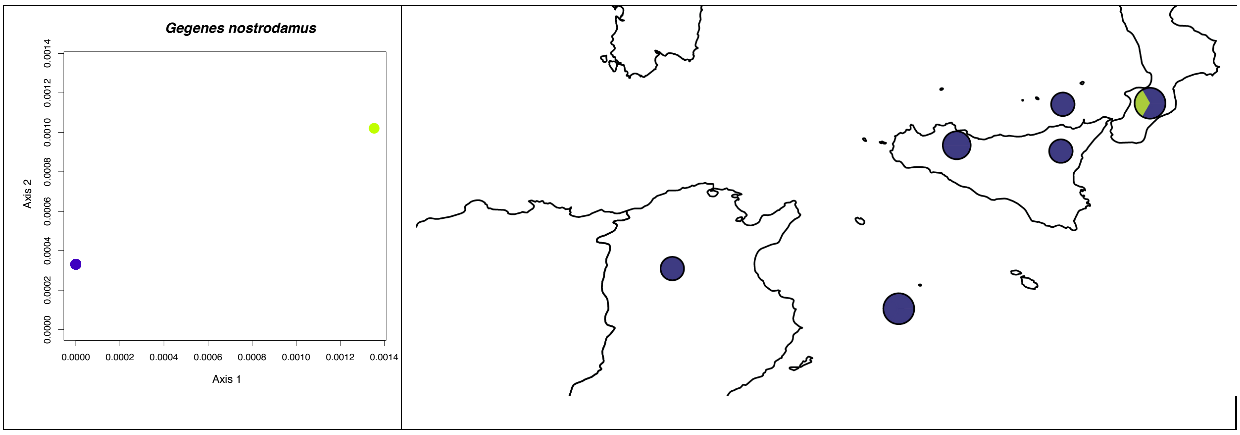

Figure 52 PCoA representation in the RGB color space and its projection on the map showing the differentiation between lineages of *G. nostrodamus*.The map used is freely available in the R package 'rworldxtra' at CRAN (https://cran.r-project.org/web/packages/rworldxtra/index.html).

Gegenes pumilio (Hoffmannsegg, 1804)

The external morphology of this butterfly is very similar to *G. nostrodamus*, but G. *pumilio* is slightly smaller in size and apparently more common on the circum-Sicilian islands. It was reported from Pantelleria, the Maltese Islands, Ustica, Lipari and Stromboli (Sammut, 1984; Balletto et al., 2005; Seizmair, 2014). We also found it on Levanzo, Salina and Ustica. Like the above-mentioned species, *G. pumilio* showed very little genetic structure over the study area (Fig. 53, 54).

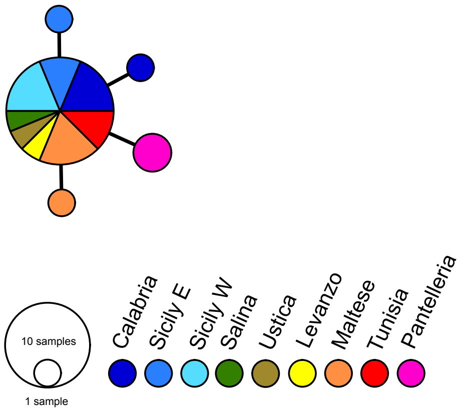

Figure 53 COI haplotype network for 21 specimens of *G. pumilio* from the study area.

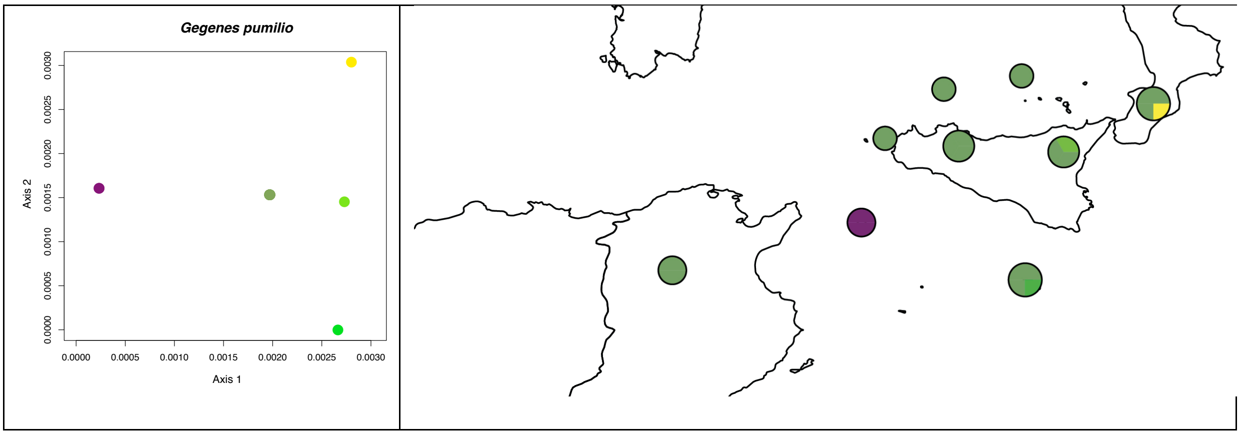

Figure 54 PCoA representation in the RGB color space and its projection on the map showing no pattern of differentiation in *G. pumilio*. The map used is freely available in the R package 'rworldxtra' at CRAN (https://cran.r-project.org/web/packages/rworldxtra/index.html).

THE ISLANDS

In this section we describe the butterfly community found on each island studied by combining the genetic information (haplotype networks and PCoA projections) and the patterns obtained by the frequency-genetic diversification graphs (see main paper for their description).

Vulcano

With an area of 21.2 km^2 Vulcano is the sixth largest circum-Sicilian island. It is an active volcano, emerged relatively recent (130 ky). The island is characterized by violent eruptions separated by long periods of quiescence: in the last 6000 years six major eruptions took place. The last eruption that occurred between 1888 and 1890 has been well documented. In the first phases solid rocks were expelled over a maximum distance of 7 km that reached even the neighboring island of Lipari. The explosive eruptions that followed had pyroclastic fragments of viscous magma that dispersed over most part of the island. Due to the last eruption 5m of material accumulated on the summit and destroyed a large part of the vegetation on the island (Calanchi et al., 2007).

The butterfly fauna of Vulcano comprises 23 species, only one species less than the richest circum-Sicilian island of Lipari. The overall genetic structure of the island resembles that of Sicily and some species are particularly interesting. Up to now, out of all the circum-Sicilian islands *P.s baton* has only been reported from Vulcano. Together with the other Aeolian islands, it also hosts *H. leighebi*, the only island endemic in the study area. The high species richness of this island, and of all the Aeolian islands as well, is probably due to a combination of varied habitats (due to the presence of steep slopes along the volcanic cones) and of a relatively well preserved environment.

All the widespread species occur on this island and among the eight uncommon ones, five species showed high genetic variation in the study area (*P. cecilia, M. jurtina, H. leighebi, P. aegeria*). Beside the endemic *H. leighebi*, Vulcano and Lipari host a particular haplotype of *M. jurtina*, intermediate between the eastern and the western lineages. Among the species showing genetic differentiation, *L. phlaeas* belonged to the typical Sicilian lineage, while all the specimens of *P. cecilia* and of the non-endemic lineage of *M. jurtina* were typical of the Italian Peninsula. These lineages also occur in eastern Sicily, but mixed with the African ones. The presence of pure Italian lineages on these islands may represent remnants of the ancient populations established before a possible recent natural invasion of lineages from southwestern Mediterranean areas to eastern Sicily. Ultimately, this implies that the populations of these species on Vulcano and Lipari are not connected with those from Sicily, and thus their conservation status should be monitored and immediate actions should be taken in case of any detected decline.

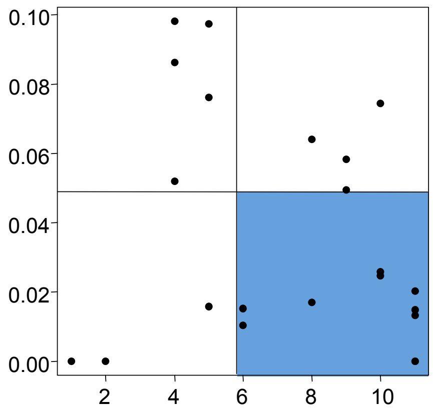

Figure 55 The genetic differentiation (Dst) frequency diagram for Vulcano island.

No quadrant in the genetic diversity-frequency diagram was significantly empty (Fig. 55), confirming the existence of a varied butterfly fauna despite the catastrophic events produced by volcanic activity. This can be due to the high proximity to Lipari, which probably functioned as a reservoir of diversity from where species re-colonized Vulcano after the habitats were restored. Only *P. baton* has not yet been reported from Lipari, although it occurs on Vulcano.

Lipari

After Malta and Pantelleria, Lipari is the largest among the circum-Sicilian islands (37.6 km^2). It represents the emerged part of a large volcanic complex higher than 1600 m, of which 602 m are above sea level. The first eruptions are dated 220 ky ago and the last effusion occurred in historical times, about 1.3 ky ago (Calanchi et al., 2007). Among the Aeolian Islands, Lipari is the most densely populated by humans with a stable population of about 11,000, but reaching 20,000 during the summer period. This resulted in a strong impact on the natural vegetation as a consequence of agriculture and, in the coastal areas, because of touristic activities. Nevertheless, with 25 recorded species, Lipari is the most butterfly-rich circum-Sicilian island. The frequency-genetic structure diagram (Fig. 56) revealed that only four of the 27 studied species are missing from this island (*D. chryshippus*, *P. baton*, *G. cleopatra*, *P. daplidice*). They mostly represent rare but genetically undifferentiated taxa, and therefore the corresponding sector is significantly empty.

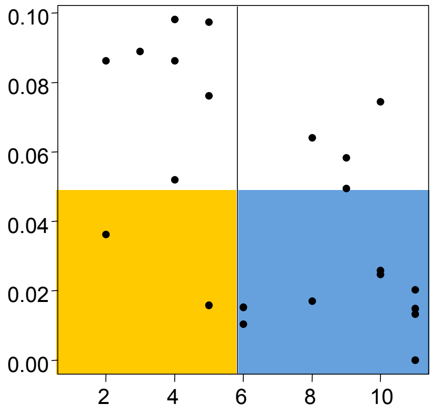

Figure 56 The genetic differentiation (Dst) -Frequency diagram for Lipari island.

The genetic characterization of the butterfly community in Lipari is very similar to that on Vulcano, with most species having typical Sicilian haplotypes, also when they are differentiated between the Italian Peninsula and Sicily (*C. alceae* and *L. phlaeas*). Similarly to Vulcano, the exceptions are represented by *H. leighebi*, *P. cecilia* and *M. jurtina*. The populations occurring on these two islands are probably rich in number and genetic characteristics because they are highly interconnected and

probably they support each other in case of potential declines on one of the islands. A similar mechanism is probably at play between Malta and Gozo (see below). This could reduce the extinction rate, move the equilibrium point to richer values and allow particularly long term survival of endemic (*H. leighebi*) and relict (*M. jurtina*, *P. cecilia*) elements.

Salina

With an area of 26.4 km^2, Salina is the second largest island in the Aeolian archipelago. It was formed by two twin volcanic cones having a maximum altitude of 962 m. The island probably emerged 450 ky ago and the last volcanic activity is dated around 13 ky ago (Calanchi et al., 2007). Salina is characterized by extensive maquis-forest vegetation dominated by *Erica arborea* and *Arbutus unedo*. The flora of this island is probably the best preserved among the Aeolian Islands, and probably also among all the circum-Sicilian islands. The butterfly fauna is varied with many diversified (eight) and rare (six) species, making this island very similar to Lipari and Vulcano, albeit slightly poorer (20 species, Fig. 57).

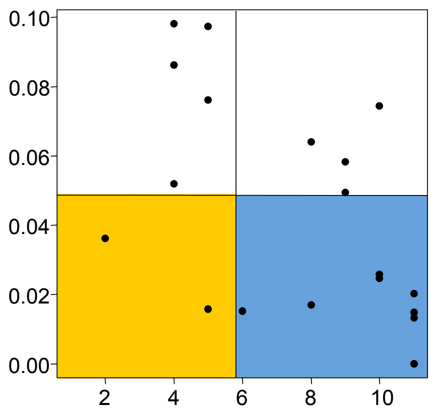

Figure 57 The genetic differentiation (Dst) -Frequency diagram for Salina island.

Besides *H. leighebi*, common on all the Aeolian Islands, Salina hosts the Sicilian lineage of *L. phlaeas*, and also the lineage of *P. cecilia* from the Italian mainland, suggesting a complex colonization history. *M. jurtina* is less frequent than on Vulcano and Lipari, and the only specimen we analyzed belongs to the typical African lineage, not recorded so far from other Aeolian islands. However, more specimens are needed to clearly delineate the genetic structure of this species in the archipelago. *Charaxes jasius* represents another interesting element of the fauna of Salina. Strictly linked to its unique host plant *Arbutus unedo*, widespread on this island, this species is common on Salina, but less frequent on Lipari and it is difficult to observe in Sicily (Balletto et al., 2005). We never recorded any individual from Sicily during years of research. *Charaxes jasius*, together with *H. leighebi*, *L. phlaeas* and *P. cecilia* provide most of the uniqueness of the butterfly diversity in this island.

Stromboli

With an area of 12.6 km^2, Stromboli is the fifth largest island in the Aeolian Archipelago. The island emerged about 100 ky ago and represents the apical 924 m of a large volcano that is 3000 m high. The volcano is still active with constant minor eruptions and common major eruptions. However, the low viscosity of the magma and the general reduced volume of erupted materials make the major eruptions much less destructive than those occurring on Vulcano (Calanchi et al., 2007).
Unlike the other Aeolian islands, Stromboli is slightly closer to the Italian Peninsula (54 km) than to Sicily (56 km). It is difficult however to know to which of the two sources the butterfly community is

more related since, probably due to the relatively high isolation, most of the butterfly species occurring on this island are represented by widespread and genetically undifferentiated taxa. Of the 15 species reported on Stromboli, almost all are widespread and 11 show reduced genetic variation (Fig. 58). Among the diversified species, *H. leighebi* has the same genetic structure as the populations occurring on other Aeolian islands, and *P. machaon*, *P. edusa* and *L. megera* show no diversification between Sicily and Calabria. The presence of *P. machaon* on this island needs confirmation since it has been recorded only once in 2000 (Biermann, 2005).

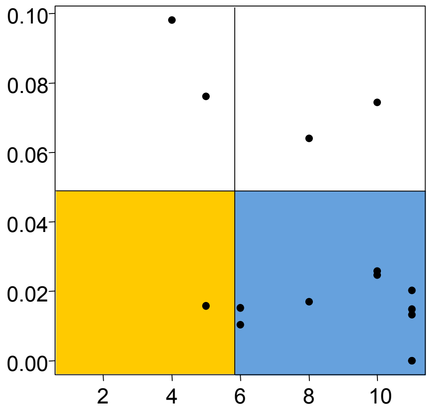

Figure 58 The genetic differentiation (Dst) -Frequency diagram for Stromboli island

The main characteristic of this island is the lack of most uncommon species, compared to other Aeolian islands. The fact that it has never been in contact with surrounding areas and its relatively pronounced isolation, represent a first indication (also confirmed for Ustica, Marettimo and Pantelleria) that most differentiated species, including those that are generalist and common on the mainland, have limited occurrence on islands presumably because of reduced dispersal capabilities.

Ustica

Ustica is a small island (8 km^2) representing the remnant apex of an ancient volcano emerged about 350 ky ago and which ended its activity about 85 ky ago. Ustica is also relatively isolated, being located 67 km away from Sicily. Accordingly, its butterfly fauna is not particularly rich and comprises the one species more (16) as the similarly isolated Stromboli (Fig. 59). However, five species are not shared between these islands resulting in a rather different faunistic composition. The small area and higher degree of isolation filtered even more the butterfly fauna toward highly generalist and dispersive species. As a result, both quadrants comprising rare species are significantly empty.

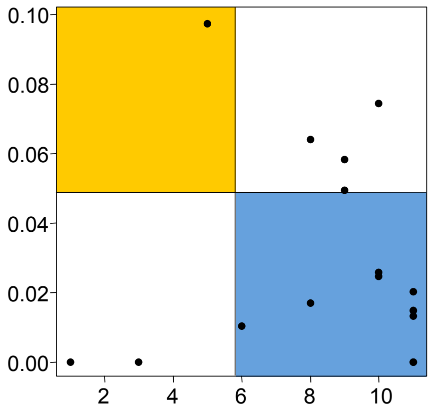

Figure 59 The genetic differentiation (Dst) -Frequency diagram for Ustica island.

There are however a few species genetically structured on Ustica (*P. machaon*, *L. phlaeas*, *A. agestis*, *L. megera*) with typical Sicilian haplotypes and without any evidence of endemic or relict haplotypes. Ustica represents another relevant example of the strong effect of species filtering based on dispersal behavior and capabilities compared to a neutral frequency-dependent hypothesis.

Levanzo

With an area of 5.6 km^2, Levanzo is the second smallest island studied, after Linosa. It is composed of calcareous rocks and is currently 12.5 km away from Sicily. However, because it is surrounded by very shallow sea, Levanzo has been connected to Sicily for a long time during colder periods. The current and historical proximity to Sicily made Levanzo relatively rich in species, not only in highly vagrant and widespread ones, but also in several infrequent taxa on islands (*C. alceae*, *P. cecilia*, *C. pamphilus*, *Pieris mannii*, *G. cleopatra*). The first three species mentioned above, together with *P. celina* and *L. megera*, also showed high genetic variation over the study area. As a result, the butterfly community appears to be varied, with no significantly empty sectors in the frequency-genetic diversity diagram (Fig. 60).

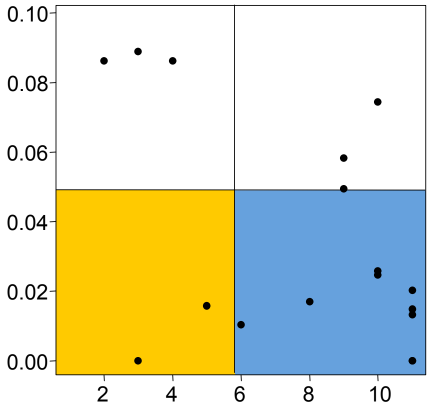

Figure 60 The genetic differentiation (Dst) -Frequency diagram for Levanzo island

According to the present day and historical proximity with Sicily, all the species showing genetic diversity had typical Sicilian lineages. Moreover, most species have several haplotypes on the island, suggesting multiple colonization events from Sicily (see the haplotype networks). In particular, the Maghrebian and Italian clades of *P. cecilia* coexist on Levanzo, and also in western Sicily. An exception is *C. pamphilus,* with three different haplotypes, separated by a single mutation that, in the study region, have been found only here. Although a more thorough sampling would probably expose these haplotypes in other areas as well, the existence of uncommon halpotypes on Levanzo could suggest that this species, among the less vagile in our dataset, could be weakly connected with the Sicilian populations. This implies that, following a potential local extinction, *C. pamphilus* may not be able to easily recolonize Levanzo. Therefore, our data indicate that this population should represent a conservation priority on Levanzo.

Marettimo

Marettimo is the westernmost Aegadian island with an area of 12.3 km^2 and a maximum altitude of 690 m. It is currently located about 30 km away from the Sicilian coast but it lies only a few km outside the large sector of the shallow Sicilian shelf from where Levanzo and Favignana emerge. For this reason, Marettimo was probably not connected to Sicily during glacial periods, but it got very near Sicily during cold phases. The island is not densely populated and it is mostly covered by well-preserved Mediterranean maquis vegetation.
There are only a few faunistic reports for butterflies on Marettimo and combining them with our recent collection data, we obtained a list of only eight species. Even though more intense research on this island may help in reporting new species, it is evident that Marettimo has a community of

butterflies extremely skewed toward very common and highly dispersive species. All the species known from Marettimo have been recorded for 10 or 11 islands in our dataset (Fig. 61).

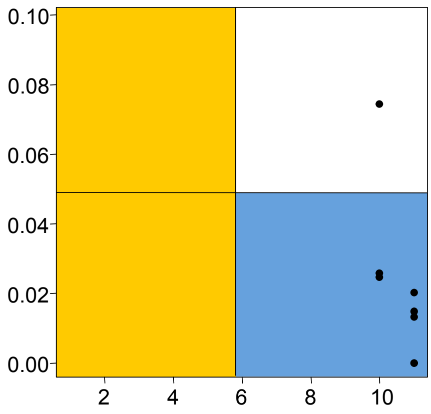

Figure 61 The genetic differentiation (Dst) -Frequency diagram for Marettimo island.

These species do not show any regional genetic structure with the exception of *L. megera,* which in Marettimo is similar to populations from Sicily and Calabria. However, this island has an undefined butterfly community, providing further clues that some common species occurring on mainland are impeded to occur on islands probably as a result of dispersal and habitat requirement constraints.

Pantelleria

Pantelleria is an ancient volcano that was active between 114 and 45 ky ago. With an area of 83 km^2, this island represents the second largest circum-Sicilian island after Malta. The relatively high maximum altitude (836 m) is benefic for a varied environment. The highest natural structure, Monte Guardia, is covered by pinewoods and maquis and, together with traditionally managed agricultural areas, it offers potential habitats for many species. Nevertheless, the butterfly fauna of Pantelleria is rather poor and undiversified, and most of the 14 species recorded on the island are widespread (Fig. 62). The only rare species reported is *P. daplidice*, but since the last collection dates back to 1986 (Romano and Romano, 1995) and the specimens are too old for a routine DNA analysis, for the moment it is not clear if they are indeed *P. daplidice* or its sibling species *P. edusa*. Our research group organized three field expeditions of six days between 2012 and 2014 but could not confirm the presence of this species on the island. *Papilio machaon* was also reported in the past from Pantelleria (Balletto et al., 2005) but its presence has not been confirmed in recent years.

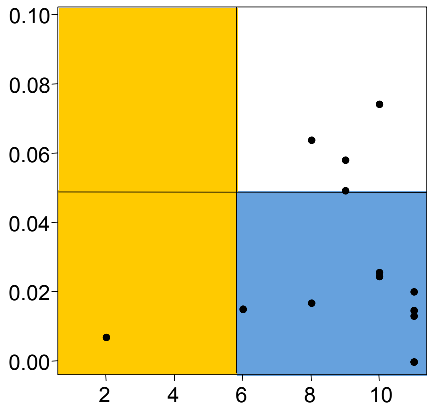

Figure 62 The genetic differentiation (Dst) -Frequency diagram for Pantelleria island.

Although the butterfly fauna of this island appears undifferentiated at the species level, three of the widespread species examined (*L. phlaeas*, *P. celina* and *L. megera*) had lineages similar to those occurring in the Maghreb, suggesting a preferential direction of colonization from this source. This pattern was quite unexpected since, although the island is currently located farther from Sicily (about 100 km) than from Tunisia (approx. 70 km), during the last glacial maximum it was located very close to Sicily. Wind direction (Fig. 63) may be involved in the observed pattern. Winds are predominantly blowing tangentially with respect to the two African and Sicilian sources (from NW to SE), but there is a minor, although still evident, contribution of winds blowing from the SW, which could potentially favor colonization from Tunisia. Winds blowing from NE are extremely uncommon and tend to show lower speed.

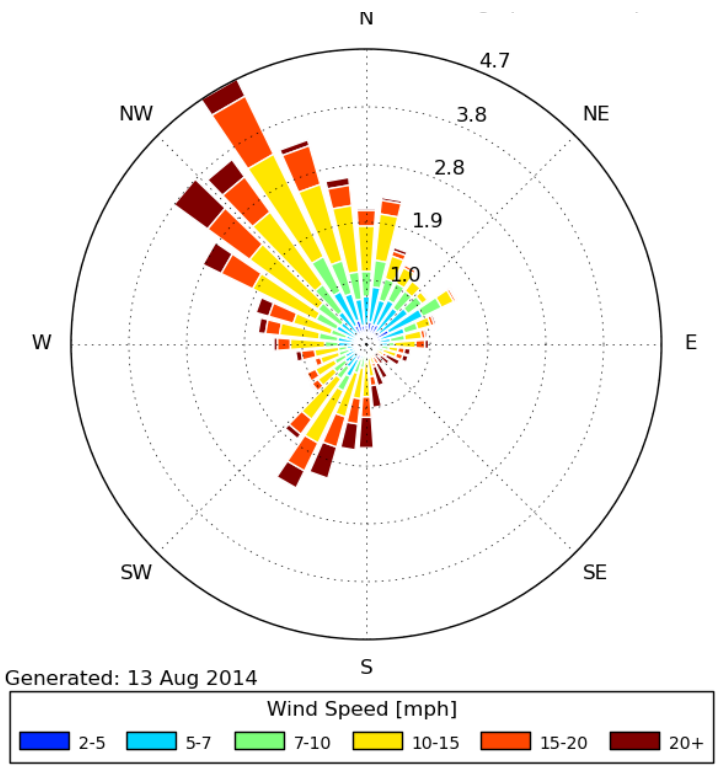

Figure 63 A wind rose for the airport of Pantelleria
(from http://mesonet.agron.iastate.edu/sites/windrose.phtml?station=LICG&network=IT__ASOS).

Whichever the mechanisms determining the observed pattern, the butterfly diversity of the island is characterized by the typical north African genetic structure of these species. Noteworthy, *P. daplidice* (identified without genetic examination) and *P. machaon*, recorded in the past, also show diverging populations between Maghreb and Sicily. Confirming the presence of *Pontia* sp. and *P. machaon* and analyzing their genetic structure would be of great importance to validate the genetic similarity of this island with Tunisia. More generally, thanks to its location at a relatively similar distance between the two most diversified areas of the Mediterranean (north Africa and Sicily-Italian Peninsula), to the proximity of Sicily during glacial periods and to the pattern of wind direction, this island represents a perfect natural laboratory to study the mechanisms that produce and maintain mutually exclusive patterns of distribution. Any species on this island (not only butterflies) can potentially contribute to the understanding of this yet largely unknown phenomenon. It is likely that mutually exclusive patterns are maintained by "founder takes all" mechanisms reinforced by density dependent processes (competition, reproductive interference, etc.), and that the established populations are the barrier against natural and human introduced propagules, which could change the original diversity pattern. For this reason, the current populations of *P. celina*, *L. phlaeas* and *L. megera* (and of *Pontia* sp. and *P. machaon* if confirmed) should be the priority for the conservation of the butterfly diversity on Pantelleria.

Malta

The Maltese archipelago comprises three main islands: Malta (246 km^2), Gozo (67 km^2) and Comino (3.5 km^2). These islands are disposed in line, separated by two narrow sea straits, 1.5 and 0.5 km long, and can be considered as a single unit for highly dispersing organisms like butterflies. The islands are mostly formed by tertiary sedimentary limestone. The maximum altitude is rather low (253 m) compared to the other, even smaller, circum-Sicilian islands of volcanic origin. Currently, the Maltese islands are highly isolated from any surrounding mainland, compared to most of the circum-Sicilian islands. The Sicilian coast is 92 km north of the islands, while Tunisia is about 300 km west. However, Malta lies on the same shallow submarine shelf as Sicily, with sea depths lower than -100 m. This configuration allowed the connection of Malta with Sicily during the glacial maxima, when the sea levels dropped, even more than -100 m in some regions. The study of butterfly richness suggests that some of the species that colonized the area during the Pleistocene connection persisted on the Maltese islands until recent times.

Another peculiar characteristic is the high population density, with more than 400,000 people currently living on these islands. The Maltese islands have been intensively cultivated for a long time and the strong anthropogenic imprint on the wild vegetation dates from early Neolithic (Carrol et al., 2012). In particular, forested areas are almost absent. However, if on one side the human impact considerably reduced the habitats of many species, on the other side, there were many butterfly admirers that gathered a large set of records for the butterflies occurring on these islands. According to van Swaay et al. (2010) there are 18 breeding species on the Maltese island but, in time, many occasional reports of single individuals for several other species have become available. These species are widespread in the southern Mediterranean (*Hipparchia* sp., *Polygonia c-album*, *Callophrys rubi*), or they are typical of the European mainland (*Pieris napi*, *Aglais io*, *Aglais urticae*, *Polygonia egea*, *Pyronia tithonus*), or typical of north Africa (*Euchloe belemia*, *Danaus chrysippus*, *Zizeeria karsandra*). Since in several cases, specimens have been photographed or collected, there are few doubts that single vagrant individuals can reach these islands from different sources without establishing breeding populations. This suggests that also the species that are already occurring on these islands can have common genetic exchanges with both continental areas (southern Europe and northern Africa). Nevertheless, when a species showed different genetic lineages between Africa and Europe, the populations of the Maltese islands (and of other circum-Sicilian islands as well) tended to host only one of them, indicating that some still unknown mechanisms operate to establish and maintain patterns of mutual exclusion (Vodă et al., 2015).

Finally, it is not easy to identify which species of the Maltese fauna represent the relicts from the Pleistocene connection, but some hypotheses can be made. The widespread species should be excluded, since they are able to disperse to islands in any moment by crossing the sea straits. Among the uncommon Maltese species, some do not occur on any circum-Sicilian island except on the Aeolian (*M. jurtina, P. aegeria, A. agestis*, the last also occurring on Ustica) or on Levanzo (*C. pamphilus*). All these species show two well-differentiated lineages between north Africa and Europe, and the specimens collected on the Maltese islands have the same lineages occurring in Sicily, as it was expected because of their Pleistocene connection (we did not analyze the mtDNA of *A. agestis* from Malta but two old specimens have been reliably identified by analyzing the male genitalia). Importantly, all these species are severely declining on the Maltese islands, probably as a response to the increasing human impact (Paul Sammut, personal observation). This set of uncommon species, absent in the surrounding islands and with a strong genetic diversification, is a key feature of the butterfly diversity of Maltese islands (Fig. 64). For this reasons, they should be considered as fundamental for conservation priorities in these islands, even though they represent common species over most Mediterranean mainland.

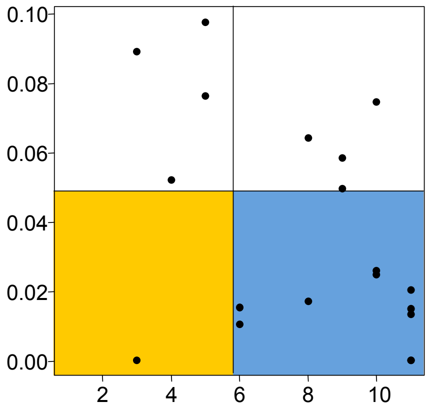

Figure 64 The genetic differentiation (Dst) -Frequency diagram for the Maltese islands.

Linosa

With an area of 5.5km^2, Linosa is the smallest island in our study area. It is a volcanic island emerged during the Pleistocene and the last eruption is dated around 2.5 ky ago. Besides being very small, Linosa is also very isolated, 165 km away from Tunisia and 160 km from Sicily. Combining data on species reported in literature (*P. rapae*, *L. phlaeas*, *P. celina*, *V. atalanta*, *V. cardui*; Balletto et al., 2005) and our won observations (*C. croceus*, *L. boeticus*, *V. cardui*), only seven widespread species are currently known from this island (Fig. 65). Although results are currently unavailable, the analysis of the mtDNA of *L. phlaeas* and *P. celina*, reported in 1956 (Zavattari, 1960), could reveal if the island is mainly influenced by Sicilian or Tunisian propagules.

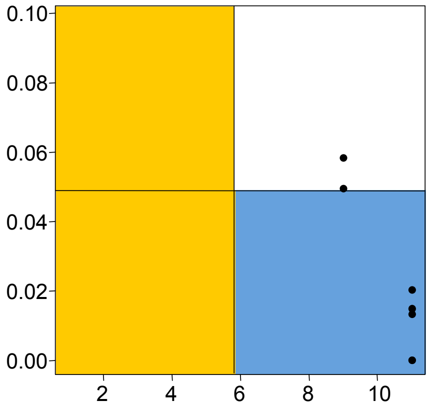

Figure 65 The genetic differentiation (Dst) -Frequency diagram for Linosa island.

Lampedusa

Lampedusa is a calcareous island located on the African continental shelf. The island has an area of 20.2 km^2 and, unlike similarly sized volcanic islands, it is almost completely flat and has a maximum altitude of only 133 m. The African shelf in this area is characterized by shallow sea and the maximum depth separating Lampedusa from Tunisia is about 120 m. The island is currently highly isolated, 113 km away from Tunisia, 150 km from Malta and 205 km from Sicily. As showed for most of the other relatively highly isolated islands, Lampedusa has a butterfly fauna mostly composed by common and poorly diversified species, reflected by the fact that the sector of rare and diversified species is significantly empty (Fig. 66).

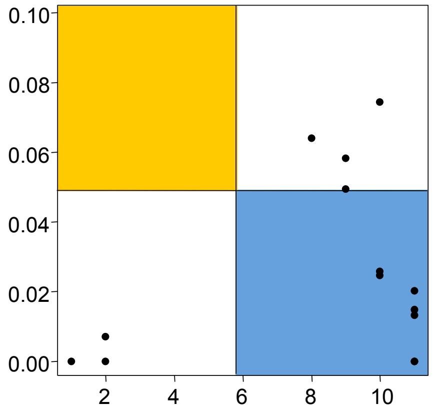

Figure 66 The genetic differentiation (Dst) -Frequency diagram for Lampedusa island.

However, there is a set of species that characterize its diversity. Of all the circum-Sicilian islands, *Danaus chrysippus* is known to reproduce only here. This is also the only island where we found *P. daplidice* (on Pantelleria the old record must be confirmed by DNA data) and from where *Z. karsandra* has been recorded (although we did not examine its DNA). There is also a single record of the Maghreb-Arabic element *Azanus ubaldus*, but its presence on the island must be confirmed. All these species represent north African elements (*P. daplidice* also occurs in Sardinia, Corsica, France and Iberia) and usually do not reproduce in Sicily and the Italian Peninsula (with the exception of a colony of *D. chrysippus*; Cucuzza, 1998). The examination of the intraspecific genetic variation confirmed this pattern and all the species showing differentiated clades between Sicily-Italian Peninsula and Africa (*P. machaon*, *L. phlaeas*, *P. celina*) had typical north African haplotypes on Lampedusa. *Lasiommata megera* also shows distinct haplotypes between the Maghreb and Europe, but the last recorded specimen on this island was collected in 1997 and we were unable to find it. This island is the most isolated in our dataset and therefore should have a butterfly fauna composed predominantly of common and undifferentiated species (as it happens in Marettimo, Ustica, Pantelleria and Stromboli). Unexpectedly, its butterfly fauna comprises a large fraction of north African species and lineages. The wind patterns do not help to explain this pattern, since most of the winds blow from directions where landmasses are very far (Fig. 67).

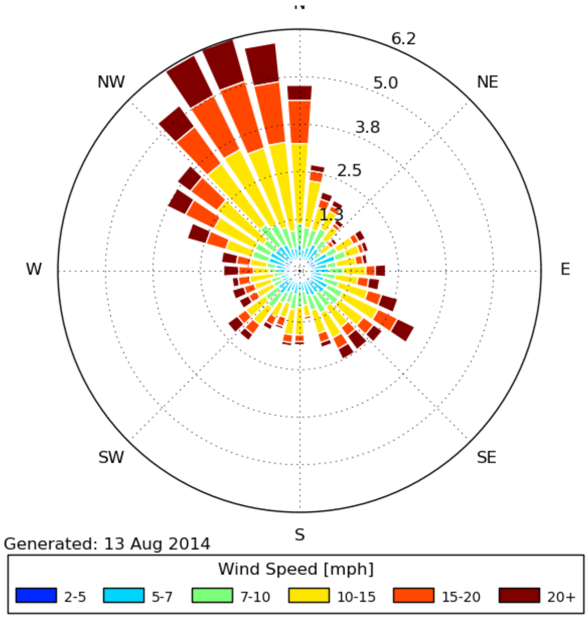

Figure 67 A wind rose for the airport of Lampedusa
(from http://mesonet.agron.iastate.edu/sites/windrose.phtml?station=LICG&network=IT__ASOS).

The land bridge connection with Tunisia during glacial periods could explain the presence of these species and lineages, but an in depth analysis also suggests a more complex explanation. A first clue is provided by the GLM indicating that the connection with Africa is not enough to explain the high richness showed by this island. If the "extra species" occurring on Lampedusa are examined, they are not low dispersive species as expected for glacial relicts. *Danaus chrysippus* and *Pontia* sp. are well known for their migratory behaviour. Erratic *Z. karsandra* individuals have been collected also on Malta and Sicily (Balletto et al., 2005), proving that occasional dispersing propagules can reach very distant areas. More likely, thanks to its very southern location, this island offers a unique opportunity among the circum-Sicilian islands for these north African elements to reproduce. The examination of the genetic richness confirms this hypothesis of ongoing dispersal to Lampedusa, since almost all the diversified species we analyzed from this island showed a relatively high number of haplotypes (*P. machaon*, *P. daplidice*, *P. celina*). *Papilio machaon* has a particularly high genetic variability, with four haplotypes in this island. Current data indicate that between Lampedusa and Tunisia there is a reduced, unidirectional gene flow but, it is still unknown if the observed pattern is due to insufficient sampling of the populations from Tunisia and Algeria, or if the diverged haplotypes on Lampedusa represent endemic or relict lineages. These characteristics make the population of *P. machaon* on Lampedusa of high conservation value.

References

Aistleitner, E. & Aistleitner, U. (2001) Weitere Notizen zur Lepidopterenfauna von Pantelleria, den Pelagischen und Ägadischen Inseln (Italien, Sizilien). Zeitschrift der Arbeitsgemeinschaft Österreichischer Entomologen, 53, 93-100.

Asher, J., Warren, M., Fox, R., Harding, P., Jeffcoate, G. & Jeffcoate, S. (2001) The millennium atlas of butterflies in Britain and Ireland. Oxford University Press.

Balletto, E., Toso, G. & Troiano, G. (1981) *Aricia cramera* (Erschscholtz, 1821) in Sardinia (Lycaenidae, Plebejinae). Nota Lepidopterologica, 4, 81-92.

Balletto, E., Bonelli, S. & Cassulo, L. (2005) Checklist e distribuzione della fauna italiana. 10.000 specie terrestri e delle acque interne: Insecta Lepidoptera Papilionoidea (Rhopalocera). Memorie del Museo civico di Storia naturale di Verona, 16, 259-263.

Biermann, H. (2005) Schmetterlinge der Liparischen Inseln. Atalanta, 36, 116-118.

Boillat, H. (2002) *Coenonympha lyllus* Esper, 1805, spec. Rev. Une nouvelle approche taxinomique du complexe pamphilus (Lepidoptera Nymphalidae Satyrinae). Alexanor, 22, 243– 309.

Bolotov, I.N., Podbolotskaya, M.V., Kolosova, Y.S. & Zubrii, N.A. (2013) The current flow of migrants and its contribution to butterfly faunas (Lepidoptera, Rhopalocera) on marine islands with young allochthonous biota. Biology Bulletin, 40, 78-88.

Calanchi, N., Lo Cascio, P., Lucchi, F., Rossi, P. L. & Tranne, C.A. (2007) Guida ai vulcani e alla natura delle Isole Eolie. LAC, Firenze.

Calcara, P. (1842) Descrizione dell'isola di Ustica. Giornale Letterario, 229, 64 pp.

Carroll, F. A., Hunt, C. O., Schembri, P. J. & Bonanno, A. (2012) Holocene climate change, vegetation history and human impact in the Central Mediterranean: evidence from the Maltese Islands. Quaternary Science Reviews, 52, 24-40.

Cesaroni, D., Lucarelli, M., Allori, P., Russo, F. & Sbordoni, V. (1994) Patterns of evolution and multidimensional systematics in graylings (Lepidoptera: *Hipparchia*). Biological Journal of the Linnean Society, *52*(2), 101-119.

Clement, M., Snell, Q., Walke, P., Posada, D. & Crandall, K. TCS: estimating gene genealogies. *Proc 16th Int Parallel Distrib Process Symp* **2**, 184 (2002).

Cucuzza, G. (1998) Discovery of a reproductive colony of *Danaus chrysippus* in eastern Sicily (Lepidoptera Danaidae). Bollettino della Societa Entomologica Italiana, 130, 273-276.

Dapporto, L. (2010) Satyrinae butterflies from Sardinia and Corsica show a kaleidoscopic intraspecific biogeography (Lepidoptera, Nymphalidae). Biological Journal of the Linnean Society, 100(1), 195-212.

Dapporto, L., Bruschini, C., Dincă, V., Vila, R. & Dennis, R.L.H. (2012) Identifying zones of phenetic compression in West Mediterranean butterflies (Satyrinae): refugia, invasion and hybridization. Diversity and Distributions, 18, 1066-1076.

Dapporto, L., Fattorini, S., Vodă, R., Dincă, V. & Vila, R. (2014a) Biogeography of western Mediterranean butterflies: combining turnover and nestedness components of faunal dissimilarity. Journal of Biogeography, 41, 1639-1650.

Dapporto, L., Vodă, R., Dincă, V. & Vila, R. (2014b) Comparing population patterns for genetic and morphological markers with uneven sample sizes. An example for the butterfly *Maniola jurtina*. Methods in Ecology and Evolution, 5, 834-843.

Dincă V., Dapporto L. & Vila R. (2011) A combined genetic-morphometric analysis unravels the complex biogeographical history of *Polyommatus icarus* and *Polyommatus celina* Common Blue butterflies. Molecular Ecology, 20, 3921-3935.

Dincă V., Montagud S., Talavera G., Hernández-Roldán J., Munguira M.L., García-Barros E., Hebert P.D.N. & Vila R. (2015) DNA barcode reference library for Iberian butterflies enables a continental-scale preview of potential cryptic diversity. Scientific Reports, 5, 12395.

Geiger, H., Descimon, H. & Scholl, A. (1988) Evidence for speciation within nominal *Pontia daplidice* (Linnaeus, 1758) in southern Europe (Lepidoptera: Pieridae). Nota lepidopterologica, 11, 7-20.

Haahtela T., Saarinen K., Ojaleinen P. & Aarnio H. (2011) Butterflies of Britain and Europe. A photographic guide. A&C Black Publishers Ltd, London.

Habel, J.C., Dieker, P. & Schmitt, T. (2009) Biogeographical connections between the Maghreb and the Mediterranean peninsulas of southern Europe. Biological Journal of the Linnean Society, 98, 693-703.

Habel, J.C., Husemann, M., Schmitt, T., Dapporto, L., Rödder, D. & Vandewoestijne, S. (2012) A forest butterfly in Sahara desert oases: isolation does not matter. Journal of Heredity, ess092.

Higgins, L.G. & Riley, N.D. (1970) A field guide to the butterflies of Britain and Europe. 1st American edn. Houghton Mifflin, Boston.

Higgins, L.G. (1975) The classification of European butterflies. Collins, London.

John, E., Cottle, N., McArthur, A. & Makris, C. (2008) Eastern Mediterranean migrations of *Pieris rapae* (Linnaeus, 1758)(Lepidoptera: Pieridae): observations in Cyprus, 2001 and 2007. Entomologist's Gazette, 59, 71.

John, E., Wiemers, M., Makris, C. & Russell, P. (2013) The *Pontia daplidice* (Linnaeus, 1758)/*Pontia edusa* (Fabricius, 1777) complex (Lepidoptera: Pieridae): confirmation of the presence of *Pontia daplidice* in Cyprus, and of *Cleome iberica* DC. as a new host-plant for this species in the Levant. Entomologist's Gazette, 64, 69-78.

Kreuzinger, A.J., Fiedler, K., Letsch, H. & Grill, A. (2015) Tracing the radiation of *Maniola* (Nymphalidae) butterflies: new insights from phylogeography hint at one single incompletely differentiated species complex. Ecology and Evolution, 5, 46-58.

Kudrna, O. & Leigheb, G. (1988) On the butterflies (Lepidoptera: Rhopalocera) of some Tyrrhenian islands (southern Italy). British Journal of Entomology and Natural History, 1, 133-137.

Kudrna, O., Harpke, A., Lux, K., Pennerstorfer, J., Schweiger, O., Settele, J. & Wiemers, M. (2011) The Distribution Atlas of Butterflies in Europe (pp. i-iv). Halle: Gesellschaft für Schmetterlingsschutz eV.

Lohman, D.J., Peggie, D., Pierce, N.E. & Meier, R. (2008) Phylogeography and genetic diversity of a widespread Old World butterfly, *Lampides boeticus* (Lepidoptera: Lycaenidae). BMC evolutionary biology, 8, 301.

Pisciotta, S., Zito, P. & Sajeva, M. (2008) *Danaus chrysippus* (Linnaeus, 1758) (Lepidoptera Nymphalidae) larvae feeding on *Caralluma europaea* (GUSS.) (Asclepiadaceae) in Lampedusa island. Naturalista siciliano, 32, 241-251.

Romano, F.P. & Romano, M., (1995) Lepidoptera. Naturalista sicil. 693-722 (suppl.).

Sammut, P.M. (1984) A Systematic and Synonymic List of the Lepidoptera of the Maltese Islands. Neue Entomologische Nachrichen, 13, 1-124.

Sammut, P. & Borg, J.J. (2008) An annotated catalogue of the Lepidoptera collection of Guido Lanfranco at the National Museum of Natural History in Malta. Bulletin of the Entomological Society of Malta, 1, 67-78.

Sañudo-Restrepo, C.P., Dincă, V., Talavera, G. & Vila, R. (2013) Biogeography and systematics of *Aricia* butterflies (Lepidoptera, Lycaenidae). Molecular Phylogenetics and Evolution, 66, 369-79.

Seizmair, M. (2014) Einige Erstmeldungen zur Rhopalocera-Fauna der Insel Ustica. Atalanta, 45, 199-201

Stefanescu, C., Penuelas, J. & Filella, I. (2003) Effects of climatic change on the phenology of butterflies in the northwest Mediterranean Basin. Global Change Biology, 9, 1494-1506.

Tarrier, M. & Delacre, J. (2008) Les Papillons de jour du Maroc. Guide d'identification et de bio-indication. Biotope, Meze, Muséum national d'Histoire naturelle, Paris.

Tennent, J. (1996) The butterflies of Morocco, Algeria and Tunisia. Gem Pub. Co..

Thomson, G. (2011) The Meadow Brown Butterflies: A Study in Genetics, Morphology and Evolution. George Thomson.

Tolman, T. & Lewington, R. (2008) Collins Butterfly Guide. Harper-Collins Publishers, London.

Tshikolovets, V.V. (2011) Butterflies of Europe and the Mediterranean area. Tshikolovets Publications.

Valletta, A. (1961) *Lycaenopsis argiolus* L., in Malta. The Entomologist, 94, 127.

Vodă, R., Dapporto, L., Dincă, V. & Vila, R. (2015) Why do cryptic species tend not to co-occur? A case study on two cryptic pairs of butterflies. PLoS One 10(2): e0117802.

Wagener, P.S. (1988) What are the valid names for the two genetically different taxa currently included within *Pontia daplidice* (Linnaeus, 1758)? (Lepidoptera: Pieridae). Nota lepidopterologica, 11, 21-38.

Weingartner, E., Wahlberg, N. & Nylin, S. (2006) Speciation in *Pararge* (Satyrinae: Nymphalidae) butterflies–North Africa is the source of ancestral populations of all *Pararge* species. Systematic Entomology, 31(4), 621-632.

Wiemers, M., Stradomsky, B.V. & Vodolazhsky, D.I. (2010) A molecular phylogeny of *Polyommatus* s. str. and *Plebicula* based on mitochondrial COI and nuclear ITS2 sequences (Lepidoptera: Lycaenidae). European Journal of Entomology, 107, 325-336.

Zavattari, E. (1960) Biogeografia delle Isole Pelagie. Rendiconti dell'Accademia Nazionale dei Lincei, 11.
